# *N*‑Chlorination of Sulfonamides: DFT Study of the Reaction Mechanism

**DOI:** 10.1021/acs.jpca.5c05059

**Published:** 2025-12-09

**Authors:** Antonio Ljulj, Petra Škibola, Dora Turkalj, Valerije Vrček

**Affiliations:** 87162 University of Zagreb, Faculty of Pharmacy and Biochemistry, Ante Kovačića 1, Zagreb 10000, Croatia

## Abstract

Sulfonamides are frequently detected as emerging pollutants in an aqueous environment. In wastewaters, their chemical fate is affected by a reaction with a chlorinating agent. In this study, the *N*-chlorination of sulfonamides has been explored computationally using density functional theory. This oxidation is the initial step that triggers other rearrangements of the parent structure. All sulfonamides contain three or more nitrogen atoms, which may serve as chlorination sites. According to the calculated results, the aniline moiety is the most reactive site, which supports the experimental findings. The observed regioselectivity of *N*-chlorination in sulfonamides has been interpreted in terms of kinetic and thermodynamic profiles of the reaction. It is demonstrated that protonation states and tautomer forms of sulfonamides should be considered to accurately calculate the mechanism underlying the chlorination. In a neutral aqueous medium, only the anion is reactive species, whereas in an acidic medium, both neutral form and its tautomers may react with the chlorinating agent. Along with the *N*-chlorinated intermediate, quantum chemical calculations have been employed to describe the formation of the ring-chlorinated product, which is frequently observed as a by-product during chlorination of sulfonamides.

## Graphical abstract

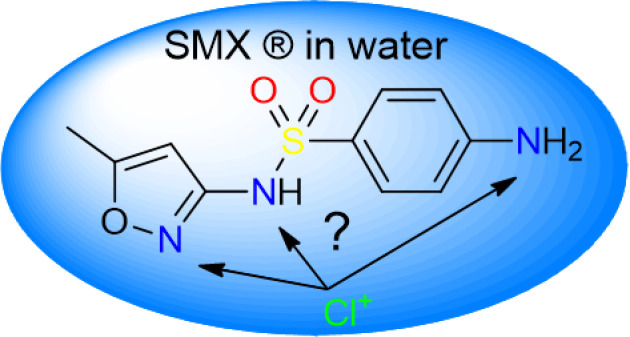

## Introduction

The chlorination of sulfonamides is of particular environmental importance, and literature reports are full of experimental data: reaction rate constants are measured, oxidation products detected, and ecotoxicity assessed.^1–3^ It is expected that all sulfonamides undergo similar chlorination reactions, i.e., the general mechanism of this process may be operative.^4^ This is, however, not fully confirmed, and details, e.g., reactive sites, the effects of chlorinating species, solvent, and pH, are still missing. A large collection of measured data available allows researchers to probe if computational chemistry methods can correctly reproduce these experiments. It is always of interest to establish a quantum chemical approach with high predictive power. To our surprise, theoretical protocols for the description of kinetic and thermodynamic profiles of chlorination of sulfonamides are rather scarce. There are some recent studies in which computational techniques have been employed to predict energy barriers for chlorination of sulfonamides^5^ and to locate (primary) reactive sites for chlorination.^6^ In both cases, the calculated data deviate a lot from experimental values, which emphasizes the need for further work in the computational description of sulfonamide chlorination.

One critical issue, not resolved earlier, is the primary site of *N*-chlorination. It has been shown that N-Cl intermediates are transient and important species formed during the *N*-chlorination of sulfonamides.^4^ All sulfonamides contain three types of nitrogen atoms, which are possible targets for the chlorine attachment: aromatic amine, sulfonamide, and heterocycle nitrogen atoms. To determine the kinetic and/or thermodynamic control of these (three) competitive routes, energy barriers (Δ*G* ^‡^) and relative stabilities of the corresponding *N*-chlorinated products should be correctly estimated and compared. For this protocol, the structure of sulfamethoxazole (SMX) has been selected as a relevant model, containing the common motif of 4-aminobenzenesulfonamide and the heterocycle (isoxazole ring) part attached to it. The most comprehensive experimental report on chlorination of SMX has been published earlier^7^ and may serve as a checkpoint for assessing the efficacy and accuracy of computational methods. In this landmark study, Dodd and Huang described the kinetics and proposed the mechanism and possible pathways of the reaction between HOCl and SMX. In specific, they applied a substructural model approach to identify the reaction centers. In this work, we try to reproduce their experimental results and to provide a full description of regioselectivity observed in the chlorination of SMX.

## Computational Details

All calculations were performed using the Gaussian suite of programs (version 16.C01)^8^ using the advanced computing service (cluster Supek) provided by the University of Zagreb University Computing Centre (SRCE)^9^ and the computational resources of the PharmInova project (sw.pharma.hr) at the University of Zagreb Faculty of Pharmacy and Biochemistry.^10^

All structures were fully optimized with the hybrid B3LYP functional.^11^ The standard split valence and polarized 6-31+G­(d,p) basis set, with diffuse functions added, was used for geometry optimizations and frequency calculations. The B3LYP/6-31+G­(d,p) method appears to be very accurate in reproducing the experimental data, e.g., the measured energy barrier for *N*-chlorination of sulfamethoxazole, and relative stabilities of sulfamethoxazole tautomers. The selected DFT method is similar to the level of theory reported earlier (B3LYP/6-311G­(d,p) level),^5^ which makes these results comparable.

All energies in the main text are reported at 298.15 K. Calculated energy barriers for *N*-chlorination reactions and the regioselectivity pattern at elevated temperatures are reported in Table S2. Thermal corrections to Gibbs free energies were calculated at the same level using the rigid rotor/harmonic oscillator model. Analytical vibrational analyses were performed to characterize each stationary point as a minimum (NImag=0) or transition state (NImag=1). Intrinsic reaction coordinate calculations were performed to identify the minima connected through the transition state.

The improved energies have been calculated at the M06-2X/6-311+G­(d,2p) level with the empirical dispersion correction (the D3 version of Grimme’s dispersion).^12^ Gibbs free energies were obtained by including thermal corrections calculated at the B3LYP/6-31+G­(d,p) level (denoted as M06-2X/B3LYP in the text).

Gibbs energies of solvation were calculated using the SMD solvation model at the B3LYP/6-31+G­(d,p) level.^13^ The solvent relative permittivity of ϵ = 78.4 (water) was used. To describe sulfonamides in water, the inclusion of bulk (continuum) and specific solvent effects has been explored. We have found that the addition of explicit water molecules substantially lowers the calculated energy barriers for *N*-chlorination process, tautomerization, and Orton-like rearrangement in sulfamethoxazole (see SI).

Gibbs free energy barriers (Δ*G* ^‡^) have been calculated as the relative energy difference between the transition state structure and reactants (sulfonamide anion and HOCl). The correction term (≈−10 kJ/mol) was included to evaluate the effect of the loss of translation degrees of freedom in solution on the Gibbs activation energy in bimolecular reactions, as reported by Ardura et al.^14^ The chlorinating species was complexed with a given number of water molecules (*n* = 0–3), and Gibbs reaction energies (Δ*G*_r_) have been calculated as an energy difference between the water-complexed reactants and products. The same methodology was applied in earlier theoretical studies of relevant water-assisted processes, which ensured that reactants, transition states, and products pertain to the same minimum-energy path.^15^

The half-lives (*t*_1/2_) reported in the text were obtained from rate constants (*t*_1/2_ = ln 2/*k*) calculated using the Eyring equation $k=\frac{k_{B}T}{h}e^{-\Delta G^{‡}/RT}$ , where *T* = 298.15 K, RT = 2.478 kJ/mol, and *k*_B_ *T*/*h* = 6.212 × 10^12^ s^–1^ (κ = 1). For comparison of intrinsic reactivity, pseudo-first-order rate constants and the corresponding half-lives should be regarded as illustrative values derived under the standard pseudo-first-order approximation for a fundamentally second-order process.

The most stable water-complexed stationary points (reactants, transition states, and products) have been located by a stochastic search procedure.^16,17^ This procedure, as reported earlier,^18,24^ provides a relatively quick screen of all possible configurations of waters around the respective structure.

## Results and Discussion

Out of three possible *N*-chlorinated products **SMX-ISX-Cl**, **SMX-SO**_**2**_ **NCl**, and **SMX-PhNCl** (Scheme 1), only the aromatic amine-chlorinated product (**SMX-PhNCl**) was detected experimentally.^7,19,20^ It is known, however, that aromatic amines (i.e., substituted anilines) do not undergo *N*-chlorination easily due to low reactivity of the nitrogen atom toward HOCl.^21^ Delocalization of the electron pair on nitrogen by the resonance effect increases the energy barrier (Δ*G* ^‡^ > 150–300 kJ/mol) for nucleophilic attack of the amine group, which makes the reaction with chlorine kinetically prohibitive.^22^ The calculated energy barrier of *N*-chlorination reactions for some aromatic amines may be lowered by the addition of explicit water molecules, but not sufficiently for the reaction to occur.^22^

**Scheme 1:**
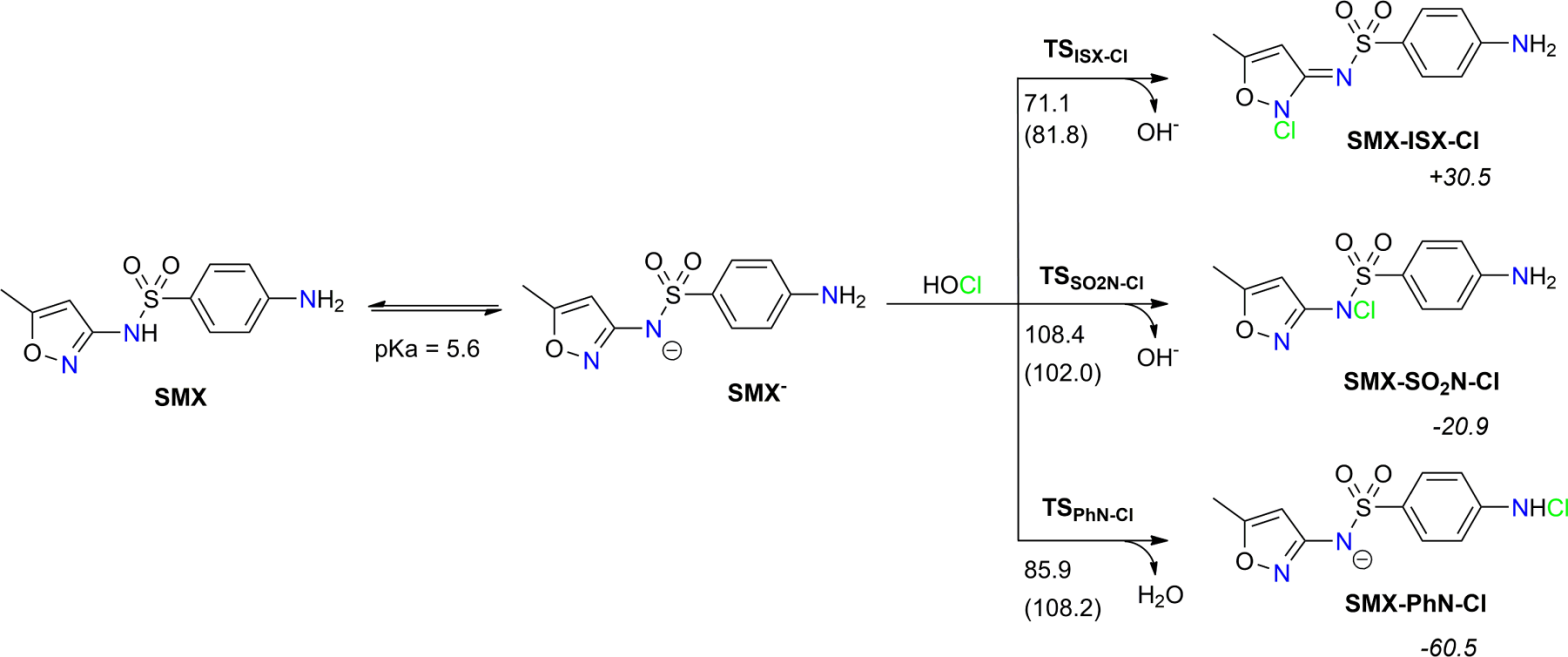
*N*-Chlorinated Products (**SMX-ISX-Cl**, **SMX-SO**_**2**_ **N-Cl**, and **SMX-PhN-Cl**) of Reactions between Anionic Form of Sulfamethoxazole **SMX** ^–^ and HOCl[Fn sch1-fn1]

i The reaction energies (Δ*G*_r_) are in italics, and energy barriers (Δ*G* ^‡^) for each respective pathway are placed below reaction arrows (corresponding barriers for chlorination of neutral **SMX** are in parentheses). All values (in kJ/mol) were calculated at the M06-2X­(D3)/6-311+G­(d,2p)//B3LYP/6-31+G­(d,p) level. Three explicit water molecules (not shown for clarity) were included in calculations of all stationary points (minima and transition states).

In contrast to that of anilines, the aromatic amine moiety in sulfomethoxazole may be effectively *N*-chlorinated. This is due to the acidic sulfonamide NH group (p*K*_a_ = 5.6), which is mostly deprotonated in a neutral aqueous environment. According to the Henderson–Hasselbalch equation, the fraction (*f*_SMX_-) of the anionic form is over 96%, and the neutral form (*f*_SMX_) makes less than 4% at pH = 7 (for details, see SI, pg. S2). The anionic form **SMX** ^–^ is not only predominant species but also more reactive toward HOCl. This was observed experimentally^7^ and supported by our calculations herewith. The measured rate constant for the reaction between HOCl and sulfamethoxazole anionic form is two times higher than the corresponding value for neutral species.^3^ In agreement with this, the calculated energy barrier (Δ*G* ^‡^ = 85.9 kJ/mol) for chlorination of the aromatic amine in **SMX** ^–^ is >30 kJ/mol lower than the barrier for the corresponding reaction in the neutral **SMX** (Scheme 1). The same applies to chlorination of *N* atoms in sulfonamide and isoxazole moieties: the calculated barriers are lower when the anionic form **SMX** ^–^ is considered as a reactant. It comes out that all *N*-chlorination reactions in sulfamethoxazole are modulated by the ionization state of the sulfonamide group.

According to our calculations, the *N*-chlorination of the sulfonamide group is a prohibitive or very slow process due to a high energy barrier (Δ*G* ^‡^ = 108.4 kJ/mol; half-life (*t*_1/2_) = 12.6 days). Therefore, the **SMX-SO**_**2**_ **N-Cl** is a kinetically disfavored product. The *N*-chlorination of the isoxazole group is kinetically preferred (Δ*G* ^‡^ = 71.1 kJ/mol), but the reaction is very endergonic (Δ*G*_r_ = +30.5 kJ/mol), resulting in unstable chlorinated product **SMX-ISX-Cl**. The latter is converted back to the starting **SMX** ^–^ or transforms (via an intramolecular chlorine shift) to the more stable product **SMX-PhN-Cl** (see below). In contrast, the chlorination of the aromatic amine group is a strongly exergonic process (Δ*G*_r_ = −60.5 kJ/mol), in which the **SMX-PhN-Cl** is a thermodynamically favored product. The calculated barrier for this process is slightly higher (Δ*G* ^‡^ = 85.9 kJ/mol) but is easily overcome at room temperature (half-life (*t*_1/2_) = 2.1 min).

The chlorination site, therefore, is not the sulfonamide nitrogen atom or isoxazole moiety but the aromatic amine nitrogen. The selected computational model accurately reproduced the experimental kinetic profile of chlorination of sulfamethoxazole. A half-life (*t*_1/2_) of 23 seconds was measured under pseudo-first order conditions, which corresponds to reaction rate constant *k*_r_ = 0.03 s^–1^ and Gibbs free energy of activation Δ*G* ^‡^ = 82 kJ/mol. This value, derived from the Eyring equation, was correctly reproduced computationally at the M06-2X/6-311+G­(d,2p)//B3LYP/6-31+G­(d,p) level (Δ*G* ^‡^ = 85.9 kJ/mol), but only if the anionic pathway **SMX** ^–^ → **SMX-PhN-Cl** was considered. The corresponding transition state structure **TS**_**PhN‑Cl**_ (Figure 1) is characterized by a cyclic arrangement in which three water molecules assist intramolecular proton transfer between HOCl and aromatic amine.^23^ The six-membered planar ring (only heavy-atom count) is a typical structural motif described earlier for *N*-chlorination of a series of aliphatic and aromatic amines.^24,25^ On the other side, the energy barrier for the pathway **SMX** → **SMX-PhN-Cl**, via transition state **TS’**_**PhN‑Cl**_ (see Figure S1), is higher than 100 kJ/mol (Scheme 1, in parentheses), which makes the neutral reaction channel less preferred.

**Figure 1:**
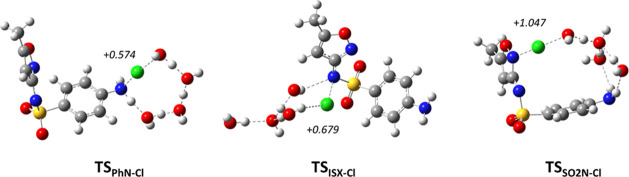
B3LYP/6-31+G­(d,p) optimized transition state structures for the *N*-chlorination of sulfamethoxazole anionic species **SMX** ^–^ at different nitrogen atoms. The calculated APT charges (*q*_Cl_) of the transferred chlorine atom are shown in italics.

In all three transition state structures **TS**_**PhN‑Cl**_, **TS**_**ISX‑Cl**_, and **TS**_**PhSO2N‑Cl**_ (Figure 1), the three explicit water molecules participate in forming the network (dashed lines) which facilitates Cl^+^/H^+^ transfers.^26^ The nature of the chlorine atom transferred in the course of the reaction may be described by partial atomic charge analysis. Accordingly, the APT charges (*q*_Cl_) have been calculated for transition state structures (Figure 1). It is known that these atomic polar tensor charges are less dependent on the DFT functional used.^27^ High values of *q*_Cl_ were calculated for the Cl atom in corresponding transition states, suggesting that the transfer of cationic species (Cl^+^) is operative in all three *N*-chlorination pathways.

The alternative mechanism that may contribute to the formation of **SMX-PhN-Cl** includes chlorinated isoxazole intermediate **SMX-ISX-Cl** (Scheme 2). As noted earlier, it is an unstable intermediate (higher in energy), but its formation is kinetically favored (Δ*G* ^‡^ = 71.1 kJ/mol). It may undergo the intramolecular *N*,*N*-chlorine shift, a rearrangement via transition state structure **TS-NClN**, which is 113.5 kJ/mol less stable than starting **SMX-ISX-Cl**. The chlorine migration to the aromatic primary amine is followed by a rapid proton shift, resulting in the formation of thermodynamically favored product **SMX-PhN-Cl** (Δ*G*_r_ = −67.8 kJ/mol). No explicit water molecule is necessary to facilitate the chlorine shift, which makes this process feasible also in a non-aqueous environment or in a hydrophobic enzyme cavity. The analogous mechanism of intramolecular *N*,*N*-chlorine shift was reported earlier in the reaction between carnosine and HOCl.^28^ The authors proposed the importance of this long-range chlorine transfer in a wide range of *N*-chlorinated bioactive compounds. In this study, we probed the same mechanism in different sulfonamide structures (see below) and revealed that the heterocyclic moiety strongly influences the ease of the intramolecular *N*,*N*-chlorine transfer, i.e., the transfer may not be a feasible process in the whole sulfonamide family.

**Scheme 2:**
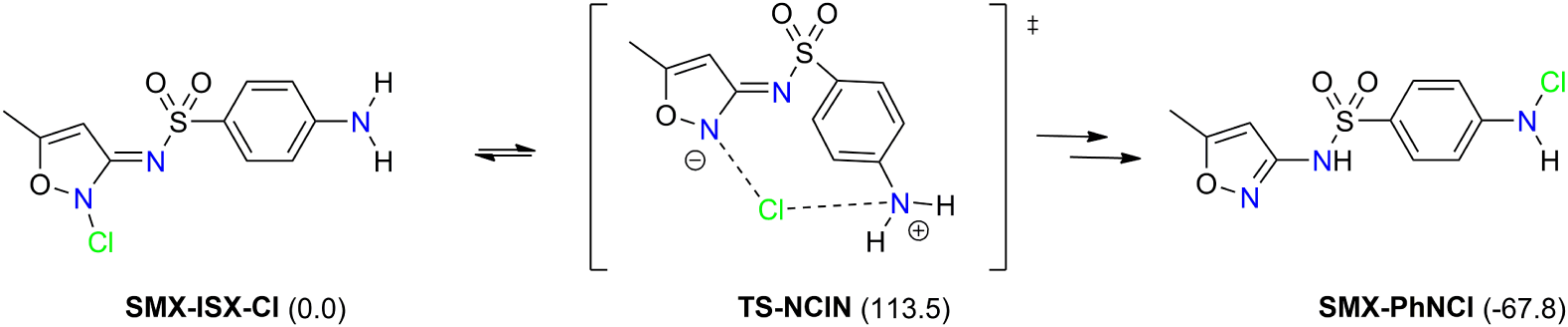
Intramolecular *N*,*N*-Chlorine Shift in *N*-Chlorinated Isoxazole Intermediate, Converting the Unstable **SMX-ISX-Cl** to **SMX-PhN-Cl** [Fn sch2-fn1]

i The relative energies (Δ*G*) are in parentheses. All values (in kJ/mol) were calculated at the M06-2X­(D3)/6-311+G­(d,2p)//B3LYP/6-31+G­(d,p) level.

To conclude this part, our results indicate that speciation (protonation state) of sulfamethoxazole is of utmost importance for computational settings and for the correct presentation of the mechanism underlying the chlorination of the sulfonamide. This is probably why in previous works the reported energy barriers for *N*-chlorination of sulfamethoxazole and other sulfonamides were extremely high and kinetically prohibitive (all calculated Δ*G* ^‡^ > 146 kJ/mol, which corresponds to a half-life > 12 000 years).^5^ This also may explain inaccurate chlorination sites predicted earlier by computational results.^6^ In addition, we show that inclusion of two explicit water molecules is mandatory for an accurate description of transition state structures involved in corresponding *N*-chlorination reactions (Figure 1), whereas the alternative mechanism (intramolecular *N*,*N*-chlorine migration; Scheme 2) does not require the assistance of water molecules.

Along with the parent (neutral) sulfamethoxazole, we have calculated the *N*-chlorination pathway which includes its tautomeric imide forms **SMX-imide-Z** and/or **SMX-imide-E** (Scheme 3). It is known that, for example, amide-containing pharmaceuticals are converted to more reactive imide tautomers, which undergo *N*-chlorination more easily.^29^ In addition to the *E*- and *Z*-isomers of the sulfonimide, the enol tautomer **SMX-enol** was also considered (Scheme 3). However, it is very unstable (easily converted to **SMX-imide-Z**, Δ*G* ^‡^ 64.3 kJ/mol), and its contribution to the *N*-chlorination process is negligible. In agreement with earlier reports,^30,31^ the *E*- and *Z*-forms of sulfonimide are similar in energy, the latter being somewhat more stable due to the intramolecular NH...O=S hydrogen bond.^32^ Both forms exist in a fast equilibrium driven by rotation around a partially double CN bond (Δ*G* ^‡^ for the interconversion **SMX-imide-Z** → **SMX-imide-E** is 75.7 kJ/mol).

**Scheme 3:**
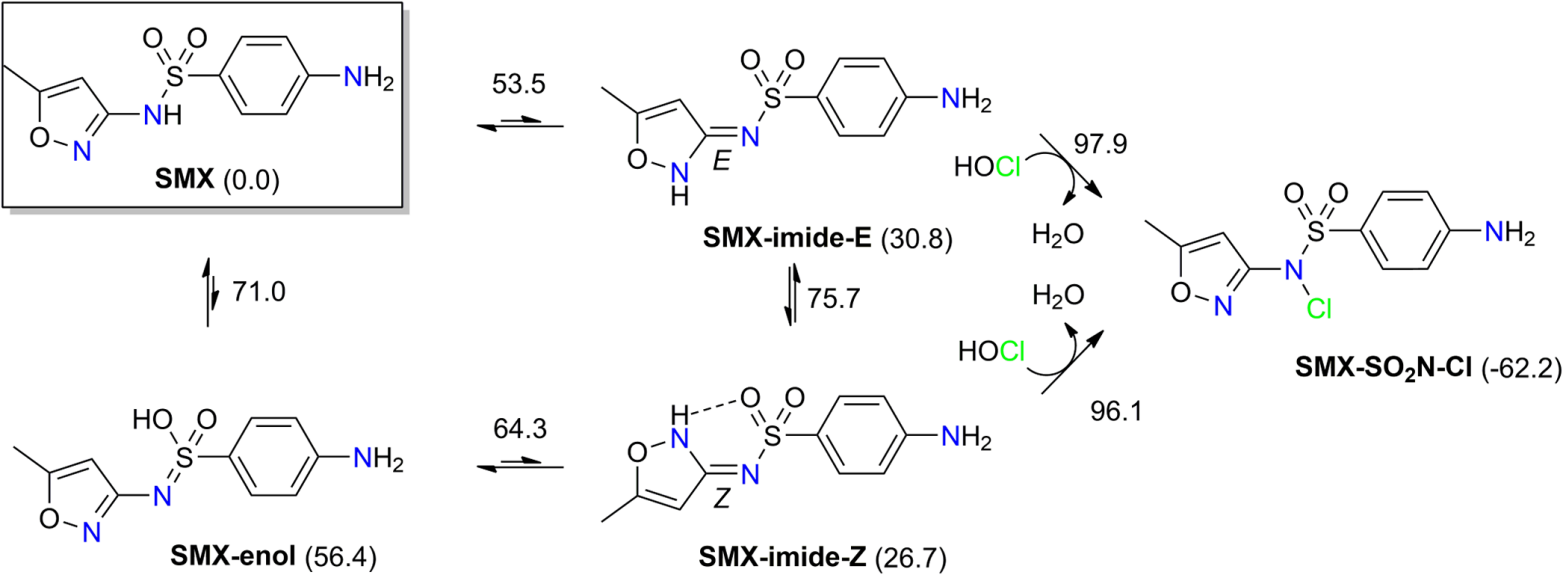
Sulfamethoxazole (**SMX**) and Its Tautomeric Sulfonimide (**SMX-Imide-Z**, **SMX-Imide-E**) and Enol (**SMX-Enol**) Forms[Fn sch3-fn1]

i Relative energies (Δ*G*_r_) are in parentheses, and energy barriers (Δ*G* ^‡^) are placed on the respective reaction arrow. All values (in kJ/mol) were calculated at the M06-2X­(D3)/6-311+G­(d,2p)//B3LYP/6-31+G­(d,p) level. Three explicit water molecules were included (not shown for clarity) in calculations of all stationary points (minima and transition states).

According to calculated energy barriers, both *E*- and *Z*-sulfonimides are of similar reactivity toward HOCl. As expected, the tautomers are more reactive than the parent neutral sulfonamide **SMX**. Due to the increased nucleophilicity of the nitrogen atom,^29^ the −N–S­(O_2_)– moiety is the primary chlorination site in these tautomers, i.e., the formation of chlorinated product **SMX-SO**_**2**_ **N-Cl** is kinetically favored (Δ*G* ^‡^ = 96.1 kJ/mol, Scheme 3). In the case of the parent **SMX**, see above, the *N*-chlorination of the isoxazole moiety is kinetically favored (Δ*G* ^‡^ = 81.8 kJ/mol, Scheme 1), whereas the anionic form **SMX** ^–^ undergoes aromatic amine *N*-chlorination (Δ*G* ^‡^ = 85.9 kJ/mol, Scheme 1). It is a good illustration how the chlorinated product distribution depends on the ionization state and/or tautomeric form of the reactant. In a neutral aqueous environment, only the anionic pathway is relevant. At pH ≈ 7, the equilibrium fraction of neutral **SMX** (p*K*_a_ = 5.6) and its sulfonimide isomers is very small, and their contribution to the overall kinetics is not important (see details in SI, Table S1). However, in the acidic medium, the neutral forms, **SMX**, **SMX-imide-Z**, and **SMX-imide-E**, may appear as reactive species.

It is interesting to note that the sulfonamide-chlorinated (**SMX-SO**_**2**_ **N-Cl**) product was not detected in experimental studies.^19,33–36^ The chlorination of sulfamethoxazole, under various reaction conditions, resulted in the formation of an aromatic *N*- or *C*-chlorinated product only. As described herewith, the chlorination of sulfamethoxazole includes its anionic form, **SMX** ^–^, in which the preferential chlorination site is the aromatic nitrogen atom.

To explain the formation of the *C*-chlorinated product (**SMX-PhC-Cl**) in the reaction between sulfamethoxazole and HOCl, several pathways were considered computationally: direct *C*_ortho_ chlorination of **SMX**, intramolecular *N*,*N*-chlorine shift in **SMX-ISX-Cl** analogous to Cl-transfer reported for carnosine,^28^ and the intramolecular (*N* -> *C*_ortho_) 1,3-chlorine shift in **SMX-PhN-Cl** (see details in Scheme S1).

According to our results, the latter process is kinetically favored. It is an Orton-like rearrangement,^37^ which is important for chemical fate of SMX in aqueous environments and for metabolism of this drug in, for example, neutrophils and/or monocytes.^38^ It has been shown that chlorination of SMX catalyzed by myeloperoxidase results in **SMX-PhN-Cl**, which spontaneously rearranges to the *C*-chlorinated product **SMX-PhC-Cl**. In this work, we describe the mechanism underlying this reaction (Scheme 4). It is a two-step process in which the chlorine atom is transferred from nitrogen to the ortho-position of the aromatic ring. The transition state structure **TS_Cl**_**1**_ corresponds to the 1,3-chlorine shift, which is followed by fast proton transfer rearrangement **SMX-Cl**_**int**_ → **SMX-PhC-Cl**. The former reaction is a rate-determining step with a barrier of Δ*G* ^‡^ = 99.3 kJ/mol, which is readily achievable in a thermally induced Orton-like process.^39^ The same reaction may be acid-catalyzed or photoinduced,^40^ but these pathways are not operative under the experimental setup (as in LC/MS analysis).^7^ The intermediate **SMX-Cl**_**int**_ may undergo (de)­protonation, thus restoring its aromaticity and regenerating amine functionality. In any case, the rearrangement of *N*-chlorinated sulfamethoxazole is a thermodynamically driven reaction, which results in a more stable ring-chlorinated product. The net reaction energy (Δ*G*_r_ = −133.1 kJ/mol) is mostly determined by the difference between the average bond energies for the N–Cl (200 kJ/mol) and C–Cl (339 kJ/mol) bonds.

**Scheme 4:**
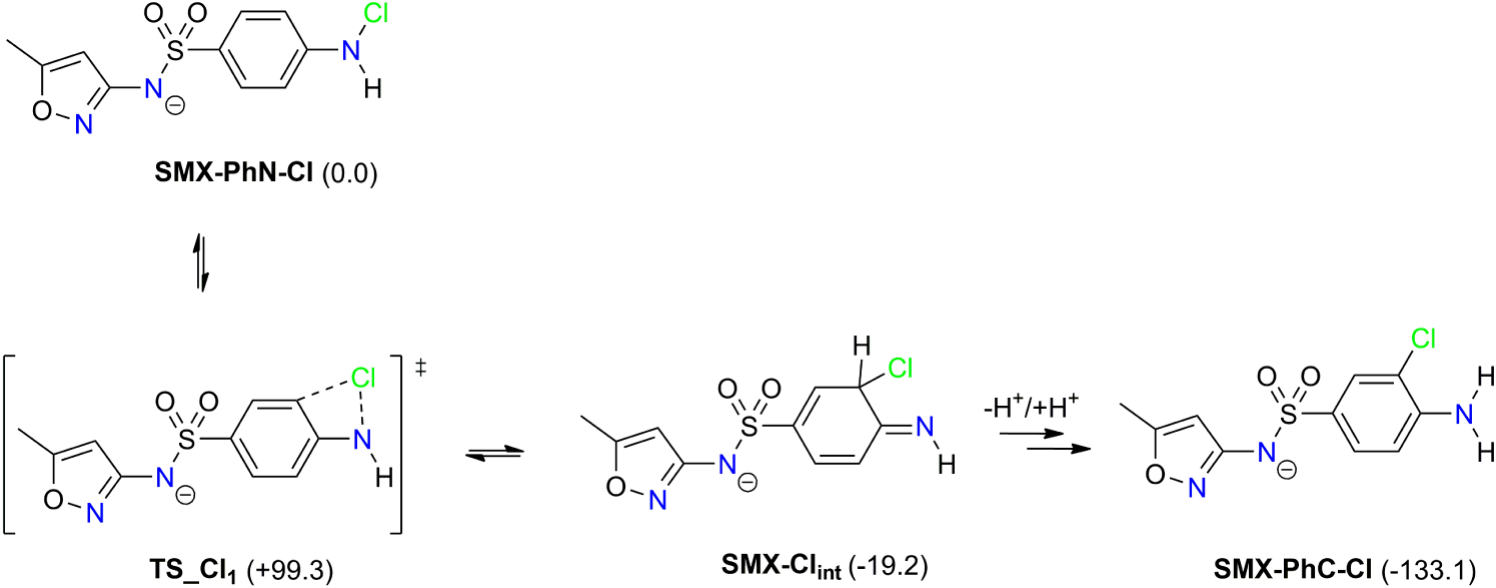
Reaction Mechanism of the Orton-like Transformation of *N*-Chlorinated Sulfamethoxazole (**SMX-PhN-Cl**) to Ring-Chlorinated Product (**SMX-PhC-Cl**)­[Fn sch4-fn1]

i Relative energies (Δ*G*_298_) are in parentheses. All values (in kJ/mol) were calculated at the M06-2X­(D3)/6-311+G­(d,2p)//B3LYP/6-31+G­(d,p) level of theory.

The *N*-chlorination step and the subsequent 1,3-chlorine shift were computationally evaluated in other sulfonamides, which are frequently detected in the aqueous environment.^41^ These include sulfadiazine (SDZ), sulfamethazine (SMZ), sulfathiazole (STZ), and sulfisoxazole (SIZ), for which both *N*- and *C*-chlorinated transformation products were reported. As expected, the kinetic and thermodynamic profiles of the two pathways were not altered by the presence of different heterocyclic rings in the sulfonamide structures (Table 1). All calculated energy barriers (Δ*G* ^‡^) for *N*-chlorination and 1,3-chlorine shift span a range of only 9 kJ/mol. It is well within the margin of error for the selected computational level. The same is observed for calculated reaction energies (Δ*G*_r_), which cover a 2 kJ/mol range. This suggests that the common mechanism, for both *N*-chlorination and *N,C*-chlorine shift, is operative throughout the entire family of sulfonamides.

**Table 1:**
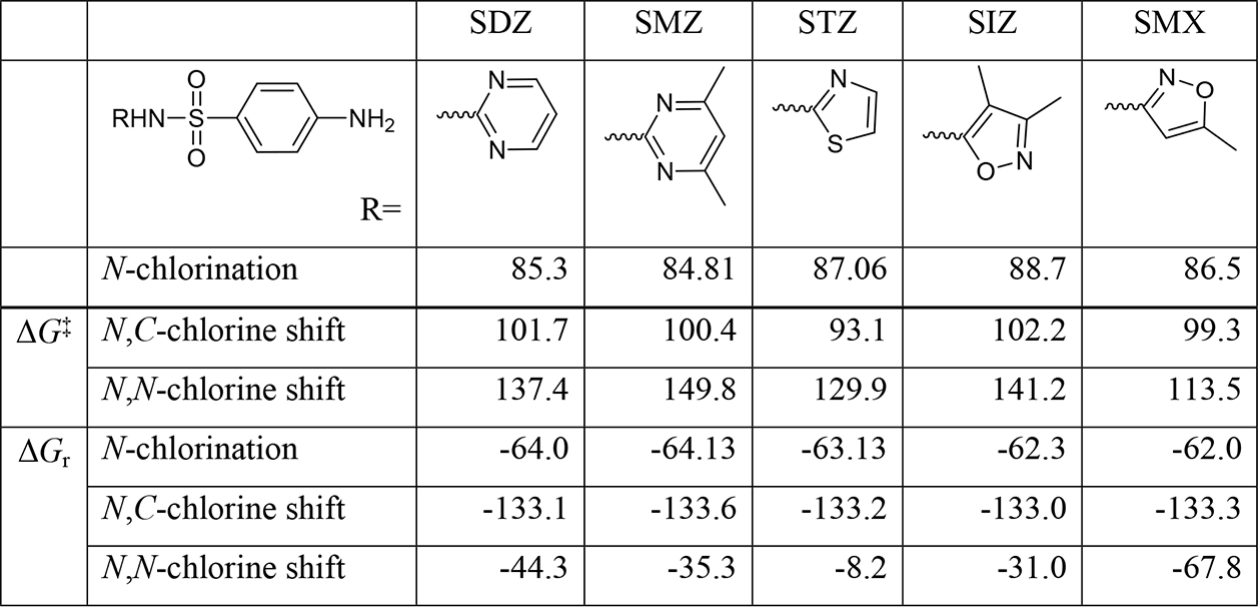
Calculated Gibbs Free Energy Barrier (Δ*G* ^‡^) and Gibbs Free Reaction Energies (Δ*G*_r_) for the *N*-Chlorination Reaction (at Aniline Nitrogen Position), *N*,*C*-Chlorine Shift (From Nitrogen to C_ortho_ Position), and *N*,*N*-Chlorine Shift in Different Sulfonamides^a^

a All values in kJ/mol, calculated at the M06-2X­(D3)/6-311+G­(d,2p)//B3LYP/6-31+G­(d,p) level of theory.

In contrast, kinetic and thermodynamic profiles of the intramolecular *N,N*-chlorine shift are more affected by the presence of different heterocylic rings. Both Δ*G* ^‡^ and Δ*G*_r_ values are within the ranges 36 and 60 kJ/mol, respectively. A much larger energy window is expected, as the heterocycle part is directly involved in the corresponding *N,N*-chlorine transfer reaction.

## Conclusions

The *N*-chlorination is a primary step in the reaction between sulfonamides and hypochlorous acid. It governs the subsequent transformations of these antibiotics in a chlorinated water environment. In this computational (DFT) work, sulfamethoxazole (**SMX**) has been selected as a model to explore the mechanism underlying the kinetics, regioselectivity, and thermodynamic profile of the reactions. In a neutral medium (pH ≈ 7), the chlorinating species reacts with the anionic form (**SMX** ^–^) of sulfamethoxazole (p*K*_a_ = 5.6), resulting in product **SMX-PhN-Cl** chlorinated at the aniline moiety. Out of three possible *N*-chlorinated intermediates, **SMX-ISX-Cl**, **SMX-SO**_**2**_ **N-Cl**, and **SMX-PhN-Cl**, the latter has been thermodynamically preferred. It may undergo an Orton-like rearrangement in which the chlorine atom is transferred from the *N*- to *C*_ortho_-position. This 1,3-chlorine shift results in the ring-chlorinated product **SMX-PhC-Cl**, which is, along with **SMX-PhN-Cl**, frequently observed as a transformation product of sulfamethoxazole in the aqueous environment.

The two respective reactions, aromatic amine chlorination and Orton-like rearrangement in **SMX**, were probed with other members of the sulfonamide family: sulfadiazine, sulfamethazine, sulfathiazole, and sulfisoxazole. No significant effect of the heterocyclic ring on the kinetic and/or thermodynamic profile was observed.

In order to correctly describe the chlorination profiles in sulfonamides, their ionization states and tautomeric forms should be considered, and explicit water molecules should be included in calculations. In the neutral medium, only the anionic form is relevant reactant species, whereas in the acidic medium, the neutral form and its two tautomers appear as reactive species.

## Supplementary Material



## Notes

Boltzmann distribution proportion values for sulfamethoxazole, its anionic form and tautomers, thermochemical and solvation parameters calculated at different temperatures, alternative reaction pathways, optimized coordinates, and their Gibbs free energies, thermal corrections, and solvation energies (PDF)
