# Supplementary material for: N‑Chlorination of Sulfonamides: DFT Study of the Reaction Mechanism
Source: J Phys Chem A. 2025 Dec 9;129(50):11495–501. doi: 10.1021/acs.jpca.5c05059 (PMC12720232; doi:10.1021/acs.jpca.5c05059)
Supplement: Supplementary file 1 [file jp5c05059_si_001.pdf]

SUPPORTING INFORMATION

N-Chlorination of sulfonamides. DFT study of the reaction mechanism.

Antonio Ljulj, Petra Škibola, Dora Turkalj, and Valerije Vrček*
University of Zagreb, Faculty of Pharmacy and Biochemistry, Ante Kovačića 1, 10000 Zagreb, Croatia

*Corresponding author:
Prof. dr. Valerije Vrček
Tel: +385-1-6394441
Fax: +385-1-4856201
E-mail: vvrcek@pharma.hr

Table of Contents

Figure S1. *N*-chlorination of sulfamethoxazole (**SMX**) and its anionic form (**SMX$^-$**)………..……………S2
Table S1. Boltzmann distribution proportion *f* values of SMX and its tautomers……………...…………..S3
Table S2. Thermochemical and solvation parameters calculated at different temperatures
Scheme S1. Different reaction pathways resulting in the formation of **SMX-PhC-Cl**……………………..S4
Coordinates, Gibbs free energies, Thermal corrections, and solvation energies calculated at the M06-2X(D3)/6-311+G(d,2p)//B3LYP/6-31+G(d,p) level……………………………………………….S6

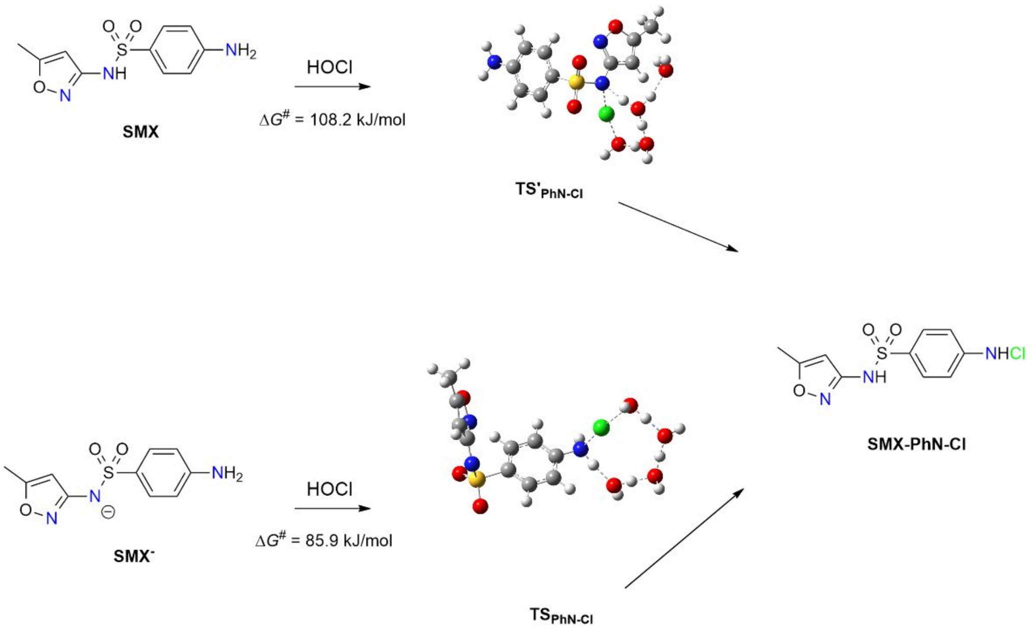

Figure S1. *N*-chlorination of sulfamethoxazole (**SMX**) and its anionic form (**SMX**$^-$). The corresponding transition state structures **TS'**$_{\mathbf{PhN\text{-}Cl}}$ and **TS**$_{\mathbf{PhN\text{-}Cl}}$, respectively, were optimized at the B3LYP/6-31+G(d,p) level of theory.

According to Eyring (transition state) theory the microscopic rates for anionic (k_{SMX}-) and neutral (k_{SMX}) pathways correspond to:

$$k = \frac{k_B \mathrm{T}}{h}\, e^{-\Delta G^{\ddagger}/RT}$$

where T = 298.15 K, RT = 2.478 kJ/mol, $k_B T$ / h= 6.212 x 10^{12} s^{-1} (if κ = 1), and

$\Delta G^{\ddagger}_{(\mathbf{SMX})}$ = 108.2 kJ/mol, $\Delta G^{\ddagger}_{(\mathbf{SMX}-)}$ = 85.9 kJ/mol

Neutral pathway: k_{SMX} = 6.88 x 10^{-7} s^{-1}

Anionic pathway: k_{SMX-} = 5.55 x 10^{-3} s^{-1}

In the neutral aqueous solution the equilibrium fractions (f) of the anionic (**SMX**$^-$) and neutral (**SMX**) species are:

$$f_{SMX^-} = \frac{1}{1 + 10^{(pKa - pH)}} = 0.9617, \qquad and\, f_{SMX} = 1 - f_{SMX^-} = 0.0383$$

where pK_a (**SMX**) = 5.6, and pH = 7.

Therefore, the apparent rate constant k_{app} is the fraction-weighted sum of the neutral and anionic pathway rate constants (k_{SMX} and k_{SMX-}):

$k_{app} = f_{SMX}\, k_{SMX} + f_{SMX-}\, k_{SMX-} = 5.29 \times 10^{-3}\ s^{-1}$

The effective "apparent barrier" from k_{app} is $\Delta G^{\ddagger}_{app} = 86.02$ kJ/mol, which is essentially the barrier for the anionic pathway. The analogous approach has been reported elsewhere.[i,ii]

Table S1. Boltzmann distribution proportion *f* values of **SMX** and its tautomers

isomer	ΔG (kJ/mol)[a]	Boltzmann fraction (f)[b]	Microscopic rate constant (k / s^{-1})[c]	Apparent rate constant (k_{app} / s^{-1})[d]	Apparent energy barrier ($\Delta G^{\ddagger}$ / kJ/mol)[e]	Reaction pathway contribution to k_{app} (%)
**SMX**	0	0.99998	8.388×10^{-6}	8.389×10^{-9}	102.0	99.975
**SMX-imide-Z**	26.7	2.1×10^{-5}	9.063×10^{-5}			0.023
**SMX-imide-E**	30.8	4.0×10^{-6}	4.385×10^{-5}			0.002

[a] Calculated at the M06-2X(D3)/6-311+G(d,2p)//B3LYP/6-31+G(d,p) level. [b] At 298.15 K. $RT = 2.479$ kJ/mol; $f_{SMX} = e^{0} / (e^{0} + e^{-26.7/RT} + e^{-30.8/RT})$. [c] *N*-chlorination reaction rate constant; from Eyring equation $k = (k_B T/h)e^{-\Delta G\ddagger/RT}$ ($\kappa = 1$), [d] $k_{app} = f_{SMX}\, k_{SMX} + f_{SMX\text{-imide-Z}}\, k_{SMX\text{-imide-Z}} + f_{SMX\text{-imide-E}}\, k_{SMX\text{-imide-E}}$, [e] $\Delta G^{\ddagger} = -RT \ln (k_{app}h/k_B T)$, [f] $\text{contribution}_i = f_i k_i / k_{app}$

In conclusion, the apparent energy barrier is identical to the **SMX** pathway, because **SMX** overwhelmingly dominates the equilibrium mixture despite the **SMX-imide-Z** and **SMX-imide-E** being more reactive (see microscopic rate constants).

Table S2. Thermochemical and solvation parameters calculated at different temperatures.

Structure	M06-2X [a]	Thermal correction [b] (298.15 K) (313.15 K) (333.15 K)	Solvation energy [c] (298.15 K) (313.15 K) (333.15 K)	Energy barrier for N-chlorination [d]
**SMX^-**	-1175.056577	0.151796<br>0.148732<br>0.144492	-67.92<br>-67.78<br>-67.52	
$HOCl(H_2O)_3$	-765.264389	0.053542<br>0.051153<br>0.04788	-13.29<br>-13.25<br>-13.19	
**$TS_{ISX\text{-}Cl}$**	-1940.339865	0.224278	-61.86	71.1

		0.219943<br>0.213926	-61.73<br>-61.48	73.8<br>77.4
**TS$_{SO2N-Cl}$**	-1940.311856	0.217407<br>0.21263<br>0.206016	-66.2<br>-66.04<br>-65.78	108.4<br>110.1<br>112.2
**TS$_{PhN-Cl}$**	-1940.311363	0.221041<br>0.216704<br>0.21069	-74.15<br>-73.98<br>-73.67	85.9<br>88.9<br>92.7

[a] Single point energy calculated at M06-2X(D3)/6-311+G(d,2p)//B3LYP/6-31+G(d,p), in Hartree. [b] Thermal correction to Gibbs free energy calculated at B3LYP/6-31+G(d,p) level, at different temperatures, in Hartree. [c] Solvation energy calculated at the SMD(water)-B3LYP/6-31+G(d,p) level. Dielectric constant for water at different temperatures were used according to (Fernández et al., J. Chem. Phys. 2007, 126, 014507), as follows: ε =78.4 (298.15 K), ε =70.7 (313.15 K), and ε =60.2 (333.15 K). Solvation energy in kcal/mol. [d] Energy barrier for N-chlorination calculated as: M06-2X(D3)/6-311+G(d,2p) + B3LYP/6-31+G(d,p) thermal correction + B3LYP/6-31+G(d,p) solvation energy, in kJ/mol.

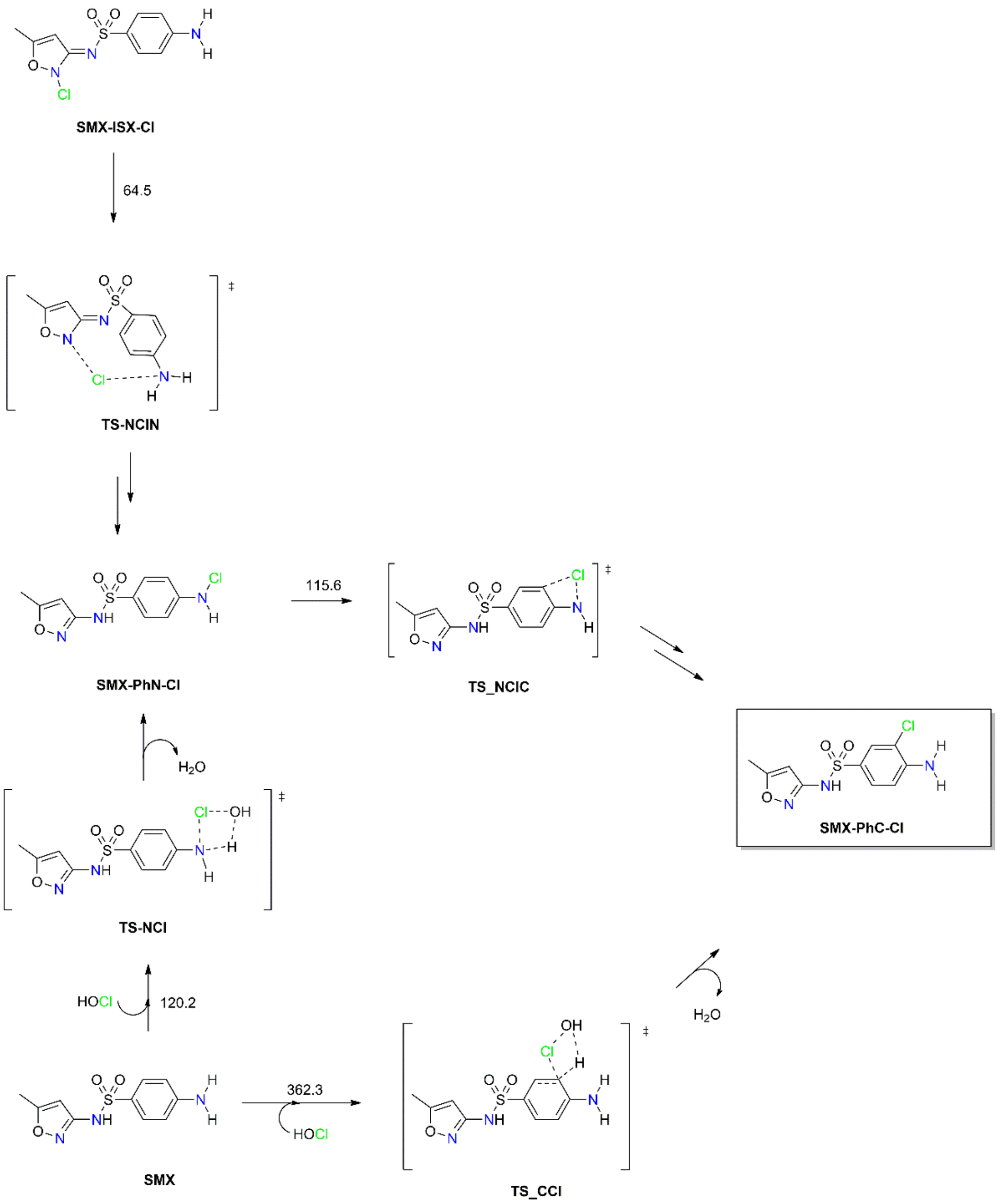

Scheme S1. Different reaction pathways resulting in the formation of **SMX-PhC-Cl**. All energy barriers ($\Delta G^{\ddagger}$, in kJ/mol) were calculated at the SMD(water)-B3LYP/6-31+G(d,p) level of theory, relative to corresponding reactant.

Coordinates, Gibbs free energies, thermal corrections and solvation energies calculated at the M06-2X(D3)/6-311+G(d,2p)// B3LYP/6-31+G(d,p) level

SDZ⁻
M062X/6-311+G(d,2p) EmpiricalDispersion=GD3
EE (a.u.)= -1154.03178795
B3LYP/6-31+G(d,p)
Thermal corr. to G (a.u.)= 0.144123
Gsolv(kcal/mol)= -69.83
S,0,2.7361799581,-1.7522378157,-0.1491249006
O,0,2.6406059304,-2.9351981377,-1.0366193615
O,0,3.3935623748,-1.9531658744,1.1664030623
N,0,3.3787927825,-0.5648423456,-1.0563382575
N,0,-3.0281172957,-0.3673704228,1.0132050698
C,0,1.0057953208,-1.3250846816,0.2145812336
C,0,0.0210731745,-1.5802448604,-0.7422934925
C,0,0.6497385994,-0.7610570418,1.440405092
C,0,-1.6836514698,-0.7160018438,0.7572289559
C,0,-1.3141451069,-1.2751231317,-0.4765958285
C,0,-0.686216836,-0.4633514055,1.7120469131
H,0,0.3076170643,-2.0342838756,-1.6852845751
H,0,1.4266624772,-0.5649282837,2.1695822849
H,0,-2.0768350159,-1.4753479054,-1.2270999709
H,0,-0.9592512744,-0.0248259401,2.6704115485
H,0,-3.2666271792,-0.3474985865,1.9967977111
H,0,-3.7010829329,-0.9142925408,0.4909306427
C,0,3.5727425176,0.6765805617,-0.5738461681
C,0,4.4669612744,2.7592369963,-1.0009932535
C,0,3.3559413131,2.324788046,1.0303882579
C,0,4.0506173356,3.2469214154,0.245700078
H,0,5.0071706457,3.4094422588,-1.6935466655
H,0,2.9731426901,2.6096707875,2.013416993
H,0,4.2451629069,4.2636238051,0.5708558778
N,0,3.1101037584,1.074902232,0.6553148753
N,0,4.2479329874,1.5242345903,-1.4245041221

SDZ⁻-PhN-Cl
M062X/6-311+G(d,2p) EmpiricalDispersion=GD3
EE (a.u.)= -1613.58647650
B3LYP/6-31+G(d,p)
Thermal corr. to G (a.u.)= 0.131099
Gsolv(kcal/mol)= -63.21
S,0,2.7302339989,-1.7436286504,-0.1606778711
O,0,2.6207893346,-2.9275641902,-1.0422841492
O,0,3.3850619753,-1.9418544524,1.1549227235
N,0,3.3478380398,-0.5528948246,-1.0667831206
N,0,-3.0492337081,-0.5262896212,1.0703845076
C,0,0.9945515145,-1.3164870869,0.2133541183
C,0,0.0083337907,-1.5645788862,-0.7502061601
C,0,0.6556870169,-0.7593941578,1.4472918852
C,0,-1.6743693596,-0.7127512927,0.765829205
C,0,-1.3229857496,-1.2681551088,-0.4755311505
C,0,-0.6760505037,-0.442845866,1.7155575216
H,0,0.2977451137,-2.0156330299,-1.6933442423
H,0,1.4409178479,-0.5710891158,2.1691777465
H,0,-2.1036847741,-1.475667448,-1.2011310094
H,0,-0.9475972507,0.0096153106,2.6677300125
H,0,-3.132811116,-0.2898745966,2.0591400815
Cl,0,-3.7406264322,1.0327355144,0.3150483771
C,0,3.5597299425,0.6869261086,-0.5759201881
C,0,4.4812598458,2.757079129,-0.9896612111
C,0,3.3583478937,2.3265003466,1.0361961796
C,0,4.0683434002,3.2417702396,0.2585761199
H,0,5.0309739449,3.4033823092,-1.6775348468
H,0,2.9754617763,2.610948399,2.0187731624
H,0,4.275150717,4.25387191,0.5898293748
N,0,3.0962932053,1.0817460941,0.6513672588
N,0,4.2465595959,1.5263178663,-1.4197983755

SDZ⁻-TS$_{PhN-Cl}$
M062X/6-311+G(d,2p) EmpiricalDispersion=GD3
EE (a.u.)= -1919.28534292
B3LYP/6-31+G(d,p)
Thermal corr. to G (a.u.)= 0.213349
Gsolv(kcal/mol)= -76.98
S,0,2.5858533987,-2.067878145,-0.1146879054
O,0,2.3452466826,-3.1924473424,-1.0451979513
O,0,3.2132389387,-2.3914136467,1.1888262063
N,0,3.3143763475,-0.906649989,-0.9672310619
N,0,-3.0114619635,-0.2513983165,1.2297132554
C,0,0.9022736045,-1.4828687501,0.2947079549
C,0,-0.1103245739,-1.5964246931,-0.6629894594
C,0,0.6333466491,-0.9424381829,1.5554524002
C,0,-1.6719781284,-0.6525107086,0.9052310319
C,0,-1.4058975185,-1.1760101101,-0.3651261553
C,0,-0.658930062,-0.5237098841,1.8628349936
H,0,0.1240009652,-2.03596594,-1.6265130093
H,0,1.4413767364,-0.8622403989,2.2718946546
H,0,-2.2084449202,-1.2703469553,-1.0902111874
H,0,-0.881504009,-0.097252271,2.8387434986
H,0,-3.1845119178,-0.3194163603,2.2311721613
O,0,-6.5253249681,0.3393665077,-1.1455482607
H,0,-6.5855944265,0.2358160789,-2.1027525272
H,0,-3.7897061471,-0.7713925356,0.6798666778
O,0,-4.9341724794,-1.3052307178,-0.1833134178
H,0,-5.4496927329,-2.076120943,0.0783445817
H,0,-5.6130148583,-0.585202605,-0.5945154371
H,0,-6.4605894002,1.508854294,-0.9261771199
O,0,-6.3991419989,2.7246650474,-0.7416722117
H,0,-7.2190780792,3.033270132,-0.3393501894
H,0,-5.2683161886,3.2535435488,-0.1315666344
O,0,-4.3626578576,3.6145962792,0.3363966933
H,0,-3.7961204909,3.9526625189,-0.3707612597
Cl,0,-3.5108977179,1.5801770469,0.8492784757
C,0,3.6509603745,0.2833057337,-0.4197347814
C,0,4.7856869441,2.2609285755,-0.7355670971
C,0,3.6008624336,1.8628215902,1.2616170021
C,0,4.410710189,2.7308569923,0.5299098626
H,0,5.4068216976,2.8753612552,-1.3905247218
H,0,3.2378688701,2.1430064361,2.2525895666
H,0,4.7170586529,3.7007017081,0.9068213125
N,0,3.2156115894,0.6692687703,0.819320553
N,0,4.4294626549,1.0803747704,-1.2207280838

SDZ⁻-TS$_{PhN-Cl-2w}$
G(a.u.)= -1842.822446
Gsolv(kcal/mol)= -75.27
S,0,2.537300165,-1.936623788,-0.2339498419
O,0,2.3424627907,-3.0593631072,-1.1773022693
O,0,3.1890189114,-2.2473735266,1.0608680717
N,0,3.2056828055,-0.7326133901,-1.0759283281
N,0,-3.1314251541,-0.4596610911,1.1983209303
C,0,0.8311766736,-1.4384293953,0.195870015
C,0,-0.1873204992,-1.6063895785,-0.7483544927
C,0,0.5502798819,-0.9150748913,1.4614875188
C,0,-1.7792445832,-0.762343193,0.8471188735
C,0,-1.5003847746,-1.2642405904,-0.4302119069
C,0,-0.7590325487,-0.5726234323,1.7882834045
H,0,0.0584559707,-2.0286384182,-1.7167008916
H,0,1.3626191541,-0.7902024791,2.1664972931
H,0,-2.3067265415,-1.3992580049,-1.1447577762
H,0,-0.9919593373,-0.1639256437,2.7695062536
H,0,-3.2771582305,-0.4983221338,2.2051397237
Cl,0,-3.889317865,1.2722589261,0.7065744121

O,0,-6.4927857023,0.6024699677,-0.8422063044
H,0,-6.730043104,0.5821654176,-1.7771234284
H,0,-3.9070560078,-1.0137125782,0.6709027339
O,0,-5.1347584227,-1.3615863097,-0.2001770631
H,0,-5.718274108,-2.1044146446,-0.0109745797
H,0,-5.7549155471,-0.495874666,-0.5039942798
H,0,-5.9543639004,1.7696401993,-0.5449805205
O,0,-5.4087823625,2.7147443197,-0.2320403691
H,0,-5.9050019122,3.1078331556,0.4991575696
C,0,3.499626893,0.4621161979,-0.5143869124
C,0,4.552493776,2.4872299123,-0.8122522167
C,0,3.4027903576,2.0151496252,1.1892461188
C,0,4.1709610251,2.9245174547,0.4629146317
H,0,5.1425603443,3.1347700124,-1.4640180931
H,0,3.038146025,2.2672834334,2.1871667491
H,0,4.4415463379,3.9008083093,0.8504283062
N,0,3.0607210065,0.8134948254,0.7336453316
N,0,4.2389449228,1.3001447155,-1.3105888833

SDZ⁻-TS$_{SO2N-Cl-2w}$
M062X/6-311+G(d,2p) EmpiricalDispersion=GD3
EE (a.u.)= -1842.78264280
B3LYP/6-31+G(d,p)
Thermal corr. to G (a.u.)= 0.194831
Gsolv(kcal/mol)= -79.08
O,0,-0.6379760091,0.8406612078,2.2937924412
N,0,-4.5554383652,-1.6288170334,-2.3948796398
C,0,-1.4073352669,-0.5406151581,0.1581915385
C,0,-1.3433162336,-1.7514483532,-0.5395820192
C,0,-2.5012811205,0.3139325002,0.0076182811
C,0,-3.4908440951,-1.2511087654,-1.5648955451
C,0,-2.3838548636,-2.0998935113,-1.3950403049
C,0,-3.5406092172,-0.0424806379,-0.8471015099
H,0,-0.4934352179,-2.4180578008,-0.4318090586
H,0,-2.528880561,1.2462669245,0.5578979013
H,0,-2.3290942986,-3.0397427849,-1.9386947069
H,0,-4.3996553209,0.6156319717,-0.9582423419
H,0,-4.3188282951,-2.3050352955,-3.1088337574
H,0,-5.1033638901,-0.8641518166,-2.7659227037
S,0,-0.1003385006,-0.1206376474,1.310513014
O,0,0.4561937874,-1.39278421,1.7860556984
N,0,1.2179591159,0.6814996378,0.5625197239
Cl,0,1.6374282662,-0.273361532,-1.4060019172
H,0,3.3571073384,-0.2182614252,0.7235453443
O,0,3.7277820887,-0.9157482054,0.1596518253
O,0,2.6825533538,-3.2200598738,0.9280186986
H,0,1.9421322852,-2.8680516523,1.444153966
H,0,3.1484457993,-2.3717397486,0.6313522312
O,0,1.3239657911,-3.8352206724,-1.3982595413
H,0,1.8394477559,-3.7597580037,-0.5555271156
H,0,1.5232043944,-2.9925866018,-1.8296079812
C,0,1.0714139348,2.0192350374,0.2089585687
C,0,-0.166760975,3.7975102856,-0.5107099411
C,0,2.1572001744,3.9994164418,-0.1119758904
C,0,0.9644351627,4.6137193096,-0.5064221659
H,0,-1.1404866779,4.1874314579,-0.8062562979
H,0,3.0924198197,4.5571650092,-0.0792309024
H,0,0.9220122238,5.6586240248,-0.794711903
N,0,-0.1270771599,2.5105865218,-0.1705238993
N,0,2.2248581965,2.7217067797,0.2490536402

SDZ-enol
M062X/6-311+G(d,2p) EmpiricalDispersion=GD3
EE (a.u.)= -1154.54089978
B3LYP/6-31+G(d,p)
Thermal corr. to G (a.u.)= 0.155634
Gsolv(kcal/mol)= -21.32
S,0,0.204269386,-0.0259589875,1.5480262874
O,0,-0.5370446259,1.4083928461,1.8226582486
O,0,0.6121466026,-0.6241759581,2.8199789617
N,0,-0.9058320155,-0.6682980694,0.6350570897
N,0,5.0839810665,1.7394524208,-1.3805749855
C,0,1.6569431344,0.5320917447,0.6835484841
C,0,1.5292395193,1.1697660012,-0.5553001758
C,0,2.9157126428,0.2946741004,1.2404677393
C,0,3.949295713,1.3653396555,-0.6832775892
C,0,2.6674030392,1.5854684172,-1.2314994076
C,0,4.0537868682,0.7124613974,0.561311899
H,0,-1.4738762272,1.2657902598,1.5881033333
H,0,0.547729374,1.3386633442,-0.9858796691
H,0,2.9932549841,-0.2125537167,2.1954093135
H,0,2.5717144091,2.079922263,-2.1942702274
H,0,5.0344446336,0.5275499614,0.9911481046
H,0,5.9497971315,1.810566292,-0.8668645273
H,0,4.9811779238,2.4229993459,-2.1159089291
C,0,-0.6964123079,-1.9175396615,0.0771174343
C,0,0.6407930524,-3.7237238338,-0.3479757813
C,0,-1.577969658,-3.6171844439,-1.1545921291
C,0,-0.3859020488,-4.3394691345,-1.0608287765
H,0,1.6105313789,-4.2025825727,-0.2236867506
H,0,-2.4329359235,-4.0168760211,-1.6977080939
H,0,-0.2667019309,-5.315990966,-1.5157245078
N,0,0.5025775336,-2.5236069473,0.2202782865
N,0,-1.7503306555,-2.4172497368,-0.5997026317

SDZiso-TS'$_{SO2N-Cl-2w}$
M062X/6-311+G(d,2p) EmpiricalDispersion=GD3
EE (a.u.)= -1843.37665972
B3LYP/6-31+G(d,p)
Thermal corr. to G (a.u.)= 0.205589
Gsolv(kcal/mol)= -30.85
S,0,0.8600444121,0.7317086106,1.8439426503
O,0,0.4492477115,2.0690900224,2.2689983748
O,0,1.2185917936,-0.2952969566,2.8262072453
N,0,-0.5129413845,0.1461249802,0.9795482074
N,0,5.3221058866,1.3994947523,-2.0659889292
C,0,2.1897071419,0.9150987631,0.669892723
C,0,2.2785089383,2.0927597614,-0.081247613
C,0,3.1492340445,-0.092228695,0.5303794188
C,0,4.3011603258,1.2525010591,-1.1373030818
C,0,3.3242278309,2.2562407825,-0.9818310945
C,0,4.1984061099,0.0780519568,-0.3651977547
H,0,-4.1438321647,-0.0064527328,1.3307333369
H,0,1.5420805164,2.8768924993,0.0553467522
H,0,3.0694959516,-0.9919438248,1.1284075114
H,0,3.3947713032,3.1707518021,-1.5647991811
H,0,4.949334698,-0.7008021634,-0.4700921663
H,0,6.1430246632,0.8247976733,-1.941196414
H,0,5.5302119932,2.3381199433,-2.3749869433
C,0,-0.626668474,-1.1492888325,0.577384558
C,0,0.2666530696,-3.215183377,0.1387038162
C,0,-2.039205891,-2.8465291702,-0.1827547607
C,0,-0.9766076598,-3.730200081,-0.251778189
H,0,1.1568134808,-3.8417114336,0.1210669132
H,0,-3.0559765684,-3.1270936652,-0.4434387173
H,0,-1.1022803754,-4.7534464461,-0.5822400462
N,0,0.4506351776,-1.9592923589,0.5271674023
N,0,-1.878373251,-1.5814849235,0.2344628367
O,0,-4.0935824816,-0.4770339681,0.4869759657
H,0,-4.3534989682,0.259257208,-0.3406393475
H,0,-2.9733451147,-0.9194220109,0.3713204195
O,0,-4.5642394282,1.0868225451,-1.3088559309
H,0,-5.4363347215,1.4989539659,-1.312556591
H,0,-3.7516134637,1.8724063724,-1.3382494861
Cl,0,-1.5331999878,1.4536881878,-0.0831823102
O,0,-2.739217351,2.6827052979,-1.2378535753
H,0,-2.2599427933,2.7722264663,-2.0742989896

SDZiso
M062X/6-311+G(d,2p) EmpiricalDispersion=GD3

EE (a.u.)= -1154.55316979
B3LYP/6-31+G(d,p)
Thermal corr. to G (a.u.)= 0.158105
Gsolv(kcal/mol)= -29.91
S,0,-0.1017526087,1.7494290624,0.47273737
O,0,0.2960245371,3.033820027,-0.1150845112
O,0,-0.4778039822,1.6768559666,1.8931472685
N,0,-1.3859880334,1.2419505879,-0.5013480422
N,0,4.4021095222,-2.0655963549,-0.5259211583
C,0,1.2365139286,0.5946464279,0.177163589
C,0,2.0527970615,0.7656289286,-0.9457918499
C,0,1.4833782162,-0.4441654697,1.0784250686
C,0,3.3715339882,-1.1636486676,-0.2760229123
C,0,3.1083119773,-0.1106850106,-1.1739147496
C,0,2.5453807643,-1.3147431176,0.8540096354
H,0,1.869222433,1.5939385703,-1.6218437419
H,0,0.8500580191,-0.5534179237,1.9508782185
H,0,3.7433678192,0.0240794238,-2.0458596074
H,0,2.7422306909,-2.118444268,1.5592461155
H,0,4.7493733336,-2.5836316105,0.2689418667
H,0,5.1378905393,-1.7490982151,-1.1417347209
C,0,-2.0318503059,0.1400011036,-0.2669594385
C,0,-2.5265992592,-1.88587727,0.7387382525
C,0,-3.9580693366,-1.1451981914,-0.9965230552
C,0,-3.6789169678,-2.1096404684,-0.0664561151
H,0,-2.2477267132,-2.6286620194,1.4860596171
H,0,-4.797137043,-1.1849515703,-1.6824401315
H,0,-4.2951563408,-2.9917065532,0.0436826232
N,0,-1.7420400365,-0.8378992768,0.6572268273
N,0,-3.1533322207,-0.062627699,-1.0890788899
H,0,-3.3358373426,0.6779439081,-1.7571740286

SDZ
M062X/6-311+G(d,2p) EmpiricalDispersion=GD3
EE (a.u.)= -1154.57632500
B3LYP/6-31+G(d,p)
Thermal corr. to G (a.u.)= 0.157728
Gsolv(kcal/mol)= -19.24
S,0,2.4826424587,-1.8010072174,-0.1851512169
O,0,2.4737906788,-2.8414805924,-1.2214612836
O,0,3.2465006725,-1.9589435253,1.0512283922
N,0,3.0858409844,-0.4585653845,-1.0662581864
N,0,-3.157361924,-0.4433197864,1.180471241
C,0,0.8046516874,-1.3808867588,0.223729645
C,0,-0.2088400071,-1.6217927352,-0.7104685783
C,0,0.5052197629,-0.8480151412,1.4810242048
C,0,-1.8494417298,-0.7818399302,0.8745911441
C,0,-1.5257621836,-1.3213075356,-0.3876150522
C,0,-0.8135768705,-0.552406273,1.8026239833
H,0,3.4944012618,-0.7377264669,-1.9524882813
H,0,0.0348640481,-2.0564861173,-1.6738393115
H,0,1.3032710474,-0.6720091204,2.1921379397
H,0,-2.314657308,-1.509525906,-1.110826832
H,0,-1.0495307785,-0.1390841937,2.7795989108
H,0,-3.3931688669,-0.3300706666,2.1554823411
H,0,-3.8894583485,-0.8652873287,0.628206364
C,0,3.5326335304,0.7479768427,-0.550250793
C,0,3.5363668355,2.3044565169,1.096185012
C,0,4.7688090403,2.6198977194,-0.8987156558
C,0,4.3923569625,3.1141358018,0.3513498125
H,0,3.1863349037,2.6100357511,2.0801289383
H,0,5.4297786993,3.1834610699,-1.5539226
H,0,4.7434733166,4.0708142056,0.7197219168
N,0,3.091137462,1.1232701204,0.6570613245
N,0,4.3531556645,1.438872652,-1.3611723792

SDZ-PhN-Cl
M062X/6-311+G(d,2p) EmpiricalDispersion=GD3
EE (a.u.)= -1614.12524833
B3LYP/6-31+G(d,p)
Thermal corr. to G (a.u.)= 0.145196
Gsolv(kcal/mol)= -17.62
S,0,1.0793379825,-1.8231637117,0.1427027474
O,0,0.9202187237,-3.0086041843,-0.7077304169
O,0,1.678487893,-1.9058814284,1.4724208524
N,0,2.0217805232,-0.8059652468,-0.8605193117
N,0,-4.2951815728,0.7337332477,0.6244471547
C,0,-0.500282061,-1.0023379472,0.2771044719
C,0,-1.4586148717,-1.2069861297,-0.7191979771
C,0,-0.7701106525,-0.1899983194,1.380389057
C,0,-2.9833296732,0.2352805432,0.4971806445
C,0,-2.693899161,-0.5774283381,-0.6150472362
C,0,-2.0128842849,0.4259075793,1.4927939519
H,0,2.433582798,-1.3149533833,-1.636583065
H,0,-1.2381633394,-1.8508087836,-1.5635341174
H,0,-0.0108919103,-0.0475774468,2.1396403484
H,0,-3.4423504478,-0.7298407962,-1.3880594672
H,0,-2.2319103404,1.0534423614,2.3486478828
C,0,2.6865528017,0.353368173,-0.4821067792
C,0,2.897270873,2.140100455,0.8939324855
C,0,4.3424098374,1.8309179142,-0.9544666976
C,0,3.978519592,2.6060122994,0.1478630257
H,0,4.5044753872,3.5172605001,0.4069740456
N,0,2.2350092058,1.0199819973,0.5868680371
N,0,3.7108901787,0.7001995687,-1.2790924892
H,0,5.1698979199,2.117991917,-1.5997073046
H,0,2.539555942,2.681739922,1.767066493
H,0,-4.7789001802,0.7818882316,-0.2679315606
Cl,0,-4.469052703,2.3333008955,1.3493567248

SDZ-TS'$_{PhN\text{-}Cl\text{-}2w}$
M062X/6-311+G(d,2p) EmpiricalDispersion=GD3
EE (a.u.)= -1843.36861627
B3LYP/6-31+G(d,p)
Thermal corr. to G (a.u.)= 0.205133
Gsolv(kcal/mol)= -29.08
S,0,2.2565448101,-2.0599961581,0.3586911149
O,0,2.1764197194,-3.2536959047,-0.4894592197
O,0,2.9422607103,-2.0761774798,1.6480546557
N,0,2.9913679678,-0.9323119043,-0.6881877324
N,0,-3.3132030637,-0.0507795913,1.2018397005
C,0,0.5971103686,-1.4280542246,0.6090656411
C,0,-0.3882827871,-1.7197349822,-0.3387624672
C,0,0.3127354277,-0.6912111337,1.759699404
C,0,-1.9815614487,-0.5135074904,1.0147775506
C,0,-1.6850550442,-1.2564932563,-0.1374049952
C,0,-0.9857435426,-0.2286694982,1.9583944334
H,0,3.3930255161,-1.3744656716,-1.5092598018
H,0,-0.1439334912,-2.3159811003,-1.2108819506
H,0,1.0988875833,-0.485044172,2.4754796114
H,0,-2.4732509738,-1.474052626,-0.8506893907
H,0,-1.2241049218,0.3544316031,2.8438049258
H,0,-3.516381418,0.1742394377,2.1737792492
Cl,0,-3.8162084848,1.574045623,0.1819850845
O,0,-6.121176131,0.7025696056,-1.5362805383
H,0,-6.2596280153,0.449620373,-2.4568687581
H,0,-4.1534002034,-0.6518020357,0.6698897508
O,0,-5.1202557917,-1.0828713834,-0.2135383777
H,0,-5.7831003036,-1.7068435425,0.1049750121
H,0,-5.6277623512,-0.2329386383,-0.8715094752
H,0,-5.543147321,1.80852428,-1.4488148256
O,0,-4.8929360174,2.7813211531,-1.2559832723
H,0,-5.417085684,3.4260015132,-0.7580138234
C,0,3.5225410749,0.3062431127,-0.3454690005
C,0,3.5963592675,2.1009861399,1.0326665846
C,0,4.9229838647,1.9933944733,-0.9244659228
C,0,4.5436006962,2.7099397818,0.2116958352
H,0,3.2375874333,2.5879724263,1.9368708976
H,0,5.6517117861,2.3907967928,-1.6274802013
H,0,4.9592174337,3.6841351276,0.4402978159

N,0,3.0692002042,0.9016952946,0.7632200811
N,0,4.4261063206,0.7880109956,-1.2133619156

SDZ-PYR-Cl
M062X/6-311+G(d,2p) EmpiricalDispersion=GD3
EE (a.u.)= -1614.09523581
B3LYP/6-31+G(d,p)
Thermal corr. to G (a.u.)= 0.144799
Gsolv(kcal/mol)= -25.62
S,0,-0.145490048,1.7721793219,0.3461159854
O,0,0.234556,3.0286449348,-0.3066045252
O,0,-0.5454080067,1.768023164,1.7609297418
N,0,-1.4098401175,1.1854715219,-0.6198936479
N,0,4.4254654765,-2.015855772,-0.3940281086
C,0,1.2111487542,0.6248013431,0.1277727109
C,0,2.0189973047,0.7286773378,-1.0096950654
C,0,1.4823124779,-0.3390179295,1.1024416313
C,0,3.3786337602,-1.1194564096,-0.2058830208
C,0,3.090260193,-0.1413632684,-1.178220494
C,0,2.5607645446,-1.2023625288,0.9374592783
H,0,1.8160424113,1.5000363591,-1.7449900874
H,0,0.855993787,-0.3941936148,1.9850704043
H,0,3.7183563871,-0.0596203772,-2.0616008159
H,0,2.7773625164,-1.9467582395,1.6996060899
H,0,4.785860394,-2.470923598,0.4327377837
H,0,5.1499830371,-1.7364465863,-1.0402013396
C,0,-2.0264951038,0.0888611406,-0.3370216481
C,0,-3.9059687008,-1.3503255965,-0.9683171082
C,0,-2.4583574734,-1.8914519081,0.8241670137
C,0,-3.5935993434,-2.2221168684,0.038684183
H,0,-4.7362716807,-1.4811222883,-1.6509340918
H,0,-2.1457928115,-2.5552588708,1.6295828025
H,0,-4.1829448421,-3.1134985991,0.2090565309
N,0,-1.7262810647,-0.8207079474,0.6502999337
N,0,-3.1479004795,-0.2386342624,-1.1550465781
Cl,0,-3.5833980818,0.834883862,-2.4381384283

SDZ-PhCCl
M062X/6-311+G(d,2p) EmpiricalDispersion=GD3
EE (a.u.)= -1614.17729036
B3LYP/6-31+G(d,p)
Thermal corr. to G (a.u.)= 0.146208
Gsolv(kcal/mol)= -17.41
S,0,-0.0803605579,1.6833444426,0.3802283649
O,0,0.225538349,2.9656290425,-0.2651129771
O,0,-0.5010581065,1.6065665454,1.7773592061
N,0,-1.3224988582,1.0805058305,-0.6320371745
N,0,4.5487325486,-1.9861768848,-0.4429147831
C,0,1.2962718178,0.581448301,0.1352827951
C,0,2.1805456749,0.8088537522,-0.9246184217
C,0,1.4938063267,-0.4798968941,1.019897602
C,0,3.495491989,-1.1276927071,-0.2336288328
C,0,3.2634667924,-0.0392181171,-1.1026040351
C,0,2.5828222081,-1.3178309047,0.8284366871
H,0,-1.7040018462,1.7998828567,-1.2382284717
H,0,2.0252814003,1.6512312654,-1.5893796867
H,0,0.8057145162,-0.6438352225,1.8394224761
H,0,3.9548500199,0.132923229,-1.9227491558
H,0,4.8132282283,-2.6084198335,0.3064628952
H,0,5.2995332812,-1.6780231767,-1.0412959295
C,0,-2.1576176825,0.0020177085,-0.3748128498
C,0,-2.566492894,-1.962012261,0.6780444429
C,0,-4.1127537782,-1.0425095423,-0.8595977543
C,0,-3.7919611049,-2.0747892584,0.0235132381
H,0,-2.230022263,-2.7214963417,1.3806278401
H,0,-5.0452610272,-1.0467791936,-1.4199444588
H,0,-4.4557098419,-2.9151581547,0.1889327917
N,0,-1.734452721,-0.9337677739,0.4836424839
N,0,-3.3100965204,0.0043156543,-1.0656811765
Cl,0,2.8433379995,-2.6531916122,1.9476945342

SDZ-Cl$_{Int}$
G(a.u.)= -1614.111066
Gsolv(kcal/mol)= -19.88
S,0,0.5657225529,1.8542709888,-0.5137460352
O,0,0.4382834016,3.2294195925,-0.0191716711
O,0,0.8891051362,1.5663146227,-1.9072704471
N,0,1.7394630193,1.201449493,0.5330606979
N,0,-4.7329963963,-0.3687575862,0.5858835827
C,0,-0.9292361867,0.9653849356,-0.0691832883
C,0,-1.5727663333,1.3079583839,1.1948536719
C,0,-1.414694497,0.0383056729,-0.9122788974
C,0,-3.4796629405,-0.098869658,0.5672450826
C,0,-2.7721340386,0.7751996811,1.5113843272
C,0,-2.6593054362,-0.7095322412,-0.5587670319
H,0,2.247799712,1.9230311102,1.0358952607
H,0,-1.0814793689,2.0168900084,1.8524422781
H,0,-0.8933363428,-0.213458567,-1.8287200983
H,0,-3.2760228265,1.0450745184,2.4354923834
C,0,2.4191070338,-0.0052075434,0.3613944953
C,0,2.5082203404,-2.101322441,-0.4852623586
C,0,4.2372301106,-1.2490649045,0.8904420921
C,0,3.7374399828,-2.3124791792,0.137874505
H,0,4.2733847867,-3.2496430507,0.0448857739
N,0,1.8336906911,-0.9518961691,-0.3768260944
N,0,3.590862955,-0.0851920278,1.0080858541
H,0,5.187047091,-1.3232276262,1.4152026666
H,0,2.0382728109,-2.8752274851,-1.0878110612
H,0,-3.3027677208,-0.8851867332,-1.4204057899
Cl,0,-2.1270854643,-2.4128520387,-0.0452081632
H,0,-5.1888303624,0.066903894,1.3936331052

SDZ$^-$-TS_Cl$_1$
M062X/6-311+G(d,2p) EmpiricalDispersion=GD3
EE (a.u.)= -1634.574994
B3LYP/6-31+G(d,p)
Thermal corr. to G (a.u.)= 0.137516
O,0,-0.5268985429,2.8242051428,-0.7183307883
O,0,-1.3037274914,1.782221579,1.4536827706
N,0,-1.1566201326,0.3544546626,-0.7857433184
N,0,4.8063310918,-0.414843373,0.8594143172
C,0,0.95503364,0.9544567827,0.3864759799
C,0,1.8117590459,0.7880151217,-0.7536682816
C,0,1.3926569847,0.6812631868,1.6890972322
C,0,3.601955451,0.0574226695,0.7606764049
C,0,3.0935105946,0.3776645639,-0.5739104263
C,0,2.6970481228,0.2734409296,1.902838863
H,0,1.4075624509,0.9976900738,-1.7368762667
H,0,0.7151652375,0.8629239174,2.5159804562
H,0,3.107948473,0.220233431,2.9016163409
H,0,4.9604096652,-0.7440701314,1.823713485
S,0,-0.6988599348,1.6176941997,0.119063598
Cl,0,3.5039376656,-2.0874910231,3.4320692574
H,0,3.7725462847,0.2199209714,-1.4055675399
C,0,-2.3376116211,-0.3052851489,-0.6435042056
N,0,-3.3987806002,0.2768131596,-0.0216610632
N,0,-2.3692902926,-1.5399895343,-1.2239090496
C,0,-4.5231956595,-0.4297696001,0.0398964926
C,0,-3.5128406149,-2.2097851195,-1.1404258744
C,0,-4.6534536272,-1.7098635015,-0.5030086602
H,0,-5.3618025651,0.0538320274,0.541687248
H,0,-3.5221055337,-3.1960993599,-1.6054150971
H,0,-5.5773377117,-2.2746970673,-0.4418921348

SDZ-TS-NClN
M062X/6-311+G(d,2p) EmpiricalDispersion=GD3
EE (a.u.)= -1614.02419251
B3LYP/6-31+G(d,p)
Thermal corr. to G (a.u.)= 0.143621
Gsolv(kcal/mol)= -36.63

N,0,-1.2090320595,0.9377232043,-1.0542767234
O,0,-1.0351509603,2.3292428077,1.2247199559
S,0,-0.4714775201,2.0723895903,-0.1020763775
O,0,-0.1460884179,3.1993619556,-0.9808998259
C,0,0.9644498656,1.0377796854,0.0273175614
C,0,1.1582636246,0.2268331138,1.1561292682
C,0,1.8541466733,0.9795388734,-1.0968079675
C,0,2.2477789055,-0.6154615732,1.2001323898
H,0,0.4450456929,0.2684400646,1.9702230946
C,0,2.9566141258,0.1809770616,-1.0555253625
H,0,1.6364296479,1.6003308076,-1.9586162454
C,0,3.2242294172,-0.6092113881,0.1199171302
H,0,2.4645537805,-1.2010551876,2.0861785446
H,0,3.6596849636,0.1488972871,-1.8816170119
N,0,4.4549097045,-1.0986019477,0.3255831891
H,0,5.0891774857,-1.1801761754,-0.4577702445
H,0,4.603964068,-1.7591659403,1.0771205869
Cl,0,1.8860879453,-2.77638687,-0.2690538942
C,0,-1.9776008121,-0.0904227925,-0.5781128681
C,0,-2.8195541578,-1.4067978315,1.0912753622
C,0,-3.4252541223,-1.7551858689,-1.1784314578
C,0,-3.5734183576,-2.1300230219,0.161837651
H,0,-2.8486977924,-1.6530059088,2.1511668336
H,0,-3.9561014764,-2.2804476369,-1.970131975
H,0,-4.2183188108,-2.9485950068,0.4607267497
N,0,-2.6319600954,-0.7612694605,-1.5628314847
N,0,-2.0283163481,-0.3970542612,0.7425912912

SIZ$^-$
M062X/6-311+G(d,2p) EmpiricalDispersion=GD3
EE (a.u.)= -1214.37405811
B3LYP/6-31+G(d,p)
Thermal corr. to G (a.u.)= 0.176286
Gsolv(kcal/mol)= -64.17
S,0,-2.2020101922,-2.0805061886,0.1681260859
O,0,-1.9968437156,-3.145264927,1.1774639577
O,0,-2.8009015922,-2.4777750256,-1.1303011781
N,0,-2.9748912443,-0.8768268785,0.918539037
N,0,3.3674878407,-0.1016792313,-1.0817959516
C,0,-0.5260056712,-1.4944923548,-0.2279404666
C,0,0.5111949138,-1.7052969397,0.6834973498
C,0,-0.2665831397,-0.8313231518,-1.4297529335
C,0,-3.4045124954,0.2311523962,0.2873755945
C,0,2.073218103,-0.594934485,-0.8099800921
C,0,1.8015061037,-1.2590933412,0.3968585171
C,0,1.023137451,-0.3852031855,-1.7177484228
H,0,0.2983531745,-2.234912277,1.6062704554
H,0,-1.0759324276,-0.6674574953,-2.1318902952
H,0,2.6052884033,-1.4275559007,1.111486006
H,0,1.21712688,0.1348051706,-2.6539922957
H,0,3.5648547546,0.0148164686,-2.0677183087
H,0,4.1097375329,-0.6143694481,-0.6226828373
O,0,-3.0456109446,0.5693560267,-0.9883670234
N,0,-3.6364023686,1.8169707318,-1.3384254395
C,0,-4.3186946919,2.1731448657,-0.2621305189
C,0,-4.2251758693,1.2381992579,0.7945730873
C,0,-4.8493078032,1.2747271363,2.1549561133
H,0,-4.6328589536,0.3365590459,2.6748287186
H,0,-4.4636773473,2.0953442738,2.7789375514
H,0,-5.9423293657,1.3901700088,2.1127245155
C,0,-5.0849873938,3.4628221459,-0.2810171611
H,0,-6.1543786003,3.2903763294,-0.1061758376
H,0,-4.7327406219,4.1451712387,0.5027261434
H,0,-4.9659017192,3.954863734,-1.2506833707

SIZ$^-$-PhN-Cl
M062X/6-311+G(d,2p) EmpiricalDispersion=GD3
EE (a.u.)= -1634.61171036
B3LYP/6-31+G(d,p)
Thermal corr. to G (a.u.)= 0.138934
Gsolv(kcal/mol)= -60.64
S,0,-2.1981059926,-2.072374762,0.1724141025
O,0,-1.9882816514,-3.1369249395,1.1780214022
O,0,-2.7915280105,-2.4643873523,-1.1280020995
N,0,-2.9429411661,-0.856989894,0.9197114245
N,0,3.4047464339,-0.2496038754,-1.1257518551
C,0,-0.516419785,-1.4866494742,-0.2247931004
C,0,0.5233062544,-1.7176122148,0.6861993439
C,0,-0.2727558906,-0.8026031356,-1.4185600801
C,0,-3.397002052,0.2407707232,0.2814594543
C,0,2.0658588162,-0.5895905351,-0.7989750069
C,0,1.8113902109,-1.2791419902,0.3981327731
C,0,1.012235987,-0.3352598872,-1.6917795961
H,0,0.3064102253,-2.2659834988,1.59656113
H,0,-1.0891647986,-0.6268583274,-2.1091601113
H,0,2.6333866917,-1.4731446089,1.080680434
H,0,1.2034108313,0.2212889214,-2.6072874434
H,0,3.4273092787,0.0944451878,-2.0857766345
Cl,0,3.9919142155,1.2780410256,-0.2288528373
O,0,-3.0571026419,0.5661243387,-1.0020543916
N,0,-3.6638569818,1.8024523646,-1.3578939363
C,0,-4.3356573235,2.1659453161,-0.278367519
C,0,-4.2184864371,1.2432432155,0.7888604615
C,0,-4.8253733606,1.2913359891,2.1566347271
H,0,-4.5681674604,0.3760532347,2.6978498088
H,0,-4.4616065838,2.1424091572,2.7511630336
H,0,-5.9220341679,1.3655380924,2.1261320033
C,0,-5.1141159335,3.4477540884,-0.301223201
H,0,-6.1794607615,3.2664378098,-0.1124918015
H,0,-4.7590994543,4.1401900884,0.4720523127
H,0,-5.0106886618,3.9316300624,-1.2765460878

SIZ$^-$-TS$_{PhN\text{-}Cl}$
M062X/6-311+G(d,2p) EmpiricalDispersion=GD3
EE (a.u.)= -1979.62684946
B3LYP/6-31+G(d,p)
Thermal corr. to G (a.u.)= 0.245848
Gsolv(kcal/mol)= -71.20
S,0,2.3344348016,-1.7985644338,-0.8053612412
O,0,2.0085201248,-2.9490370598,-1.6747538954
O,0,3.1375975731,-2.0606008074,0.4117044631
N,0,2.8892659826,-0.625598892,-1.7518471208
N,0,-3.0507756432,0.0099507334,1.2333487774
C,0,0.7114932349,-1.2350069408,-0.1845511529
C,0,-0.4501480749,-1.6018948902,-0.8730296434
C,0,0.6360532154,-0.4387696834,0.9641462981
C,0,-1.7669772379,-0.3968046587,0.7414325159
C,0,-1.6977022855,-1.1835144642,-0.414374812
C,0,-0.6064751951,-0.0106680491,1.4233120424
H,0,-0.3595662547,-2.2327349668,-1.7505670633
H,0,1.546754412,-0.160907136,1.4812019052
H,0,-2.6105043979,-1.4753629361,-0.9246344102
H,0,-0.6754532553,0.6178514504,2.308486625
H,0,-3.0238607274,0.1892204681,2.2355593503
O,0,-6.9587611655,-0.2008712674,-0.5152094055
H,0,-7.1620013555,-0.549113308,-1.3915325048
H,0,-3.8646594281,-0.6655990494,0.9929148823
O,0,-5.0870755249,-1.4600380692,0.5210143736
H,0,-5.4815984926,-2.151756165,1.0635885766
H,0,-5.886842878,-0.9046308634,0.074036786
H,0,-6.9752464005,0.9889291779,-0.5996713602
O,0,-7.005391932,2.2118105375,-0.7266177255
H,0,-7.776384975,2.5647649701,-0.2678532715
H,0,-5.8497256199,2.938195495,-0.4664467557
O,0,-4.9227394215,3.4564148068,-0.2621690208
H,0,-4.5123085446,3.6488773315,-1.116456063
Cl,0,-3.7934666575,1.6654534765,0.5384595642
C,0,3.3993938683,0.5318822571,-1.2794511024
C,0,4.0975302708,1.5069452906,-1.9826529388
O,0,3.2439622503,0.9527883272,0.0116972219

C,0,4.3406879739,2.5176935652,-1.0201550331
N,0,3.8521221885,2.2277899552,0.1735380256
C,0,4.4907804132,1.4578157161,-3.4263720927
H,0,5.5765129071,1.5567808643,-3.5672542746
H,0,4.1853342028,0.4976897232,-3.8522858712
H,0,4.0150225522,2.2518834028,-4.0200679357
C,0,5.0673176851,3.8147465007,-1.2162527484
H,0,4.5768566322,4.4309340138,-1.9796912786
H,0,5.0922761816,4.3761074504,-0.2781660751
H,0,6.0985397373,3.642968237,-1.5482541508

SIZ$^-$-TS$_{PhN-Cl-2w}$
M062X/6-311+G(d,2p) EmpiricalDispersion=GD3
EE (a.u.)= -1903.18019464
B3LYP/6-31+G(d,p)
Thermal corr. to G (a.u.)= 0.225630
Gsolv(kcal/mol)= -69.40
S,0,2.4571540379,-1.9578981395,-0.276301266
O,0,2.2328068833,-3.1044720576,-1.1819650369
O,0,3.1044263397,-2.2277349546,1.028714949
N,0,3.1319781207,-0.7867711308,-1.1447861133
N,0,-3.1173311982,-0.1276157691,1.1100302286
C,0,0.7759589654,-1.3819932808,0.1422748924
C,0,-0.300133887,-1.7550273194,-0.6718183342
C,0,0.5711504387,-0.5701517613,1.2644354398
C,0,3.5935950857,0.3640909366,-0.6114858743
C,0,-1.7919641837,-0.5307351158,0.7677658345
C,0,-1.5907817458,-1.3311425066,-0.3642598457
C,0,-0.7141945896,-0.1345021457,1.5712020503
H,0,-0.1102032444,-2.3948878144,-1.5266095887
H,0,1.4167933715,-0.2869093137,1.879748119
H,0,-2.4395751875,-1.623358898,-0.9751668901
H,0,-0.8830400038,0.5040821441,2.4356538561
H,0,-3.1977253291,0.1145625733,2.0954586467
Cl,0,-3.9191965043,1.4197664775,0.205626667
O,0,-6.6088951953,0.37079143,-0.9376485821
H,0,-6.9075632682,0.0962935213,-1.8129587329
H,0,-3.9236265788,-0.7911333087,0.8016939699
O,0,-5.1990055508,-1.3554542098,0.1333316105
H,0,-5.7663705278,-2.0054785431,0.5619868862
H,0,-5.8423685712,-0.5995681406,-0.3570755781
H,0,-6.0617327252,1.5703732991,-1.0079478839
O,0,-5.5063620096,2.5595035023,-1.0033643391
H,0,-5.9574976223,3.1432454701,-0.3779115616
O,0,3.3035176123,0.7775014127,0.658806293
N,0,3.898900172,2.0469475359,0.8941839194
C,0,4.5148038964,2.3412817606,-0.2380178419
C,0,4.3697407998,1.3389267461,-1.2284282032
C,0,4.9140278176,1.2973977955,-2.6224159283
H,0,4.6473270656,0.3436969938,-3.0869694782
H,0,4.5107348853,2.1006634621,-3.2559325645
H,0,6.0094128199,1.3872448034,-2.6461293012
C,0,5.2660649936,3.6346555809,-0.3457194917
H,0,6.3257138169,3.4581665224,-0.5673968341
H,0,4.8632744196,4.2593701659,-1.1522791982
H,0,5.194364611,4.189312736,0.5939617157

SIZ$^-$-TS$_{SO2N-Cl-2w}$
M062X/6-311+G(d,2p) EmpiricalDispersion=GD3
EE (a.u.)= -1903.23668590
B3LYP/6-31+G(d,p)
Thermal corr. to G (a.u.)= 0.225206
Gsolv(kcal/mol)= -59.91
O,0,1.365522038,-3.4703235459,0.7442466341
N,0,5.2923090785,1.785095025,0.2433887632
C,0,2.15326744,-0.9642201615,0.7870312065
C,0,1.8843803641,0.4055715482,0.7150588591
C,0,3.4662544417,-1.4238972395,0.6883778115
C,0,-1.5603448478,-1.431539088,0.1805899176
C,0,4.2452907675,0.8625150661,0.4127914115
C,0,2.9184448504,1.31455973,0.5232034932
C,0,4.508838092,-0.513614361,0.509559943
H,0,0.8618097946,0.7624503223,0.7685501234
H,0,3.6585544128,-2.4905798329,0.7300155007
H,0,2.6825994666,2.369979017,0.4133897867
H,0,5.5321389112,-0.8740354227,0.4254039138
H,0,4.9998442115,2.6658773112,-0.1612983325
H,0,6.1053823969,1.4143947949,-0.2314053868
S,0,0.8017493748,-2.1316851108,1.0307483247
O,0,0.2393784467,-1.9139044538,2.3858127663
N,0,-0.2588554306,-1.6687238194,-0.1339143901
Cl,0,1.8095636136,-1.6319327571,-3.1532700151
H,0,0.1923441374,-1.3407833705,-1.6200805729
O,0,0.3159545932,-0.9917963989,-2.5954469726
O,0,-0.4770614553,1.6569788333,-1.9827010278
H,0,-1.1335409621,1.2570459471,-1.387935726
H,0,-0.0257051595,0.8755788136,-2.3600282661
O,0,1.0940527042,3.7136457915,-0.954020184
H,0,0.5802064964,2.9614438847,-1.3313471447
H,0,0.4863182519,4.4611628864,-0.9816200884
O,0,-2.0930061328,-0.2851236791,-0.3643524945
N,0,-3.4626583573,-0.1930983257,-0.0322904101
C,0,-2.5558284677,-2.0872574895,0.8780450386
C,0,-3.6991065399,-1.2566722266,0.71225943
C,0,-5.0802869919,-1.4785093936,1.2513430848
H,0,-5.747087468,-0.6829907871,0.9078495939
H,0,-5.4843803771,-2.4418558664,0.9180085672
H,0,-5.0778588405,-1.4858568864,2.3477010487
C,0,-2.4611397279,-3.3955200941,1.6010514518
H,0,-1.6819929762,-4.0243553674,1.1582156376
H,0,-2.1960838361,-3.264675006,2.6568421426
H,0,-3.4104069134,-3.9431174573,1.5489315312

SIZ
M062X/6-311+G(d,2p) EmpiricalDispersion=GD3
EE (a.u.)= -1214.90857129
B3LYP/6-31+G(d,p)
Thermal corr. to G (a.u.)= 0.190064
Gsolv(kcal/mol)= -18.06
S,0,2.1596143191,-2.0722508363,-0.3059163008
O,0,1.9472532781,-3.211161079,-1.2029120468
O,0,3.045422186,-2.1312779682,0.857643825
N,0,2.858608817,-0.8812238327,-1.3505172845
N,0,-3.1357690693,0.0649345848,1.4082415699
C,0,0.5749271045,-1.4612009187,0.2269119991
C,0,-0.5646511977,-1.7336665692,-0.5396346378
C,0,0.4783498765,-0.6984189489,1.3966234838
C,0,-1.9151566066,-0.4636887938,1.0343870842
C,0,-1.7985610303,-1.2405318565,-0.1375857619
C,0,-0.7566974525,-0.204577676,1.795635143
H,0,2.3920279149,-0.898937358,-2.2545160482
H,0,-0.4836184986,-2.3464865296,-1.4311517515
H,0,1.3630399127,-0.5109411334,1.9949343113
H,0,-2.6838155328,-1.4567956566,-0.7293941465
H,0,-0.831637671,0.386924034,2.7039667292
H,0,-3.2413162337,0.3933210348,2.3564046801
H,0,-3.9706395443,-0.3363687073,1.0083089717
C,0,3.1653759973,0.3931825533,-0.8824080736
C,0,3.9159962798,2.269513069,-0.0407941061
O,0,2.315166492,1.3998070942,-1.2165487654
C,0,4.205469008,0.8676238893,-0.1343401822
C,0,5.3837931068,0.1282275558,0.4159570877
H,0,5.4364831005,-0.8774865558,-0.005421022
H,0,6.3168333043,0.6554947666,0.1876203213
H,0,5.3194220945,0.0199007514,1.5045176665
C,0,4.7159130848,3.3252671925,0.6556645168
H,0,4.7920372299,3.1145637197,1.7281007196
H,0,5.7350989427,3.3709751748,0.2561502729
H,0,4.2444217048,4.3013502721,0.5222133389
N,0,2.8158570826,2.5962757278,-0.6871905939

SIZ-PhN-Cl
M062X/6-311+G(d,2p) EmpiricalDispersion=GD3
EE (a.u.)= -1674.45718162
B3LYP/6-31+G(d,p)
Thermal corr. to G (a.u.)= 0.177615
Gsolv(kcal/mol)= -16.68
S,0,0.2057890207,1.9099829616,0.2011300263
O,0,0.7367205459,3.1299798243,-0.4105560944
O,0,-0.4211733297,1.8876522345,1.5221467967
N,0,-1.0240582943,1.4173174832,-0.9050987396
N,0,4.5201738831,-2.224498645,0.0842950526
C,0,1.4813649866,0.6581650479,0.1727552801
C,0,2.5163250173,0.752691989,-0.7633601477
C,0,1.4207612969,-0.4144488835,1.0666492165
C,0,3.4406876813,-1.322312559,0.0904123931
C,0,3.4880686787,-0.2394018822,-0.8088431688
C,0,2.3994931956,-1.4028890867,1.0278116756
H,0,-0.7210197522,1.5868289192,-1.8614356986
H,0,2.5625728088,1.5977719662,-1.4417772525
H,0,0.6222028477,-0.464770763,1.7982778492
H,0,4.2956645545,-0.1681082826,-1.5323751007
H,0,2.357811549,-2.2342205443,1.7212798073
H,0,4.9984132374,-2.2593283547,-0.8112908465
C,0,-1.7320412112,0.232945594,-0.716182134
C,0,-3.0244834322,-1.4637012,-0.2240956682
O,0,-1.3736264963,-0.8324336976,-1.4807200327
C,0,-2.7714916336,-0.0906213598,0.1082723519
C,0,-3.5031421916,0.7735885178,1.0857994385
H,0,-3.2117744877,1.8190060855,0.9677242376
H,0,-4.5865843399,0.6917848416,0.9442571693
H,0,-3.2800070511,0.4891182999,2.120229306
C,0,-4.0699472359,-2.3670867671,0.3503571305
H,0,-3.9259336258,-2.4953678078,1.4287832973
H,0,-5.0720795865,-1.9509164246,0.1991290182
H,0,-4.022583055,-3.3471779471,-0.1291000323
N,0,-2.2191269952,-1.9045592852,-1.1682804094
Cl,0,4.1734081146,-3.8939150948,0.5186225588

SIZ-TS'$_{PhN-Cl-2w}$
M062X/6-311+G(d,2p) EmpiricalDispersion=GD3
EE (a.u.)= -1903.69932595
B3LYP/6-31+G(d,p)
Thermal corr. to G (a.u.)= 0.237308
Gsolv(kcal/mol)= -28.16
S,0,2.2601024606,-2.2618223018,-0.4564244337
O,0,2.0655738999,-3.1532979794,-1.6003252856
O,0,2.9998416834,-2.6493338104,0.7438028955
N,0,3.119810367,-0.9276623371,-1.1184353273
N,0,-3.115796744,-0.2196618551,1.2066231264
C,0,0.6499491615,-1.6546333529,0.0563864648
C,0,-0.4233847491,-1.7301965488,-0.8375246503
C,0,0.4970669376,-1.1109729155,1.334555023
C,0,-1.8335393829,-0.6987956249,0.8309622406
C,0,-1.6709212069,-1.2530193215,-0.4477422644
C,0,-0.7496384376,-0.6229690453,1.7166977336
H,0,2.7413900001,-0.6699189572,-2.027032136
H,0,-0.2845026599,-2.1806709838,-1.8141766292
H,0,1.3367846422,-1.0804419293,2.0194675744
H,0,-2.5241336977,-1.3149369737,-1.1151152423
H,0,-0.8808995077,-0.1871409456,2.7032636037
H,0,-3.2072358526,-0.0953588433,2.2129033828
Cl,0,-3.6578048962,1.5284184069,0.4177908959
O,0,-6.1751849105,0.9217283099,-1.0967791327
H,0,-6.4230046455,0.7769330793,-2.0178731418
H,0,-4.0436517926,-0.7291418765,0.6983280043
O,0,-5.1025691791,-1.0248260944,-0.0997501071
H,0,-5.7448323994,-1.6654358945,0.2279120183
H,0,-5.6508183495,-0.0877826487,-0.6006573978
H,0,-5.5458344571,1.9805277048,-0.9621157099
O,0,-4.8220168934,2.9082849961,-0.746851319
H,0,-5.2451572487,3.5213042586,-0.1273788686
C,0,3.5360624975,0.1337526911,-0.3176162855
C,0,4.4454758234,1.6148571008,1.0126142915
O,0,2.7758604079,1.2610156084,-0.3262227692
C,0,4.6115168251,0.280852045,0.5101325916
C,0,5.7127268351,-0.6881183721,0.8014692038
H,0,5.6718496284,-1.5343842935,0.1127251509
H,0,6.6923956358,-0.2067447826,0.7076739466
H,0,5.6342682888,-1.0905077668,1.817587544
C,0,5.3321299806,2.3526804956,1.9656824191
H,0,5.3911133001,1.8321105949,2.9278208638
H,0,6.3509519013,2.4310694244,1.5705210123
H,0,4.9446199116,3.3597034125,2.1342365399
N,0,3.3772052425,2.2035723667,0.5154029138

SIZ-TS'$_{SO2N-Cl-2w}$
M062X/6-311+G(d,2p) EmpiricalDispersion=GD3
EE (a.u.)= -1903.70086150
B3LYP/6-31+G(d,p)
Thermal corr. to G (a.u.)= 0.240154
Gsolv(kcal/mol)= -33.80
O,0,-0.1917458536,-2.0689478695,1.7714376339
O,0,-0.782485999,0.2258776788,2.7355248753
N,0,0.790817011,0.0039562344,0.713969247
N,0,-5.3530674131,-0.3899838962,-1.9552737302
C,0,-1.993831565,-0.5805009979,0.5314241101
C,0,-2.294511244,-1.6556389396,-0.3116345626
C,0,-2.8197615,0.5483413203,0.5661185092
C,0,0.8141215982,1.3858513399,0.5531182918
C,0,-4.2594102702,-0.4666648215,-1.1093719404
C,0,-3.4196975904,-1.5990108005,-1.124019284
C,0,-3.942309171,0.6039869352,-0.2490304007
H,0,-1.6571180337,-2.5323740297,-0.3130530203
H,0,-2.5878177983,1.3696513499,1.2345560716
H,0,-3.6560055617,-2.4348703853,-1.7771550554
H,0,-4.5814241443,1.4823913336,-0.2250230943
S,0,-0.5590484338,-0.6597129052,1.5823236275
O,0,3.6730056489,-2.7594006803,-0.3466184346
H,0,3.115941245,-2.6585007275,-1.2257083675
O,0,2.2210436112,-2.2331422761,-2.3592604765
Cl,0,1.2819436396,-1.074943315,-1.0635959476
H,0,1.5447980421,-2.8482064407,-2.6820103219
H,0,3.6455954522,-3.6827307622,-0.0637297411
H,0,3.2967936855,-1.9405395066,0.5708905278
H,0,2.0923713862,-0.6849572481,1.2059271229
O,0,3.0104724816,-1.1955080283,1.4001853825
H,0,3.7124206255,-0.5414361348,1.5248900256
H,0,-6.0664799892,0.289885396,-1.7374147702
H,0,-5.7083432869,-1.2520021311,-2.3416291562
O,0,0.087732421,1.9314220851,-0.4573417008
N,0,0.2851465668,3.314531502,-0.4586615623
C,0,1.1113407233,3.5636444763,0.5364144639
C,0,1.4894784721,2.3693566962,1.2288899153
C,0,1.5345247897,4.9699414847,0.8256076661
H,0,2.6212776342,5.080102109,0.7387174585
H,0,1.0576144654,5.6550511643,0.1211847578
H,0,1.2529466841,5.2625885357,1.8434320916
C,0,2.3686205913,2.2482828797,2.4343281022
H,0,3.4239464697,2.4362221674,2.1953470456
H,0,2.0812575316,2.9729255052,3.203955666
H,0,2.2782347276,1.2569025723,2.8860958647

SIZ-ISZ-Cl
M062X/6-311+G(d,2p) EmpiricalDispersion=GD3
EE (a.u.)= -1674.43932501
B3LYP/6-31+G(d,p)
Thermal corr. to G (a.u.)= 0.176973
Gsolv(kcal/mol)= -20.08
S,0,-0.3640187771,1.7262382825,-0.5961599174

O,0,-0.7452239185,2.9986564417,0.0195320411
O,0,-0.0605257721,1.6501580679,-2.0317313498
N,0,1.0006473549,1.2229572907,0.3130119799
N,0,-4.550095248,-2.3108929127,0.7592529858
C,0,-1.6170494838,0.5184623831,-0.2007855505
C,0,-2.38911636,0.6834379377,0.9549317821
C,0,-1.8311840459,-0.5732223509,-1.0486308454
C,0,1.7283550727,0.2287950874,-0.0380623437
C,0,-3.5997664872,-1.3575714984,0.4253083859
C,0,-3.370343782,-0.2494765246,1.2664564276
C,0,-2.8165657569,-1.5028163762,-0.73726386
H,0,-2.2277650617,1.5475448071,1.5904758037
H,0,-1.2383145664,-0.6794982757,-1.9501053455
H,0,-3.9723047694,-0.1207884221,2.1621706357
H,0,-2.9872817591,-2.3483692696,-1.3983714592
H,0,-4.882090743,-2.9111820771,0.0183159524
H,0,-5.2696208145,-2.0369852249,1.4125395923
O,0,1.4518089999,-0.650355907,-1.0623082574
N,0,2.4207904399,-1.6920899482,-1.0271065084
C,0,3.3637333773,-1.3238710159,-0.02500905
C,0,2.9406166715,-0.2196637436,0.6314551504
C,0,3.4995864033,0.4714834261,1.8301273254
H,0,3.7768635424,1.500300576,1.5775811157
H,0,2.7460785842,0.5332249632,2.6221112187
H,0,4.3812899397,-0.0425642596,2.2182543769
C,0,4.5423602624,-2.2110113312,0.1873674859
H,0,5.2599225167,-2.108708582,-0.6335923231
H,0,5.0457225776,-1.9549366673,1.1212625326
H,0,4.2313935264,-3.2594425251,0.2297097564
Cl,0,3.1878670968,-1.7361911115,-2.684129648

SIZ-PhCCl
M062X/6-311+G(d,2p) EmpiricalDispersion=GD3
EE (a.u.)= -1674.50915546
B3LYP/6-31+G(d,p)
Thermal corr. to G (a.u.)= 0.178645
Gsolv(kcal/mol)= -16.49
S,0,0.234999553,1.9192321694,0.1413473998
O,0,0.7826852945,3.1077371283,-0.5162628388
O,0,-0.4128228571,1.9602907764,1.4521547219
N,0,-0.9845832188,1.3894783277,-0.9629011492
N,0,4.4226824328,-2.3306945561,0.2596892148
C,0,1.4962016221,0.6597621545,0.1880552884
C,0,2.5282931558,0.6721062487,-0.7576142682
C,0,1.4262025263,-0.3455331509,1.1556919633
C,0,3.4460577105,-1.3671211363,0.2222821054
C,0,3.4869419563,-0.329006097,-0.7348377929
C,0,2.3931824634,-1.3408206847,1.1652844101
H,0,-0.6688797962,1.5334237618,-1.9194030929
H,0,2.5864986539,1.4708184086,-1.4890097333
H,0,0.6371341992,-0.3414557152,1.8982459078
H,0,4.2974247229,-0.3164774371,-1.4581365658
H,0,5.011272267,-2.447552307,-0.5503327854
C,0,-1.6750445512,0.1985772866,-0.7522005215
C,0,-2.9392791301,-1.5100738011,-0.228689404
O,0,-1.3028260701,-0.8731972179,-1.5009938062
C,0,-2.7068424284,-0.1280556211,0.080887689
C,0,-3.4502202014,0.7399125096,1.0463595913
H,0,-3.1749688285,1.7876900682,0.9120154361
H,0,-4.5324853914,0.6391949674,0.9077415346
H,0,-3.2217719471,0.4747740729,2.084801666
C,0,-3.9677739729,-2.4204520574,0.3649683567
H,0,-3.8157744892,-2.529876434,1.4443613154
H,0,-4.9770345868,-2.0221886831,0.2128707022
H,0,-3.9075857906,-3.4069982856,-0.0995919207
N,0,-2.1307883421,-1.9535265042,-1.168868962
H,0,4.2611875032,-3.1593187004,0.8124733776
Cl,0,2.3095897008,-2.6051319212,2.3858236005

SIZ-Cl_{Int}
M062X/6-311+G(d,2p) EmpiricalDispersion=GD3
EE (a.u.)= -1674.45194030
B3LYP/6-31+G(d,p)
Thermal corr. to G (a.u.)= 0.177409
Gsolv(kcal/mol)= -19.16
S,0,-0.3967790439,2.0263765396,0.4876351331
O,0,-0.106668392,3.4144763824,0.12391781
O,0,-0.8500334286,1.6326968286,1.8193020852
N,0,-1.6319623086,1.5740821199,-0.6087552306
N,0,4.7225399587,-0.6642255799,-0.4903674985
C,0,1.0435711868,1.0277245401,0.0797236147
C,0,1.7653830953,1.3341737419,-1.1512817604
C,0,1.4126632345,0.0415214559,0.9172334455
C,0,3.5032952045,-0.2685874356,-0.4985493587
C,0,2.9183233936,0.6948517766,-1.4400325122
C,0,2.5927002627,-0.8163028761,0.5899940232
H,0,-1.5297934402,2.0691902827,-1.4910693306
H,0,1.3767893121,2.1046916681,-1.8088324197
H,0,0.8486736891,-0.170470325,1.8188746397
H,0,3.4784866123,0.9353285258,-2.3394641301
H,0,5.2442033053,-0.2620106127,-1.2752587594
C,0,-2.1032585535,0.2650881081,-0.702134788
C,0,-3.011005672,-1.7230505503,-0.6171354434
O,0,-1.6917315054,-0.4534328225,-1.7787126205
C,0,-2.9413430719,-0.4711046341,0.0839914826
C,0,-3.654932054,-0.0765794758,1.3381720331
H,0,-3.5460432873,0.9932418584,1.5254978811
H,0,-4.7221456326,-0.3149767557,1.2698809247
H,0,-3.2530405981,-0.60345527,2.2109212588
C,0,-3.7934482323,-2.9408712829,-0.2392303542
H,0,-3.4624131078,-3.331408413,0.7292473995
H,0,-4.8610413156,-2.7097560179,-0.1552619642
H,0,-3.6618729527,-3.7189453702,-0.9940526337
N,0,-2.2945992366,-1.7159924352,-1.7212356523
H,0,3.1885620178,-1.0602163095,1.4692223198
Cl,0,1.9133636707,-2.4486035117,0.0365135254

SIZ-TS_Cl_1
M062X/6-311+G(d,2p) EmpiricalDispersion=GD3
EE (a.u.)= -1673.886876
B3LYP/6-31+G(d,p)
Thermal corr. to G (a.u.)= 0.158191
O,0,-0.3401647528,3.2036210217,-1.1804949367
O,0,-1.4117117329,2.4526441431,0.9882988329
N,0,-1.3203336584,0.9034475216,-1.1492902496
N,0,4.2852204481,-0.8331123393,0.9069640263
C,0,0.8087954412,1.2573988223,0.1699939205
C,0,1.8940752612,1.2946158112,-0.7659196561
C,0,0.889789907,0.5224633631,1.3644609242
C,0,3.1808034746,-0.1708293612,0.7301350184
C,0,3.0477458256,0.6327092259,-0.483231461
C,0,2.0425248418,-0.179527627,1.6615423976
H,0,1.7713402082,1.8774247574,-1.6719172042
H,0,0.0599780459,0.5508951849,2.0624111674
H,0,2.1729293003,-0.6462867539,2.6276569207
H,0,4.1732172999,-1.4654149386,1.7098347676
Cl,0,2.0582496228,-3.0671791121,2.1330740873
H,0,3.8966958327,0.6412455504,-1.1593900483
C,0,-2.1777953292,-0.0258267661,-0.7043771146
C,0,-3.4682676123,-1.8104028132,-0.4760041789
O,0,-2.9134447138,0.0857780389,0.4419990878
N,0,-3.7305723972,-1.0484700201,0.5724150172
C,0,-2.5005963753,-1.2290897808,-1.3331015154
C,0,-4.1663425634,-3.126438989,-0.6290644756
H,0,-4.7044228013,-3.1792478625,-1.5827420264
H,0,-3.4468106132,-3.9527823979,-0.6076434589
H,0,-4.8808243956,-3.2666961492,0.1857761132
C,0,-1.9111990811,-1.7676961607,-2.5945916813
H,0,-1.3008499058,-0.9998391728,-3.0767576366
H,0,-1.267096301,-2.6360277826,-2.4017829903

H,0,-2.6873627065,-2.0873161209,-3.301813244
S,0,-0.6946436198,2.1180883076,-0.2515902531

SIZ-NClN
M062X/6-311+G(d,2p) EmpiricalDispersion=GD3
EE (a.u.)= -1674.37714419
B3LYP/6-31+G(d,p)
Thermal corr. to G (a.u.)= 0.177556
Gsolv(kcal/mol)= -25.71
N,0,1.2497132934,0.9755741941,-1.1024932107
O,0,2.5814262373,1.4424948521,1.157515209
S,0,2.5354513914,0.6044405059,-0.0455908132
O,0,3.7112882235,0.4542183349,-0.8928400098
C,0,1.7589978844,-0.9378749474,0.3009029894
C,0,0.8302248726,-0.9756722386,1.3602460278
C,0,1.7669619868,-1.9513964837,-0.6949299407
C,0,-0.1594355859,-1.9277665613,1.3635412456
H,0,0.8776776292,-0.2258914864,2.1414177018
C,0,0.815401519,-2.9432811923,-0.6681909114
H,0,2.5005432466,-1.9032408116,-1.4925795742
C,0,-0.2300077213,-2.8961756068,0.3008847404
H,0,-0.9054731848,-1.9581403488,2.1469808702
H,0,0.7960244457,-3.7074759877,-1.439145662
N,0,-1.2904123608,-3.7189837719,0.2569318459
H,0,-1.4060035656,-4.3105121281,-0.5564254381
H,0,-2.1627305422,-3.3223201437,0.6410577247
C,0,0.0636182508,1.4126924179,-0.787714091
C,0,-1.982285668,2.0196455662,-0.1180752594
O,0,-0.8901410501,1.087865697,-1.758578569
N,0,-2.1202912916,1.4296992938,-1.3139079824
C,0,-0.6327948428,2.0744208839,0.2723730246
C,0,-0.1140749511,2.7134898275,1.5123279934
H,0,0.9133047663,3.0583387455,1.4006460078
H,0,-0.7608605919,3.5479736099,1.7982228735
H,0,-0.1216363206,2.0027740415,2.3499136282
C,0,-3.1937741486,2.4928348118,0.6149736384
H,0,-3.3797416885,1.851210392,1.4818713034
H,0,-3.0704009578,3.5250754496,0.9577856927
H,0,-4.0653755063,2.4329591194,-0.0387578302
Cl,0,-3.2134477691,-1.2956510348,0.6294717753

SMX^-
M062X/6-311+G(d,2p) EmpiricalDispersion=GD3
EE (a.u.)= -1175.05657718
B3LYP/6-31+G(d,p)
Thermal corr. to G (a.u.)= 0.151796
Gsolv(kcal/mol)= -67.92
S,0,2.4646832332,-2.0053225677,-0.237205529
O,0,3.9141036681,2.0665237243,0.9107988206
O,0,2.2548023334,-3.1032615417,-1.2126695166
O,0,3.0909018517,-2.3701604613,1.0591830805
N,0,3.2051909531,-0.8146880883,-1.0391105174
N,0,3.3080663498,0.7644282735,0.8078384121
N,0,-3.1231713654,-0.104851923,1.0753666365
C,0,0.7843341167,-1.4384248266,0.1782333191
C,0,-0.273183404,-1.7266646786,-0.6875585569
C,0,0.5425503797,-0.712990077,1.3478563746
C,0,3.5779744376,0.3450361949,-0.4314152492
C,0,-1.8214741429,-0.5768935691,0.789587338
C,0,-1.5673548993,-1.3001305646,-0.3864466437
C,0,-0.751500216,-0.2865639047,1.6498671964
C,0,4.3483933539,1.3462415724,-1.1432518622
C,0,4.5148992759,2.3622489132,-0.2631483446
C,0,5.2071907064,3.6828017391,-0.3406253006
H,0,-0.0712402036,-2.3015771288,-1.5854448909
H,0,1.3704235771,-0.481465196,2.007932074
H,0,-2.3872744626,-1.5306755219,-1.0648001186
H,0,-0.9331807278,0.2819527433,2.560380856
H,0,4.7038679513,1.2670479384,-2.1597719519
H,0,4.5060273784,4.5071367296,-0.1615206596
H,0,6.0010589896,3.7565846044,0.4126851569
H,0,5.654511801,3.8172009656,-1.3292696557
H,0,-3.2922893489,0.0509477872,2.0613892025
H,0,-3.8602485865,-0.6693121366,0.6713403297

SMX^--PhNCl
M062X/6-311+G(d,2p) EmpiricalDispersion=GD3
EE (a.u.)= -1634.61171034
B3LYP/6-31+G(d,p)
Thermal corr. to G (a.u.)= 0.138934
Gsolv(kcal/mol)= -60.64
S,0,0.144533517,1.8297130016,0.1812356558
O,0,-2.8786093265,-1.4080014413,0.7552774265
O,0,0.7320770417,2.9735610537,-0.5541099257
O,0,-0.0967957761,2.0125921048,1.6332579584
N,0,-1.1227935689,1.3241755688,-0.6725062117
N,0,-1.753209306,-0.5176123217,0.7830037049
N,0,4.4325700202,-2.4007993308,-0.1834371438
C,0,1.4155333675,0.5225241522,0.0695644548
C,0,2.391587246,0.5994305554,-0.9335171673
C,0,1.4017006611,-0.5514327987,0.9644306474
C,0,-1.9316654354,0.3111630887,-0.2474183976
C,0,3.3640316632,-1.4683661782,-0.136614609
C,0,3.3680023616,-0.3862229237,-1.0328441675
C,0,2.3624016023,-1.5551617158,0.8441424035
C,0,-3.1514455334,-0.0193656953,-0.9541723465
C,0,-3.6752417442,-1.0746775186,-0.284991396
C,0,-4.9073323464,-1.896981767,-0.4680771622
H,0,2.3828080727,1.4531172261,-1.6027326263
H,0,0.62879741,-0.6014011943,1.72227105
H,0,4.1481732994,-0.3279603307,-1.7860393536
H,0,2.343466742,-2.4084125151,1.5198319295
H,0,-3.5383017235,0.4893742894,-1.8242673045
H,0,-4.6565136661,-2.9529312398,-0.6254569922
H,0,-5.5538819074,-1.8398055573,0.4158720516
H,0,-5.4722240016,-1.5421236755,-1.3341826909
H,0,4.371066034,-3.003778016,0.6372441538
Cl,0,4.1990613167,-3.6896651307,-1.5247569715

$TS_{PhN-Cl-1w}$
M062X/6-311+G(d,2p) EmpiricalDispersion=GD3
EE (a.u.)= -1787.36507725
B3LYP/6-31+G(d,p)
Thermal corr. to G (a.u.)= 0.174937
Gsolv(kcal/mol)= -69.53
S,0,2.0730683195,-2.0241905251,-0.1403163692
O,0,3.7914678404,1.9672821831,0.8821628723
O,0,1.8858192034,-3.1557939848,-1.0664612543
O,0,2.7332693979,-2.2859947035,1.1539575312
N,0,2.7144286547,-0.806369707,-1.0109909837
N,0,3.1328772198,0.7179124305,0.8303239169
N,0,-3.3735724493,0.0372744821,1.0099993755
C,0,0.4278685073,-1.4000022771,0.2145599561
C,0,-0.636431253,-1.6940978726,-0.6636242391
C,0,0.2256557099,-0.5708170096,1.3393032583
C,0,3.2236860099,0.323176472,-0.4440611075
C,0,-2.1297392607,-0.4001266492,0.7485804868
C,0,-1.9007056718,-1.205552059,-0.4084659806
C,0,-1.0330094479,-0.0747030987,1.6020492135
C,0,3.9313937408,1.3075311006,-1.2300862092
C,0,4.2564678542,2.2872848476,-0.3522863196
C,0,4.9850446628,3.5823459722,-0.4705522084
H,0,-0.4406060059,-2.3255654505,-1.5232009349
H,0,1.0688365253,-0.3351064284,1.9785000324
H,0,-2.7423167478,-1.4265414152,-1.0576205535
H,0,-1.2055385924,0.5636694083,2.4647113868
H,0,4.1448301011,1.2448094438,-2.2858928692
H,0,4.3436232263,4.4206031269,-0.1756007269
H,0,5.8688233416,3.5958784163,0.1775440995
H,0,5.3079225652,3.7383976386,-1.5026646931

H,0,-3.5493354031,0.5359652148,1.8724173606
Cl,0,-4.5261827566,2.1345371735,-0.0041215413
O,0,-6.8267201835,1.0707230421,-1.1064719723
H,0,-7.1716206898,1.5195174234,-0.3209137888
H,0,-4.2280219603,-0.3262427827,0.4689447248
O,0,-5.4345574595,-0.842530447,-0.3903199059
H,0,-6.0166157432,-1.5608705786,-0.1192461229
H,0,-6.0659322553,0.0065606134,-0.7118324341

$TS_{PhN\text{-}Cl\text{-}2w}$
M062X/6-311+G(d,2p) EmpiricalDispersion=GD3
EE (a.u.)= -1863.86469584
B3LYP/6-31+G(d,p)
Thermal corr. to G (a.u.)= 0.201057
Gsolv(kcal/mol)= -72.41
S,0,0.5188400362,-3.0212235856,0.4441640028
O,0,-2.53017323,-0.0930213143,-0.9850250517
O,0,1.1555676487,-3.873247461,1.473573883
O,0,0.1962268914,-3.656035894,-0.8563392806
N,0,-0.6908048829,-2.2397413211,1.155665372
N,0,-1.4051367607,-0.9558145077,-0.7790070172
N,0,4.7829217519,1.0423060411,-0.8549265101
C,0,1.7949837901,-1.7726585532,0.0498240793
C,0,2.8532770809,-1.5674106655,0.9429133506
C,0,1.7037229093,-1.0250291698,-1.1307641988
C,0,-1.5290528188,-1.4086783548,0.4694562468
C,0,3.7522931679,0.1068295742,-0.5335435507
C,0,3.8395460608,-0.625822051,0.6571901668
C,0,2.6806373773,-0.0756827542,-1.417406758
C,0,-2.7133568398,-0.8567395417,1.0906554817
C,0,-3.2744651642,-0.0659989903,0.1439024713
C,0,-4.4996315026,0.7853055159,0.1111700947
H,0,2.8986508843,-2.1706068401,1.8431904344
H,0,0.8661498547,-1.1875849954,-1.7987320281
H,0,4.6765325719,-0.464482511,1.3299563533
H,0,2.616256934,0.5182898122,-2.3268683709
H,0,-3.0545006149,-1.0550306585,2.0954271604
H,0,-4.2456759924,1.8328998001,-0.0898528017
H,0,-5.187261935,0.4550359158,-0.6765209853
H,0,-5.0205488655,0.7328843524,1.0707305765
H,0,4.8326694137,1.2196654571,-1.8561348368
Cl,0,4.7263927575,2.8426614201,-0.0901408341
O,0,7.4979731911,3.235281377,1.2632549179
H,0,7.8116630672,3.1959786574,2.1747358621
H,0,5.768893196,0.8384193422,-0.4472790379
O,0,7.1115341106,0.9752866624,0.3267871995
H,0,7.9457166646,0.6249445168,-0.0040609703
H,0,7.3007854703,1.9726744182,0.7570253618
H,0,6.4647099791,4.0529011546,1.1837855808
O,0,5.5259842509,4.6767669459,1.0522144349
H,0,5.7178128562,5.3510263959,0.385966612

$TS_{PhN\text{-}Cl}$
M062X/6-311+G(d,2p) EmpiricalDispersion=GD3
EE (a.u.)= -1940.31115017
B3LYP/6-31+G(d,p)
Thermal corr. to G (a.u.)= 0.221041
Gsolv(kcal/mol)= -74.15
S,0,2.5034004801,-2.0412937316,-0.3834767938
O,0,4.1583494311,1.8543516302,1.0254333981
O,0,2.2168867756,-3.0628682556,-1.4157773649
O,0,3.1176805786,-2.5097306622,0.8819570673
N,0,3.2564441777,-0.8254559575,-1.1144201427
N,0,3.4719568195,0.6118165682,0.8318236998
N,0,-2.9773854702,-0.0597596983,1.1947943925
C,0,0.8506066201,-1.425356691,0.1006069151
C,0,-0.2388536272,-1.6488443754,-0.7486451885
C,0,0.6848418913,-0.7285690688,1.3036816675
C,0,3.7150617312,0.2653133017,-0.4329682406
C,0,-1.6684322144,-0.5058965288,0.812671622
C,0,-1.5072541342,-1.1897470518,-0.3980632509
C,0,-0.5784006642,-0.260065713,1.6561418233
C,0,4.5458503399,1.2592358469,-1.0771630886
C,0,4.7785252615,2.1967197008,-0.1265301186
C,0,5.5544532049,3.4712888438,-0.1093450963
H,0,-0.0777794946,-2.20336097,-1.6668114029
H,0,1.545491743,-0.5519333422,1.9378561149
H,0,-2.3674435239,-1.3722452809,-1.0349950283
H,0,-0.7181847558,0.2929437173,2.5826741863
H,0,4.895458045,1.2299461012,-2.0980512405
H,0,4.9073738581,4.3228237643,0.1325527192
H,0,6.3504608618,3.4369743211,0.6439943124
H,0,6.0097890636,3.6484645775,-1.0871716981
H,0,-3.0583107318,0.0221502455,2.2068021934
O,0,-6.6993137484,0.1644984259,-0.9357104471
H,0,-6.8344168364,-0.0813263944,-1.8586649409
H,0,-3.7933763346,-0.6537950798,0.8019883818
O,0,-5.0100730598,-1.3151327701,0.1326054266
H,0,-5.4945243822,-2.0330583045,0.5548849886
H,0,-5.7268466835,-0.6680419603,-0.3239333062
H,0,-6.6337948884,1.3504603437,-0.9004512435
O,0,-6.5748899359,2.5842622974,-0.9066329795
H,0,-7.3633820584,2.9476083254,-0.4874452401
H,0,-5.4037581274,3.2029323832,-0.4728757174
O,0,-4.4717319619,3.6341567437,-0.1394468761
H,0,-3.9704818192,3.8666181782,-0.9330375069
Cl,0,-3.5387970707,1.6952600602,0.5949854136

$TS_{SO2N\text{-}Cl\text{-}1w}$
M062X/6-311+G(d,2p) EmpiricalDispersion=GD3
EE (a.u.)= -1787.36997832
B3LYP/6-31+G(d,p)
Thermal corr. to G (a.u.)= 0.178971
Gsolv(kcal/mol)= -65.31
O,0,1.3177,3.032855,-0.364592
O,0,-0.589607,0.632484,2.75616
N,0,0.516514,2.047286,0.216997
N,0,-5.16615,0.220175,-1.978671
C,0,-1.87609,-0.213292,0.598018
C,0,-2.369371,-1.325226,-0.090164
C,0,-2.463484,1.042423,0.429864
C,0,1.320406,0.992046,0.368642
C,0,-4.05037,0.080105,-1.1394
C,0,-3.452155,-1.177753,-0.952655
C,0,-3.547632,1.185722,-0.431546
C,0,2.643243,1.258477,-0.133296
C,0,2.575121,2.535675,-0.575916
C,0,3.549046,3.463777,-1.215704
H,0,-1.901528,-2.291579,0.054419
H,0,-2.060179,1.896099,0.960668
H,0,-3.83654,-2.042469,-1.488722
H,0,-4.007093,2.162925,-0.562155
H,0,3.432073,0.517326,-0.160197
H,0,4.512428,2.962098,-1.331224
H,0,3.20037,3.780321,-2.20571
H,0,3.6941,4.365306,-0.608903
H,0,-5.241349,-0.480732,-2.704166
H,0,-5.306434,1.152372,-2.34545
S,0,-0.497173,-0.425001,1.730202
O,0,-0.506601,-1.832201,2.146154
N,0,0.985084,-0.210561,0.932461
Cl,0,1.012636,-1.727097,-0.931051
H,0,2.923246,-1.812912,0.906441
O,0,3.374814,-1.624272,0.068666
O,0,3.923823,-3.786021,-1.479017
H,0,3.044624,-3.867882,-1.869079
H,0,3.804788,-3.012983,-0.86282

$TS_{SO2N\text{-}Cl\text{-}2w}$*

M062X/6-311+G(d,2p) EmpiricalDispersion=GD3
EE (a.u.)= -1863.88715046
B3LYP/6-31+G(d,p)
Thermal corr. to G (a.u.)= 0.199940
Gsolv(kcal/mol)= -69.98
O,0,-6.198792221,4.2969497605,7.4573415041
O,0,-7.159023251,4.8830697121,5.1733513816
N,0,-5.3284251387,3.7467562789,3.1335233325
N,0,-7.950233278,-1.6815361093,5.3897286142
C,0,-6.8110702765,2.3319738356,5.8237179174
C,0,-6.5661230153,1.426267065,6.8606870846
C,0,-7.4380425992,1.9142951321,4.6458862744
C,0,-4.416894346,4.066545529,4.0329532174
C,0,-7.5986860907,-0.3383895467,5.5477605993
C,0,-6.9579420475,0.0982764639,6.7214448127
C,0,-7.8325923134,0.5868261526,4.5139738471
C,0,-3.1236136056,4.2851149128,3.4645991763
C,0,-3.3360884,4.084723803,2.1384914999
C,0,-2.4208069935,4.1444041913,0.9663019336
H,0,-6.0823001,1.7686082943,7.7684672179
H,0,-7.5980421017,2.6255505256,3.8445110985
H,0,-6.7642169607,-0.6081058808,7.5250375631
H,0,-8.3164986473,0.2582478281,3.5973003809
H,0,-2.1643669027,4.5188120622,3.9330190542
H,0,-1.413383082,4.368732857,1.3252814492
H,0,-2.4043074623,3.1881455406,0.4302562332
H,0,-2.7328642132,4.9206172436,0.2572128681
S,0,-6.3119364509,4.040441292,6.0201494653
H,0,-8.6748231246,-1.8705520574,4.7112252384
H,0,-8.067668225,-2.210378527,6.242761672
N,0,-4.7081039401,4.202886241,5.4087484883
Cl,0,-3.4273099806,3.4522379568,6.431208866
O,0,-1.4485308797,2.4424544535,7.566414387
O,0,-4.6418869138,3.7748236046,1.9067915264
H,0,-1.5774011059,1.500493429,7.4001384668
H,0,-0.7214547384,2.7276827017,6.8700969139
O,0,0.2720423507,3.041104254,5.8077489157
H,0,1.1787058982,3.1356855909,6.1208189366
H,0,0.0583993418,3.9412535608,4.9583460628
O,0,-0.1606430996,4.8019697814,4.166055672
H,0,-0.0431008725,5.7015967152,4.5178069815

$TS_{SO2N\text{-}Cl}$
M062X/6-311+G(d,2p) EmpiricalDispersion=GD3
EE (a.u.)= -1940.311856
B3LYP/6-31+G(d,p)
Thermal corr. to G (a.u.)= 0.217407
Gsolv(kcal/mol)= -66.2

$TS_{ISX\text{-}Cl\text{-}2w}$
M062X/6-311+G(d,2p) EmpiricalDispersion=GD3
EE (a.u.)= -1863.88994135
B3LYP/6-31+G(d,p)
Thermal corr. to G (a.u.)= 0.201567
Gsolv(kcal/mol)= -62.55
N,0,-1.5824406205,4.6514781324,-7.1911722859
O,0,-3.7345665129,6.1019357343,-7.7138844717
O,0,1.5939507927,6.0665208251,-2.0492051136
Cl,0,-0.3980653003,3.2420470088,-4.5950517467
H,0,2.8575609371,6.6081132012,-3.052254299
S,0,-2.2534010939,6.1329986171,-7.6204402808
O,0,-1.4913245809,6.5961266134,-8.7889017388
C,0,-1.7938323762,7.0528111281,-6.1672580842
C,0,-2.7411205429,7.3068958721,-5.1718022989
C,0,-0.4275132514,7.2406651377,-5.9056412692
C,0,-2.3202979119,7.6992430247,-3.9035602008
H,0,-3.7948744781,7.1605678344,-5.385390707
C,0,-0.0085704578,7.6166271348,-4.6410879316
H,0,0.299484677,7.0427636497,-6.6862962976
C,0,-0.9459824087,7.7817024569,-3.593962221
H,0,-3.056221669,7.8669769836,-3.1199089194
H,0,1.049134029,7.722041729,-4.420389526
N,0,-0.4878458701,8.0084417369,-2.3077887573
H,0,0.3541159177,7.4433540722,-2.0915859426
H,0,-1.2006807832,7.8897735347,-1.5984563112
C,0,-2.2777512008,3.7644383748,-6.5445356756
N,0,-1.5678031318,2.6897025316,-5.9404471271
C,0,-3.6838766015,3.5450523173,-6.2969637194
O,0,-2.5602199134,1.8654534728,-5.3036957865
C,0,-3.7724431687,2.41024888,-5.5650358511
H,0,-4.479460973,4.1770058938,-6.6552754294
C,0,-4.9370003276,1.6458877977,-5.0387081044
H,0,-4.963289712,0.6394193408,-5.471199558
H,0,-4.8630391254,1.5390922732,-3.9510493737
H,0,-5.8671899825,2.1618772838,-5.2848708782
H,0,1.2654134882,4.9176679925,-2.5071789279
O,0,1.0478160339,3.8422713325,-2.8921171941
H,0,1.8864698227,3.5009416778,-3.2295501017
O,0,3.5679920755,6.8949458401,-3.6944653582
H,0,1.9514336655,5.9177668946,-1.1648842732
H,0,3.4299884351,6.3431501396,-4.4732022181

$TS_{ISX\text{-}Cl}$
M062X/6-311+G(d,2p) EmpiricalDispersion=GD3
EE (a.u.)= -1940.339865
B3LYP/6-31+G(d,p)
Thermal corr. to G (a.u.)= 0.224278
Gsolv(kcal/mol)= -61.86
N,0,1.796647411,-0.9345323699,-1.1964337106
O,0,2.3196957253,-2.8183386662,0.573863591
O,0,-3.1571233524,2.7603371299,-0.2364308424
Cl,0,0.7707837088,2.0874052958,-1.2592986531
H,0,-4.3156008714,1.6748075311,-0.8078532749
S,0,1.4762947129,-2.4618439608,-0.5948591448
O,0,1.4722949947,-3.3680413474,-1.7538531032
C,0,-0.1968444657,-2.2296761109,-0.0256794029
C,0,-0.4343751009,-1.8510534406,1.3004100395
C,0,-1.2432918911,-2.2186587365,-0.9556090295
C,0,-1.6993362051,-1.4218448012,1.682649397
H,0,0.3777465116,-1.8819406795,2.0194657252
C,0,-2.5073310802,-1.7900395959,-0.5724331621
H,0,-1.0487461173,-2.5176119916,-1.9803739793
C,0,-2.7491828311,-1.3265032776,0.7407441363
H,0,-1.8834585064,-1.1168137976,2.7091188571
H,0,-3.3132169435,-1.7551638855,-1.2999909864
N,0,-3.9783244818,-0.8076281782,1.103064448
H,0,-4.5105488636,-0.4295059541,0.318822035
H,0,-3.9025208052,-0.1023312382,1.833984303
C,0,2.411958528,-0.0369734916,-0.476796449
N,0,2.4544527726,1.2837202369,-0.9722600434
C,0,3.1508835796,-0.0511868482,0.7666047498
O,0,3.1382510292,2.0679398344,0.0153276604
C,0,3.5437308578,1.2244008657,0.9928545499
H,0,3.3375286402,-0.9269497297,1.365302215
C,0,4.3526191543,1.8478720389,2.077060698
H,0,5.2749769996,2.2777308637,1.6705224825
H,0,3.7914598476,2.6571209724,2.5567263999
H,0,4.6125007307,1.0996086025,2.8285892276
H,0,-2.1731222651,2.8639693105,-0.9079630227
O,0,-1.2046924188,3.0443906407,-1.6483265776
H,0,-1.4073315447,2.651551853,-2.5080011679
O,0,-4.9894981206,1.000846329,-1.1142153564
H,0,-3.5307464267,3.6490065052,-0.1815761722
H,0,-4.8008574607,0.8600422111,-2.0488983291
O,0,-2.9305772987,1.8475520328,2.3194712644
H,0,-1.9841670866,1.7053124648,2.4417107321
H,0,-3.0049788864,2.1945352628,1.3889696959

SMX-imide-E

M062X/6-311+G(d,2p) EmpiricalDispersion=GD3
EE (a.u.)= -1175.57960333
B3LYP/6-31+G(d,p)
Thermal corr. to G (a.u.)= 0.164342
Gsolv(kcal/mol)= -21.35
C,0,1.6597800948,-0.1037025968,0.5292007064
C,0,2.1434761781,-0.782832041,-0.5915327001
C,0,3.4309916713,-0.5265657497,-1.0519979197
C,0,4.2523376282,0.412409851,-0.3983296539
C,0,3.7489800948,1.0827365706,0.7345753172
C,0,2.4636052005,0.8251922273,1.1964530798
H,0,1.5156979207,-1.5152342945,-1.0875583695
H,0,3.811946711,-1.0643535653,-1.9163515086
H,0,4.3773828913,1.7981163999,1.2587203299
H,0,2.0839727764,1.3329395398,2.0765896409
N,0,5.5555256627,0.6311150304,-0.8263599008
H,0,5.7783414264,0.3682592948,-1.7754087654
H,0,5.9991815324,1.4881683747,-0.5295273194
S,0,0.0106495876,-0.4320436277,1.1242191859
O,0,-0.0016785273,-0.2525610627,2.5847019076
O,0,-0.4291495279,-1.7273163433,0.5602105175
N,0,-0.8308112244,0.8509310342,0.434860069
C,0,-2.0806176709,0.6843446731,0.1164704364
C,0,-3.0202996854,-0.4170290292,0.0818340788
C,0,-4.1483523124,0.0475682291,-0.5058782169
H,0,-2.8130951783,-1.4211706644,0.4125731917
N,0,-2.8078072779,1.7982681722,-0.3140450287
O,0,-4.0493815688,1.3654891604,-0.8416176627
C,0,-5.4515823212,-0.5893515998,-0.8319896638
H,0,-6.2602440011,-0.121976425,-0.2598383182
H,0,-5.6831108241,-0.4737492889,-1.8959183173
H,0,-5.4163084811,-1.6526219827,-0.5892673227
H,0,-2.3783993753,2.4619392136,-0.9505943533

$TS_{EZ\text{-}rotation}$
M062X/6-311+G(d,2p) EmpiricalDispersion=GD3
EE (a.u.)= -1175.55526569
B3LYP/6-31+G(d,p)
Thermal corr. to G (a.u.)= 0.163922
Gsolv(kcal/mol)= -25.62
C,0,1.73474924,-0.2253535511,-0.2268100311
C,0,2.4061656063,0.2966655545,-1.334376236
C,0,3.7344319931,0.6931628145,-1.2140001154
C,0,4.4105772222,0.569997789,0.0147441356
C,0,3.7204860011,0.0311821525,1.1180766726
C,0,2.3924987912,-0.3640702498,0.9975944675
H,0,1.8888115356,0.3796049634,-2.2840099943
H,0,4.2593011907,1.0912856058,-2.0786710744
H,0,4.2352573835,-0.0868624991,2.068210095
H,0,1.8669805181,-0.7909634884,1.8449758708
N,0,5.7537475709,0.9172921228,0.1218306691
H,0,6.1136059619,1.5575038938,-0.5715029775
H,0,6.1028105717,1.0998265038,1.0518253449
S,0,0.0283855853,-0.7254623856,-0.3824497212
O,0,-0.2346739102,-1.8202922304,0.5851556542
O,0,-0.2357097676,-1.0172081307,-1.8079768054
N,0,-0.7453397268,0.6309309414,0.1125085579
C,0,-2.0569728751,0.6392135985,0.2223707689
C,0,-3.1187539772,0.9565511361,-0.6954925343
C,0,-4.2907342084,0.8090411348,-0.0225858707
H,0,-2.9798257667,1.2270970173,-1.7301409363
N,0,-2.6861030029,0.4160673329,1.4210351189
O,0,-4.0714607944,0.4355368299,1.2716119493
C,0,-5.7180526643,0.9882590766,-0.3956290321
H,0,-6.2856439246,0.0724340967,-0.2016686232
H,0,-6.1708843568,1.7963649655,0.1887746076
H,0,-5.7947518122,1.2342244329,-1.4559559227
H,0,-2.3614777145,-0.2394734078,2.1251152022

SMX

M062X/6-311+G(d,2p) EmpiricalDispersion=GD3
EE (a.u.)= -1175.59514008
B3LYP/6-31+G(d,p)
Thermal corr. to G (a.u.)= 0.164137
Gsolv(kcal/mol)= -18.83
S,0,2.2022055995,-1.9908890214,-0.2094087532
O,0,3.9485269337,2.0522431645,0.8000325865
O,0,2.0874291344,-3.0276235949,-1.244197414
O,0,2.9790351523,-2.2066664269,1.0106379633
N,0,2.8918244721,-0.6906935192,-1.1066600348
N,0,3.1028343701,0.9470511377,0.591718464
N,0,-3.2586679984,-0.1219381606,1.2634332421
C,0,0.5771010975,-1.4277938863,0.2342836407
C,0,-0.4932832257,-1.6755617776,-0.6320934744
C,0,0.3773606709,-0.7614078635,1.4477363569
C,0,3.5148350556,0.4194170447,-0.5413811517
C,0,-1.9938271918,-0.5807141787,0.9360117717
C,0,-1.7697019565,-1.2546360582,-0.2824650619
C,0,-0.9006813574,-0.342146931,1.7934077185
C,0,4.6068172981,1.1451353756,-1.1126929257
C,0,4.8244709739,2.153600944,-0.2222422726
C,0,5.7992595851,3.277727447,-0.16244008
H,0,3.3137238548,-1.0583297788,-1.9522420717
H,0,-0.3244426184,-2.2084026732,-1.56148647
H,0,1.2176794326,-0.5702404382,2.1045304418
H,0,-2.6037363331,-1.4493229128,-0.9513094858
H,0,-1.0583486833,0.1797265653,2.7333375671
H,0,5.1404194276,0.9381928945,-2.0280769757
H,0,5.2796662839,4.2415387248,-0.1406773849
H,0,6.4141698638,3.2093459792,0.7411031887
H,0,6.4557889892,3.2541853621,-1.0346448587
H,0,-3.4368406123,0.1052186077,2.2306770092
H,0,-4.045148218,-0.5377170257,0.7867434649

SMX-PhNCl
M062X/6-311+G(d,2p) EmpiricalDispersion=GD3
EE (a.u.)= -1635.14961259
B3LYP/6-31+G(d,p)
Thermal corr. to G (a.u.)= 0.152595
Gsolv(kcal/mol)= -16.74
O,0,3.0602703604,-2.116284808,1.1470351905
O,0,0.6664006569,3.2821564185,0.5655318087
O,0,1.7089792335,1.9963246308,-1.4021879901
N,0,1.9569114252,1.1905222483,0.9892161539
N,0,2.3773185665,-1.0092034147,1.6699040647
N,0,-4.1143641864,-1.0251853086,-0.7579043127
C,0,-0.5284551299,1.101494015,-0.327931276
C,0,-1.5948808443,1.3316722534,0.547451084
C,0,-0.6253129545,0.1348619306,-1.3327790212
C,0,2.4634286822,-0.0882661712,0.7312641362
C,0,-2.8691249357,-0.3905295162,-0.5922328128
C,0,-2.757789357,0.5814135019,0.4205150676
C,0,-1.7942314241,-0.6086850208,-1.4675532197
C,0,3.1642528399,-0.5467351828,-0.4233306639
C,0,3.5080375485,-1.8255803879,-0.096883756
C,0,4.255090938,-2.9000992504,-0.8066269181
H,0,-1.5157316025,2.0951167481,1.313903034
H,0,0.2013045535,-0.0191771677,-2.0170285726
H,0,-1.8756224294,-1.3539539141,-2.2497949448
H,0,3.3795605531,0.0058645806,-1.3233703025
H,0,4.5784326261,-2.5462499501,-1.7876339758
H,0,5.1384046041,-3.2017554345,-0.2340075127
H,0,3.6268228195,-3.7868419174,-0.942668181
S,0,0.9851053934,2.0354400989,-0.1310149561
H,0,1.6188348864,1.3131766834,1.940913063
H,0,-3.5888238687,0.7593224722,1.0976665337
H,0,-4.6678905026,-1.0304610581,0.094034716
Cl,0,-4.0935268625,-2.6886490892,-1.3348111362

$TS'_{PhN\text{-}Cl\text{-}1w}$

M062X/6-311+G(d,2p) EmpiricalDispersion=GD3
EE (a.u.)= -1787.90624801
B3LYP/6-31+G(d,p)
Thermal corr. to G (a.u.)= 0.189958
Gsolv(kcal/mol)= -32.13
S,0,0.6515135473,-2.8595390875,0.363657976
O,0,-2.4277903898,0.2382105487,-0.7624840832
O,0,1.1274431956,-3.7767241193,1.4038313247
O,0,0.0791312548,-3.3345060031,-0.8920965246
N,0,-0.491245809,-1.9126257584,1.2063236972
N,0,-1.2509702082,-0.4900614072,-0.5249525307
N,0,5.0926664829,0.9156516115,-0.9102530332
C,0,1.9726416744,-1.7058026269,-0.0241020336
C,0,3.0524935206,-1.5985987884,0.8579055277
C,0,1.9029172687,-0.9591696049,-1.2038184711
C,0,-1.4621367073,-1.1018254964,0.6204501944
C,0,4.0299415274,0.0297113098,-0.6267101921
C,0,4.0896961978,-0.7216282269,0.5579914999
C,0,2.9371729215,-0.0776887812,-1.499936761
C,0,-2.742723029,-0.7958257901,1.1739110574
C,0,-3.2933730887,0.0500431313,0.2569575454
C,0,-4.600009327,0.7576705694,0.1677866815
H,0,-0.7122997171,-2.3331639521,2.1015012819
H,0,3.0806051628,-2.2045291005,1.7564806708
H,0,1.0478009641,-1.0581672179,-1.8614100405
H,0,4.9428234571,-0.6083711706,1.2196887202
H,0,2.8959670457,0.5241988731,-2.4034652728
H,0,-3.1777669729,-1.1555475022,2.0939725128
H,0,-4.4510896189,1.8414603417,0.119783911
H,0,-5.1434193199,0.4543544249,-0.7331295528
H,0,-5.2135794786,0.5271669555,1.0409321879
H,0,5.1591139358,1.1941988778,-1.887162028
Cl,0,5.1514696522,2.6647009694,0.1290009163
O,0,6.989666852,3.0425092346,1.7173892116
H,0,7.560646312,3.7470755225,1.3761662777
H,0,6.0614965685,0.6993835941,-0.4354232734
O,0,7.2098650462,0.8674658785,0.5701967505
H,0,8.0665628388,0.4296219937,0.6126738226
H,0,7.2124659602,1.8656102571,1.1159644495

TS'$_{PhN\text{-}Cl\text{-}2w}$
M062X/6-311+G(d,2p) EmpiricalDispersion=GD3
EE (a.u.)= -1864.38761881
B3LYP/6-31+G(d,p)
Thermal corr. to G (a.u.)= 0.211589
Gsolv(kcal/mol)= -28.48
S,0,0.634297435,-2.8222842975,0.485764037
O,0,-2.5784082614,0.057925349,-0.8380037562
O,0,1.136523099,-3.6543876982,1.5846024835
O,0,0.0837395281,-3.4008800393,-0.7365784308
N,0,-0.5541010792,-1.872255127,1.2681765146
N,0,-1.3693637742,-0.5984221409,-0.5526945622
N,0,4.9345233739,1.0538822143,-1.0112552447
C,0,1.9080695036,-1.6449985715,0.0309553358
C,0,2.9764769103,-1.4356221192,0.907834253
C,0,1.8200019549,-0.9801147438,-1.1939266354
C,0,-1.5579138232,-1.1479906668,0.6277113477
C,0,3.9000511884,0.1386510686,-0.6708671453
C,0,3.9799892298,-0.5382049888,0.554648296
C,0,2.8217512542,-0.0766237795,-1.5391095656
C,0,-2.8555743146,-0.8696344075,1.1569353985
C,0,-3.4400493324,-0.1087803254,0.188257441
C,0,-4.7782336762,0.5292533652,0.0519438364
H,0,-0.7766173676,-2.2672148555,2.1746831713
H,0,3.0253489329,-1.9827514255,1.8425703379
H,0,0.9763718329,-1.1557535982,-1.8504431842
H,0,4.8283814664,-0.3634139212,1.2080942583
H,0,2.7624444317,0.4619211505,-2.4809275924
H,0,-3.2785251229,-1.1922200032,2.0962512527
H,0,-4.6808109198,1.6144423415,-0.0578717864
H,0,-5.2997747016,0.1490632019,-0.8326084953
H,0,-5.3869483122,0.320255043,0.9339149183
H,0,4.9668790993,1.2536733662,-2.0091041808
Cl,0,4.8470041732,2.8267285253,-0.1318546016
O,0,7.4322103316,3.0461465466,1.386345393
H,0,7.728753227,2.9475554352,2.2989788784
H,0,5.9734476709,0.861140054,-0.5375052696
O,0,7.0988552759,0.9051136538,0.269896895
H,0,7.9259636828,0.5525151584,-0.0788894961
H,0,7.2879672678,1.9354930815,0.8251064692
H,0,6.4638804987,3.84121041,1.3019694469
O,0,5.4796253017,4.4718067049,1.1285536572
H,0,5.6762799454,5.2219093888,0.5481168951

TS'$_{PhN\text{-}Cl}$
M062X/6-311+G(d,2p) EmpiricalDispersion=GD3
EE (a.u.)= -1940.834019
B3LYP/6-31+G(d,p)
Thermal corr. to G (a.u.)= 0.231092
Gsolv(kcal/mol)= -30.91
S,0,2.5023170463,-1.9237947902,0.2930513069
O,0,4.1940753942,2.252717709,0.4074758124
O,0,2.6644508496,-3.2821742102,-0.2378840148
O,0,2.696315017,-1.6145450497,1.7069303491
N,0,3.6150779354,-1.0310580116,-0.6595288329
N,0,3.3532038075,1.1416784667,0.2368646686
N,0,-2.8851929883,0.1013725989,-1.3425931287
C,0,0.8962842448,-1.3031035007,-0.207713201
C,0,0.2184824758,-1.9557149231,-1.2402741809
C,0,0.3460409787,-0.202731844,0.4539912826
C,0,4.1182251054,0.2246619922,-0.3148040494
C,0,-1.5958478298,-0.3819405536,-0.9710773866
C,0,-1.0383349356,-1.4919031956,-1.6195620115
C,0,-0.9091425096,0.2607422773,0.0674639433
C,0,5.4496189901,0.6903850172,-0.5405172016
C,0,5.4264429798,1.9687854804,-0.0664678667
C,0,6.448998573,3.0461319016,0.0337851488
H,0,0.6625421684,-2.8199637297,-1.7211696236
H,0,0.8997938799,0.2889613257,1.2447325348
H,0,-1.5895512216,-1.9984542221,-2.4069874351
H,0,-1.3562885992,1.1223288273,0.5519754919
H,0,6.2823529286,0.1543173664,-0.9700407547
H,0,6.132173462,3.934755822,-0.522233611
H,0,6.5988669301,3.3408847669,1.0776368336
H,0,7.4016803145,2.7003840271,-0.3723588238
H,0,-3.1252882362,-0.150973953,-2.2995316318
O,0,-5.2816526612,2.7748108445,0.7662324077
H,0,-5.0458698,3.2823783195,1.5519723074
H,0,-3.0688294098,1.2102224833,-1.1258721939
O,0,-3.3522885555,2.4889735446,-0.6709357267
H,0,-3.3266558566,3.2112378715,-1.3095954602
H,0,-4.276286355,2.6195642133,0.0117551128
H,0,-6.0386501771,1.8638618554,1.0840247268
O,0,-6.7780923199,0.9945211203,1.42199487
H,0,-7.6795838046,1.1998099674,1.147066703
H,0,-6.447840465,-0.2005424366,1.1303056603
O,0,-6.0906995633,-1.2509371214,0.831192204
H,0,-5.7458934101,-1.6992959005,1.6173666158
Cl,0,-4.3799684644,-0.6632597069,-0.296699551
H,0,4.2643350817,-1.6703506497,-1.1048192941

SMX-SO_2N-Cl
M062X/6-311+G(d,2p) EmpiricalDispersion=GD3
EE (a.u.)= -1635.14444272
B3LYP/6-31+G(d,p)
Thermal corr. to G (a.u.)= 0.152390
Gsolv(kcal/mol)= -15.95
S,0,0.0461043543,1.8588362281,-0.1925966018
O,0,-2.8668815414,-1.7027010662,-1.4450927263
O,0,0.6366084825,3.0843184099,-0.7174836589

O,0,-0.7899375058,1.845470153,1.0097214767
N,0,-1.0898427395,1.3336870439,-1.4657718789
N,0,-1.8893873264,-0.860403945,-1.9845226805
N,0,4.2185195672,-2.3628202747,0.2086532656
C,0,1.2988092391,0.613702143,-0.053000948
C,0,2.477054777,0.7302600855,-0.802160657
C,0,1.0949621431,-0.4858654922,0.7914588739
C,0,-1.8897454232,0.1984302842,-1.2012395292
C,0,3.2663016492,-1.3671885986,0.1422808075
C,0,3.4522726792,-0.2515929963,-0.7015261602
C,0,2.0724357098,-1.4655932798,0.8886907199
C,0,-2.8414828279,0.0918796841,-0.1392674318
C,0,-3.4144758888,-1.1255553247,-0.3476355422
C,0,-4.4818715717,-1.8925698065,0.3514777675
H,0,2.6273459031,1.5924215187,-1.4417776995
H,0,0.1866426152,-0.5582158001,1.379138888
H,0,4.3684019258,-0.1608016795,-1.2784251165
H,0,1.9184073018,-2.3163053618,1.5466552373
H,0,-3.0428238408,0.8136047085,0.6342594365
H,0,-5.3188623963,-2.0970988535,-0.3244412405
H,0,-4.1001194465,-2.85426108,0.7107177569
H,0,-4.853449396,-1.3229056294,1.2056778259
H,0,4.1810429165,-3.0141637893,0.9779266054
H,0,5.1456901228,-2.1653675103,-0.1361062088
Cl,0,-0.3329683725,1.3138453989,-3.0485046708

TS’$_{SO2N-Cl-1w}$
M062X/6-311+G(d,2p) EmpiricalDispersion=GD3
EE (a.u.)= -1787.89696962
B3LYP/6-31+G(d,p)
Thermal corr. to G (a.u.)= 0.190363
Gsolv(kcal/mol)= -29.23
O,0,-3.7137106249,2.2910818827,1.9318574989
O,0,-0.8574354518,-0.4725657852,-0.925801752
O,0,-0.6599636946,2.0796766309,-1.0220970997
N,0,-0.600516013,0.7972751784,1.3435409297
N,0,-2.3576296233,2.3713008643,1.6574129512
N,0,5.6803557188,0.3792930622,-0.6984432406
C,0,1.5305113725,0.6579877749,-0.5675040792
C,0,2.1436748763,-0.5839409997,-0.342907259
C,0,2.290272673,1.812319481,-0.8123197633
C,0,-1.9593135564,1.1192250493,1.61709076
C,0,4.3122517221,0.4761972294,-0.6325633653
C,0,3.5244835727,-0.669582397,-0.3793743584
C,0,3.6704205292,1.7165584558,-0.8526854452
C,0,-3.0108562828,0.1905855333,1.8598860826
C,0,-4.0970278813,0.9939635331,2.0473033755
C,0,-5.5338462852,0.7429501753,2.3393458847
H,0,-0.3217965925,-0.1790486204,1.7324160956
H,0,1.546643218,-1.4609251468,-0.1232881071
H,0,1.7964786407,2.7640121134,-0.9723668052
H,0,4.0071523899,-1.6263535095,-0.2033910243
H,0,4.2658915539,2.6028041187,-1.0516573208
H,0,-2.9567517098,-0.8864840974,1.8734208486
H,0,-5.7187706801,-0.3305430484,2.4119051798
H,0,-5.8254866369,1.2143477867,3.2835896385
H,0,-6.1675592926,1.1555192676,1.547445205
S,0,-0.2164196159,0.7823905656,-0.5295813732
O,0,0.6368943193,-1.1957879321,2.6000099265
H,0,1.2997084753,-0.6171931521,3.2761528413
O,0,2.028582002,0.2651049742,4.0735133708
Cl,0,0.7202426565,1.5932024782,2.507616733
H,0,2.9764449167,0.2712968201,3.8760929699
H,0,0.2932564841,-1.9498324634,3.0932652432
H,0,6.1303426442,-0.4485012871,-0.3386760916
H,0,6.233655516,1.221916804,-0.6676190497

TS’$_{SO2N-Cl-2w}$
M062X/6-311+G(d,2p) EmpiricalDispersion=GD3
EE (a.u.)= -1864.38915782
B3LYP/6-31+G(d,p)
Thermal corr. to G (a.u.)= 0.215263
Gsolv(kcal/mol)= -32.47
O,0,-3.8733050745,2.0895476956,1.1430902954
O,0,1.3053799267,0.024417419,-0.318662192
O,0,-1.0493522064,-0.1458084811,-1.2729194943
N,0,-0.6219296544,0.7169644018,1.1244156284
N,0,-2.6958616609,1.7433800056,0.4600332249
N,0,0.6089781907,6.0544265068,-2.8052736189
C,0,0.1362699521,2.2296732182,-1.1742546288
C,0,1.3439669051,2.9143992614,-0.9975330233
C,0,-0.9069031537,2.8021901486,-1.9100199903
C,0,-1.9078461166,1.2169265927,1.3768621042
C,0,0.4647754965,4.7743894543,-2.2915585952
C,0,1.504844549,4.1791414776,-1.5494856313
C,0,-0.7403199389,4.0647042822,-2.4646792075
C,0,-2.5296024832,1.2094834122,2.6697015375
C,0,-3.75066876,1.7688367304,2.4517281561
C,0,-4.9121869023,2.0825226279,3.3292794301
H,0,2.1519324978,2.4470142602,-0.4464525811
H,0,-1.8355850377,2.2598807351,-2.0374263442
H,0,2.4421089721,4.7121649836,-1.4131011317
H,0,-1.5500734021,4.5116293177,-3.0353317002
H,0,-2.1067320133,0.8800628271,3.605812985
H,0,-4.7001331228,1.7799896519,4.356905069
H,0,-5.1278370378,3.1562495891,3.3186318008
H,0,-5.8110323212,1.5584449715,2.9873003528
S,0,-0.0379467338,0.5876608909,-0.5045499259
O,0,1.7848450632,-0.7765695681,4.1197985383
H,0,2.0066219543,0.2515590023,4.1301976257
O,0,2.0657380112,1.7351136406,4.0018559719
Cl,0,0.7847205579,1.5465754751,2.4897849405
H,0,2.8623391936,2.1186317825,3.6039048301
H,0,2.6082265266,-1.2715473343,4.0177122716
H,0,0.905974893,-1.0908166377,3.2466053302
H,0,-0.2380095494,-0.5793785903,1.8612257637
O,0,0.1099605168,-1.3920431121,2.4646835755
H,0,-0.6387788304,-1.8704172793,2.8451745228
H,0,-0.0178395416,6.3273021748,-3.548236703
H,0,1.5488820344,6.4060893563,-2.9156133368

TS’$_{SO2N-Cl}$
M062X/6-311+G(d,2p) EmpiricalDispersion=GD3
EE (a.u.)= -1940.83157
B3LYP/6-31+G(d,p)
Thermal corr. to G (a.u.)= 0.238179
Gsolv(kcal/mol)= -35.77
O,0,-0.5939709993,3.5431488952,-0.1076724611
O,0,-0.0273386798,-1.9465097937,1.5859432496
O,0,-0.7569484055,0.2310705529,2.6822490349
N,0,0.4428793606,0.2055431553,0.4072647463
N,0,-0.7871982431,2.2739166071,0.4812525455
N,0,-5.94328226,-1.1569813639,-1.1602851839
C,0,-2.2103967767,-0.7587892786,0.6932999725
C,0,-2.4660298939,-1.868062058,-0.1207613366
C,0,-3.1972623113,0.2089763904,0.9089730404
C,0,0.2154833782,1.5329461872,0.0444280456
C,0,-4.7166340172,-1.041623938,-0.5210058529
C,0,-3.709574232,-2.006294898,-0.7250486658
C,0,-4.4411292571,0.0650359545,0.3061129592
C,0,1.0821242171,2.2851452075,-0.8284624633
C,0,0.5078754339,3.5209272018,-0.8847921184
C,0,0.8372215066,4.7736941169,-1.6209393549
H,0,-1.698604711,-2.6209167627,-0.2608780862
H,0,-2.983961927,1.0618695627,1.5417799757
H,0,-3.9097126584,-2.8679595479,-1.3565205069
H,0,-5.2089267836,0.8160819144,0.4725094614
H,0,1.9246362362,1.9186504312,-1.3950109375
H,0,1.7709946316,4.6532369126,-2.1740245045
H,0,0.0409447241,5.0220327368,-2.3308369639

H,0,0.9409031474,5.6174396232,-0.930695363
S,0,-0.6194584284,-0.6062941889,1.4842650706
O,0,4.109336275,-1.5231788566,-0.8066024813
H,0,3.4184461074,-1.7761778993,-1.5586676743
O,0,2.2951242753,-1.908723352,-2.5183552709
Cl,0,1.1412055268,-0.9268599166,-1.2135791208
H,0,1.8828627731,-2.782718784,-2.5939199709
H,0,4.6121226012,-2.3088695983,-0.5581076956
H,0,3.5844774842,-0.8786424823,0.1962433303
H,0,2.1247439789,-0.2351223242,0.9440837913
O,0,3.1208717023,-0.312705524,1.065066186
H,0,3.4404981043,0.6239521421,1.1705973867
H,0,-6.713573153,-0.6300912774,-0.7747107518
H,0,-6.2058498792,-2.0744240845,-1.4903258675
O,0,3.5024374616,2.3219803398,1.332264023
H,0,2.7460873859,2.6714929741,0.8282621682
H,0,3.4070373052,2.6575180233,2.2339106445

SMX-ISX-Cl
M062X/6-311+G(d,2p) EmpiricalDispersion=GD3
EE (a.u.)= -1635.11879192
B3LYP/6-31+G(d,p)
Thermal corr. to G (a.u.)= 0.151356
Gsolv(kcal/mol)= -19.11
S,0,2.0968869997,-1.4840931546,0.5627519294
O,0,5.8101499376,1.1881893687,0.6360170683
O,0,2.3928064163,-2.6323661953,-0.3050014157
O,0,2.5619833198,-1.4650056508,1.9591613867
N,0,2.58881859,-0.081234936,-0.2551797122
N,0,4.7826632076,0.2035182274,0.845851483
N,0,-3.8074508372,-0.5908186848,0.570995743
C,0,0.3336398695,-1.2182701331,0.5652002939
C,0,-0.422777214,-1.5702202753,-0.5566327754
C,0,-0.2833619021,-0.677921706,1.6962436435
C,0,3.711205509,0.5249834331,-0.0774889666
C,0,-2.4392218802,-0.8278056715,0.5816348117
C,0,-1.7985269794,-1.3749872467,-0.5484450532
C,0,-1.6605405777,-0.4841240739,1.7038720323
C,0,4.0955121374,1.7626772157,-0.7058776083
C,0,5.3228203834,2.0985522747,-0.2533210935
C,0,6.2114979689,3.2631041822,-0.503941163
H,0,0.0650544888,-2.0065560798,-1.4216042142
H,0,0.3140097201,-0.4279195981,2.5664126102
H,0,-2.3875614872,-1.6498222921,-1.4195266711
H,0,-2.1414214015,-0.0669045521,2.5847445354
H,0,3.4815929703,2.3010565945,-1.410490624
H,0,6.3758943435,3.8230326923,0.4229158177
H,0,7.1877309148,2.9269602327,-0.868509026
H,0,5.7612757244,3.9263784545,-1.2444275896
H,0,-4.2616465828,-0.4861585025,1.4668682701
H,0,-4.3570718176,-1.1072854067,-0.1005718898
Cl,0,5.5382307686,-1.3621904365,0.6220704174

$TS'_{ISX\text{-}Cl\text{-}1w}$
M062X/6-311+G(d,2p) EmpiricalDispersion=GD3
EE (a.u.)= -1787.96027564
B3LYP/6-31+G(d,p)
Thermal corr. to G (a.u.)= 0.194141
Gsolv(kcal/mol)= -21.19
S,0,0.0881481462,-1.2661758185,0.6056202049
O,0,-3.1602275249,2.3460739716,1.0199202062
O,0,0.5645226499,-2.212775053,1.6314701193
O,0,-0.7647589037,-1.741966202,-0.4965180407
N,0,-0.668937802,-0.0754958651,1.5126278463
N,0,-2.0670154632,1.7938533106,1.7096287327
N,0,4.8515451782,1.4255551694,-1.8013516782
C,0,1.5183284263,-0.4795600223,-0.1224950752
C,0,2.6241721341,-0.1735004301,0.6794940001
C,0,1.5294525116,-0.1719239761,-1.4855626094
C,0,-1.6072455002,0.7392531718,0.9815832966
C,0,3.7610757068,0.766747399,-1.2539536695
C,0,3.7335160022,0.4455970212,0.1191947272
C,0,2.6426823888,0.445654829,-2.046960512
C,0,-2.3169903354,0.7221593455,-0.2661340248
C,0,-3.2490887066,1.7096641852,-0.1706369455
C,0,-4.2990214305,2.2053326748,-1.1006134319
H,0,-0.36486213,-0.2823614769,2.8634929681
H,0,2.6189407522,-0.4301977428,1.7334513208
H,0,0.6786396221,-0.4367595573,-2.1034277303
H,0,4.5909334362,0.6822738754,0.7435735569
H,0,2.6507181644,0.6789139793,-3.1083907641
H,0,-2.1773171053,0.0190234833,-1.0694235226
H,0,-4.0961209462,3.2402777089,-1.3965561724
H,0,-4.3276320327,1.5826454124,-1.99669632
H,0,-5.2819128202,2.1812402202,-0.6191493057
H,0,5.722793414,1.3806414696,-1.2931604951
H,0,4.963070861,1.3830060854,-2.8038949493
Cl,0,-2.1422969311,1.3799707468,3.6748221974
O,0,-0.0627340679,-0.5924273094,3.9197160924
H,0,0.0265961126,-1.5576987003,3.9026055694
H,0,-0.7476598573,-0.2254389663,4.6704871856
O,0,-1.7473906464,0.4605429958,5.4106845581
H,0,-1.400718873,1.143443475,6.0054974645

$TS'_{ISX\text{-}Cl\text{-}2w}$
M062X/6-311+G(d,2p) EmpiricalDispersion=GD3
EE (a.u.)= -1864.40307717
B3LYP/6-31+G(d,p)
Thermal corr. to G (a.u.)= 0.214348
Gsolv(kcal/mol)= -27.72
S,0,0.2803081251,-1.2279271568,0.424874399
O,0,-3.157599332,2.1823631075,1.0443031982
O,0,0.8456317038,-2.1307061554,1.434806257
O,0,-0.6503316235,-1.7400770321,-0.5965358117
N,0,-0.4214395488,0.013988588,1.3158310819
N,0,-1.9648045534,1.7272630246,1.6487847806
N,0,4.8392858195,1.4409215879,-2.3818816086
C,0,1.6465944839,-0.4482739321,-0.4285379815
C,0,2.8618883892,-0.2644125716,0.2402037451
C,0,1.5003523079,-0.0266522089,-1.753276798
C,0,-1.5005171845,0.6837579096,0.8889174557
C,0,3.7923510494,0.7869598217,-1.7462310344
C,0,3.9240446279,0.3498906878,-0.4127665485
C,0,2.5644049164,0.5866841991,-2.4064707207
C,0,-2.348182243,0.5575744416,-0.2633455349
C,0,-3.3296450835,1.4852937019,-0.0993597646
C,0,-4.5041910967,1.8693928108,-0.9277419316
H,0,2.9739602011,-0.617222745,1.2598941827
H,0,0.5653246926,-0.200449314,-2.2745374887
H,0,4.8686001624,0.4881476155,0.1069712685
H,0,2.4499895866,0.9082003041,-3.4383956979
H,0,-2.2432202901,-0.163895425,-1.0551126253
H,0,-4.5798897399,1.2132416933,-1.7966759219
H,0,-5.4267144852,1.7943150035,-0.3429823284
H,0,-4.4106000608,2.904643809,-1.2732058557
H,0,5.7649193446,1.3013262948,-2.0029822142
H,0,4.817938832,1.4688270223,-3.3911311143
Cl,0,-2.2351175642,1.3475385609,3.6139310989
O,0,-0.0792444122,-0.2287892522,5.4868127035
H,0,-1.0652768072,0.2018382415,5.5904817976
O,0,-2.3211611436,0.7900826634,5.5377974452
H,0,-2.4121910711,1.6130813518,6.0409764126
H,0,-0.1350331326,-1.1894456836,5.5753979647
O,0,0.9370599508,0.4978927966,3.4163866893
H,0,0.4424635759,0.1159331975,4.4109740308
H,0,0.3219777912,0.2986245263,2.5333008998
H,0,1.0710525918,1.4543657157,3.4711533004

$TS'_{ISX\text{-}Cl}$
M062X/6-311+G(d,2p) EmpiricalDispersion=GD3

EE (a.u.)= -1940.853931
B3LYP/6-31+G(d,p)
Thermal corr. to G (a.u.)= 0.23885
Gsolv(kcal/mol)= -26.96
S,0,-0.4977037933,-0.1528143895,0.9076119377
O,0,2.3029114918,3.2536565954,-1.0888898272
O,0,-0.2153999623,-1.6165184207,0.9223637884
O,0,-0.3047984571,0.5944177807,2.1634815712
N,0,0.3979667377,0.4261163179,-0.3494692541
N,0,1.7868707683,1.9652909789,-1.3688906168
N,0,-6.2123880913,0.5104005634,-0.7190445624
C,0,-2.2076286383,0.0346056845,0.42001994
C,0,-2.7043483052,-0.6622446274,-0.6884642322
C,0,-3.0447865114,0.8734589545,1.1597498501
C,0,0.9568710608,1.6383126833,-0.3203047601
C,0,-4.8953361128,0.3295342462,-0.3213130942
C,0,-4.034884086,-0.512356377,-1.0561343533
C,0,-4.379119466,1.0190161246,0.7919837505
C,0,0.897435749,2.7529143765,0.5801627188
C,0,1.7341093509,3.6893573323,0.0552085102
C,0,2.1027234315,5.0658031039,0.4828459294
H,0,-2.0585480858,-1.318197683,-1.2625515771
H,0,-2.6519150078,1.3936208175,2.0263530059
H,0,-4.4174078216,-1.0505457544,-1.9192959882
H,0,-5.0289274952,1.6697037315,1.3712457532
H,0,0.3331623056,2.8077358069,1.4955678842
H,0,1.6283972473,5.3022762857,1.4369886894
H,0,3.1884829222,5.1546344996,0.5919589859
H,0,1.7809565711,5.7997276049,-0.2639269112
H,0,-6.6140829524,-0.1967515755,-1.3175130375
H,0,-6.8566252699,0.8706280843,-0.0300045878
Cl,0,3.3317582281,0.6627484989,-1.591486221
O,0,1.4225355573,-2.8827900418,-0.7623383148
H,0,2.5770054234,-2.7910397765,-0.6740551945
O,0,4.8009409701,-0.7022240951,-1.673128982
H,0,0.9157696385,-2.454205263,-0.0173787581
O,0,0.4354444615,-1.2552229195,-2.5544715003
H,0,1.1021215183,-2.3884443659,-1.5792694532
H,0,0.4140361233,-0.5373653068,-1.8628929744
O,0,3.8307642078,-2.7319282744,-0.6884022191
H,0,4.2683828741,-1.847536628,-1.140979314
H,0,5.0430267444,-0.7601698515,-2.6091753187
H,0,4.2489433485,-2.8944912602,0.1662072395
H,0,0.969620015,-0.9096480015,-3.2808601822

SMX-PhCCl
M062X/6-311+G(d,2p) EmpiricalDispersion=GD3
EE (a.u.)= -1635.20155894
B3LYP/6-31+G(d,p)
Thermal corr. to G (a.u.)= 0.153541
Gsolv(kcal/mol)= -16.59
O,0,3.3477725395,-1.4274649992,1.443434133
O,0,-0.1837151292,3.1244072486,0.0357213864
O,0,1.22141439,1.8597395947,-1.70824844
N,0,1.540595528,1.4835272968,0.7789307198
N,0,2.4145733256,-0.4394058452,1.7888754208
N,0,-3.6775283003,-2.4085040847,-0.6667363279
C,0,-0.7914184419,0.6215201645,-0.540670285
C,0,-1.9164716643,0.6941331827,0.2894990767
C,0,-0.6154992625,-0.4586882694,-1.4088307921
C,0,2.3307162827,0.3301438336,0.7222392373
C,0,-2.7156061378,-1.4313098473,-0.6080414048
C,0,-2.8613546105,-0.3197177821,0.2512566099
C,0,-1.5699953442,-1.4661936642,-1.4351946257
C,0,3.1618709732,-0.1214169269,-0.3454143871
C,0,3.7685410017,-1.2276270898,0.1724641486
C,0,4.7631588567,-2.1973308671,-0.3630458482
H,0,-2.055387261,1.5489277569,0.9423680572
H,0,0.2446600128,-0.5038676019,-2.0659727629
H,0,3.284169344,0.3277397318,-1.3175715782
H,0,5.0381385784,-1.92605467,-1.3842753297
H,0,5.668089727,-2.2055927696,0.2535633746
H,0,4.3531261897,-3.2128943646,-0.3681875966
S,0,0.4483572488,1.9037042707,-0.4661790003
H,0,1.138445846,1.6583008625,1.6970221503
H,0,-3.4589343566,-3.2751278316,-1.1348339231
H,0,-4.3506673505,-2.4655991386,0.0813572902
Cl,0,-1.3498709002,-2.8261806876,-2.530670398
H,0,-3.7425179251,-0.2599307229,0.883810695

SMX-Cl_{Int}
M062X/6-311+G(d,2p) EmpiricalDispersion=GD3
EE (a.u.)= -1635.14498313
B3LYP/6-31+G(d,p)
Thermal corr. to G (a.u.)= 0.152406
Gsolv(kcal/mol)= -18.99
O,0,3.2641116547,-1.3163034981,1.4361963004
O,0,-0.340576724,3.1571520022,-0.0328975548
O,0,1.1148043846,1.9541814286,-1.775929828
N,0,1.4009990157,1.5403002132,0.7130873998
N,0,2.3260535511,-0.3308128175,1.7648375309
N,0,-4.0795636561,-1.8400319175,-1.2024792416
C,0,-0.8780041895,0.6411804991,-0.6389034301
C,0,-1.8846985702,0.5561723422,0.4151033604
C,0,-0.8243382261,-0.2235859303,-1.6683204819
C,0,2.2158149417,0.4007935371,0.6752944588
C,0,-3.0187409397,-1.2140601956,-0.8476898608
C,0,-2.8844141458,-0.3466770822,0.329691259
C,0,-1.8024509851,-1.3510100253,-1.7504854842
C,0,3.0347118307,-0.0714389323,-0.3905354672
C,0,3.6623066682,-1.1543849996,0.153053911
C,0,4.6559931264,-2.1324096381,-0.3676336356
H,0,-1.8340397676,1.2536470037,1.2446309565
H,0,-0.0591600646,-0.141611155,-2.4321787588
H,0,3.1375324931,0.3464852492,-1.3786557318
H,0,4.9190775197,-1.8862642608,-1.3982415493
H,0,5.5667556008,-2.1211673773,0.240060598
H,0,4.2491968963,-3.1488817521,-0.3428088301
S,0,0.339075227,1.9642843014,-0.5373250693
H,0,1.0251592654,1.7602690529,1.6319409029
H,0,-3.6562672747,-0.3971813331,1.0927866092
H,0,-2.1301664632,-1.5492311276,-2.7705923115
Cl,0,-0.8756851124,-2.8854025844,-1.27051302
H,0,-4.8306540566,-1.7067483029,-0.5183259619

TS_Cl_1
M062X/6-311+G(d,2p) EmpiricalDispersion=GD3
EE (a.u.)= -1634.574994
B3LYP/6-31+G(d,p)
Thermal corr. to G (a.u.)= 0.137516
O,0,-3.4828460483,-1.7714959645,-1.0109353066
O,0,-0.3643364039,3.2348920815,-0.8901468643
O,0,-1.4175898843,2.2065319364,1.168542871
N,0,-1.3100589619,0.9287110988,-1.1308795969
N,0,-2.5466972819,-0.944584268,-1.648779284
N,0,4.3729397378,-0.8369528483,0.8137098117
C,0,0.8282125967,1.1803897984,0.2279874092
C,0,1.8118023085,1.1176210944,-0.8170675353
C,0,1.0398380227,0.5788205598,1.477296444
C,0,-2.2158553852,-0.0185973421,-0.7411819812
C,0,3.2617929079,-0.1781268212,0.6855555005
C,0,2.9913281289,0.4832420692,-0.5911514741
C,0,2.2383604727,-0.0597615152,1.7378065935
C,0,-2.9324033151,-0.2342106873,0.4948058681
C,0,-3.6889335363,-1.3344808807,0.2579310762
C,0,-4.6480979546,-2.1205252775,1.0839988204
H,0,1.5872669079,1.5914433667,-1.7657411196
H,0,0.2903391064,0.6945706069,2.2517888111
H,0,2.4951582946,-0.3662579896,2.7420176384
H,0,-2.8669053536,0.3615122012,1.3897369781

H,0,-4.2954449408,-3.1495620055,1.2169565807
H,0,-4.7549861135,-1.661420369,2.0694441507
H,0,-5.6341507475,-2.1635024049,0.6075133085
H,0,4.3505473915,-1.365751172,1.6961780939
S,0,-0.7047396027,2.0290550469,-0.1170397223
Cl,0,2.3789159083,-2.7675875874,2.7717986869
H,0,3.7573148359,0.4016938929,-1.3555142788

TS-NClN
M062X/6-311+G(d,2p) EmpiricalDispersion=GD3
EE (a.u.)= -1635.05881504
B3LYP/6-31+G(d,p)
Thermal corr. to G (a.u.)= 0.152252
Gsolv(kcal/mol)= -30.18
N,0,-0.3673957291,-0.7318365312,1.570378131
O,0,-0.0406042338,-3.068242494,0.3359680778
S,0,0.5301134709,-2.1257285336,1.3109886064
O,0,0.9634887205,-2.5982951202,2.6247726488
C,0,1.7901059265,-1.1620298834,0.5339434685
C,0,1.8754049892,-1.1154444198,-0.860085061
C,0,2.5052442416,-0.2351964862,1.3518451444
C,0,2.5701635794,-0.0806546,-1.4549445527
H,0,1.3260172629,-1.8311696013,-1.4596834234
C,0,3.2706137823,0.7400414224,0.7733906184
H,0,2.4184577114,-0.3111188137,2.4299014106
C,0,3.2964200382,0.8679062633,-0.6471904569
H,0,2.64230639,-0.0104029507,-2.5346701992
H,0,3.8224510987,1.4486757574,1.3827193881
N,0,4.0744626263,1.7958328241,-1.2354843432
H,0,4.4555513047,2.5476691378,-0.6783717926
H,0,3.9443256037,2.000338598,-2.216688178
C,0,-1.3721887723,-0.3688427527,0.7633642397
N,0,-1.8998857502,0.8688040523,0.9992818007
C,0,-2.0857180694,-1.0183252983,-0.3122601822
O,0,-2.9321390611,0.9912743321,0.1269548194
C,0,-3.0414004825,-0.1293476994,-0.666594627
H,0,-1.8947317897,-1.9996026647,-0.7129077554
C,0,-4.1241598692,-0.1021081503,-1.6829510293
H,0,-4.1493936411,-1.0487872448,-2.226003855
H,0,-5.0985432998,0.0600114882,-1.2102965563
H,0,-3.9578311177,0.710264901,-2.398707865
Cl,0,0.6184450596,1.8177584678,-1.1547474668

SMX_Z_enol
M062X/6-311+G(d,2p) EmpiricalDispersion=GD3
EE (a.u.)= -1175.57389244
B3LYP/6-31+G(d,p)
Thermal corr. to G (a.u.)= 0.163950
Gsolv(kcal/mol)= -15.07
C,0,2.0215559197,-0.0390090575,0.0319384728
C,0,2.3344035116,1.2710844021,-0.3471524435
C,0,3.6627043592,1.6362209342,-0.5105161732
C,0,4.6982453003,0.7016030694,-0.293360133
C,0,4.3598445204,-0.6128770458,0.0864966332
C,0,3.0304733113,-0.9819781269,0.2494712523
H,0,1.540143063,1.9869929662,-0.5255753702
H,0,3.9089799666,2.6495206717,-0.8156976783
H,0,5.1454098195,-1.3461482717,0.2463404939
H,0,2.7680874586,-1.9942201557,0.536589593
N,0,6.0178461077,1.0572940586,-0.5002896062
H,0,6.2466587898,2.0399918904,-0.5139640177
H,0,6.7343395103,0.4679110631,-0.1033325459
S,0,0.3448970013,-0.5303855126,0.2905478737
O,0,0.0223925991,0.1327089417,1.7544983513
O,0,0.277265269,-1.9923386888,0.4101117015
N,0,-0.4844114386,0.2126168537,-0.8186075448
C,0,-1.8082326798,0.5110836971,-0.5722956889
C,0,-2.7468843972,0.9652213655,-1.5524021347
C,0,-3.8912903365,1.1867081599,-0.8489483695
H,0,-2.5745976099,1.0868893702,-2.6102068962
N,0,-2.3684391205,0.4727563718,0.6322826323
O,0,-3.6946615377,0.9036219359,0.460683278
C,0,-5.2550923533,1.6499905186,-1.2271343374
H,0,-6.004287358,0.8877759831,-0.9878264774
H,0,-5.5213225301,2.5621330744,-0.682540854
H,0,-5.297178834,1.8569689477,-2.2984295686
H,0,-0.9624568321,0.3066688837,1.7641852374

SMX_Z_enol_transfer_2w
M062X/6-311+G(d,2p) EmpiricalDispersion=GD3
EE (a.u.)= -1328.45160135
B3LYP/6-31+G(d,p)
Thermal corr. to G (a.u.)= 0.203716
Gsolv(kcal/mol)= -26.30
C,0,1.7505769734,-0.227803803,0.3127060238
C,0,1.6979635849,-0.3941759678,-1.0740019544
C,0,2.6664226466,0.2018863742,-1.8729526038
C,0,3.6948969329,0.9755377462,-1.3017463395
C,0,3.7300824516,1.1315274279,0.0986495251
C,0,2.7637959874,0.5361829094,0.9007448263
H,0,0.8986383497,-0.9764921647,-1.5162718379
H,0,2.6282874114,0.0692815573,-2.9508703781
H,0,4.5270957113,1.7121906253,0.555730347
H,0,2.8041459119,0.6469689469,1.9784828081
N,0,4.6879276785,1.529971607,-2.0983111336
H,0,4.4783240732,1.6418541543,-3.0797808833
H,0,5.215016883,2.2978311031,-1.7082906299
S,0,0.4852508942,-0.979938769,1.3226517481
O,0,1.2037830557,-1.2373797602,2.6482441249
O,0,-0.0166751555,-2.1759217904,0.6345306008
N,0,-0.6610078622,0.1017811007,1.7259203005
C,0,-1.5405581234,0.5748897347,0.7628774013
C,0,-2.7145582716,1.3426577237,1.0723183375
C,0,-3.262182736,1.6195676731,-0.1417257857
H,0,-3.0832260832,1.6164456259,2.0490836574
N,0,-1.3989925283,0.4111512764,-0.5410314235
O,0,-2.5029961474,1.0795346532,-1.1200088942
C,0,-4.4804442265,2.3608736149,-0.5737317842
H,0,-5.1563005368,1.7057150199,-1.1339584919
H,0,-5.0137793193,2.7496212825,0.2965406097
H,0,-4.2155276911,3.2005019783,-1.22554635
O,0,-0.0397763436,-1.6261554023,4.6871491529
H,0,-0.6514931997,-0.6978099016,4.606390455
H,0,0.5405301714,-1.5577967484,3.7373828824
O,0,-1.2316251131,0.4182715284,4.1945423918
H,0,-0.9857929117,0.3707849663,3.1491792004
H,0,-0.8578284508,1.2252120733,4.5726089554
H,0,-0.5891320471,-2.4224682856,4.6997526714

SMX_Z_enol_transfer_3w
M062X/6-311+G(d,2p) EmpiricalDispersion=GD3
EE (a.u.)= -1404.89542582
B3LYP/6-31+G(d,p)
Thermal corr. to G (a.u.)= 0.224558
Gsolv(kcal/mol)= -28.20
C,0,-1.4772968973,-0.0575647477,-0.082291233
C,0,-2.0217539996,-1.1538007607,-0.754325089
C,0,-3.1303875998,-1.8098973858,-0.2257676207
C,0,-3.7101914866,-1.3790076001,0.9817063377
C,0,-3.148554772,-0.2687312296,1.6455551471
C,0,-2.0424559293,0.385309162,1.1191675297
H,0,-1.5771302775,-1.4828818159,-1.687017974
H,0,-3.5555097485,-2.659457349,-0.7539152454
H,0,-3.5886078007,0.0802669273,2.5760293677
H,0,-1.6176282073,1.2362637063,1.6413338014
N,0,-4.8454261504,-2.0007322598,1.4858724016
H,0,-5.0370503334,-2.9379361514,1.1624933698
H,0,-5.0461483352,-1.8641035255,2.4660347493
S,0,-0.0473805942,0.7842391894,-0.7592787893
O,0,-0.5145046181,2.2116072927,-0.9623849883

O,0,0.3451286412,0.0541718112,-1.9822101208
N,0,1.0925080671,0.9024196797,0.3610190354
C,0,1.9140736802,-0.1653281069,0.6722786296
C,0,2.1194659606,-1.4419829074,0.0442507229
C,0,3.0815021644,-2.0381341203,0.8002174634
H,0,1.6397223447,-1.8169372517,-0.8450115443
N,0,2.7075248306,-0.024747356,1.7312402461
O,0,3.4544998389,-1.2185191577,1.807517175
C,0,3.7689155202,-3.3581240642,0.7256545179
H,0,3.5888746405,-3.9438147092,1.6338178806
H,0,3.4014208799,-3.9243113941,-0.1328568714
H,0,4.851854107,-3.229477151,0.6224615223
O,0,1.0163041654,4.1270087691,-1.2696407995
H,0,0.3852303228,3.2796837399,-1.1443775047
O,0,1.8069026557,2.749414855,2.1381687041
H,0,1.344817358,2.110350831,1.5029601683
H,0,2.4145075808,2.1481002633,2.6026553841
H,0,1.2704123589,4.2006482263,-2.1986196293
O,0,2.7690174886,4.2196638202,0.3845497653
H,0,3.6163299586,3.8558144766,0.0934846701
H,0,2.4289642954,3.626968515,1.2028394464
H,0,1.9162498301,4.1742178189,-0.4976030762

SMX-imide-Z
M062X/6-311+G(d,2p) EmpiricalDispersion=GD3
EE (a.u.)= -1175.58392126
B3LYP/6-31+G(d,p)
Thermal corr. to G (a.u.)= 0.164733
Gsolv(kcal/mol)= -19.87
C,0,1.5900448612,-0.6751856833,0.2407475938
C,0,1.7887638277,-0.1427129064,-1.036405159
C,0,2.9133647605,0.6343635591,-1.2944570538
C,0,3.8554740093,0.8918549817,-0.2796055086
C,0,3.641761771,0.3417247694,1.0005592317
C,0,2.5199739418,-0.4362323291,1.2585444865
H,0,1.0745755549,-0.351860384,-1.8252601689
H,0,3.0744947643,1.0352117611,-2.2918169321
H,0,4.3688162857,0.5178630491,1.7890389129
H,0,2.3647998897,-0.8691274346,2.2409382723
N,0,5.0031170497,1.6243560497,-0.5496933453
H,0,4.9878272326,2.2251473407,-1.3608984529
H,0,5.4953807467,2.0187108911,0.2386297391
S,0,0.136253373,-1.6487137798,0.5968438656
O,0,0.4874750859,-2.6280117544,1.628803384
O,0,-0.408961042,-2.1325145634,-0.7022624282
N,0,-0.9125847884,-0.554006656,1.3108620197
C,0,-1.9217265262,-0.0714115347,0.6324122821
C,0,-2.9499333485,0.7946448302,1.1622661202
C,0,-3.8677314581,0.9716499227,0.183331667
H,0,-2.9753385998,1.1750879637,2.1709272428
N,0,-2.2342533353,-0.2236724978,-0.7095025899
O,0,-3.5144101335,0.3155381166,-0.9583666814
C,0,-5.1452329776,1.7287755362,0.1085619339
H,0,-5.0789277606,2.523226793,-0.6424219897
H,0,-5.9695885933,1.0666360486,-0.1758234672
H,0,-5.3702296825,2.1774239901,1.0776469139
H,0,-2.0628964782,-1.0972051292,-1.2024311887

$TS_{enol-Z-imide-2w}$
M062X/6-311+G(d,2p) EmpiricalDispersion=GD3
EE (a.u.)= -1328.45660020
B3LYP/6-31+G(d,p)
Thermal corr. to G (a.u.)= 0.205146
Gsolv(kcal/mol)= -23.03
C,0,1.6992847455,-0.1439567689,0.2272589967
C,0,2.6540002377,-0.9936467867,-0.3318865091
C,0,3.9560459206,-0.5383987438,-0.5242226962
C,0,4.3190327175,0.7701122663,-0.1573117211
C,0,3.3407121504,1.6118305526,0.4105701072
C,0,2.0418196446,1.1587258886,0.6028474004
H,0,2.3718531413,-2.0012655618,-0.6166762157
H,0,4.6995174573,-1.2016830475,-0.9585297746
H,0,3.6081497028,2.6230341842,0.7060727619
H,0,1.2948146033,1.8139233764,1.0376699939
N,0,5.6302524873,1.2102853651,-0.3010394382
H,0,6.2073368657,0.7119907322,-0.9634458485
H,0,5.7757528484,2.2095782475,-0.3178320703
S,0,0.0429130601,-0.7454841568,0.4949408933
O,0,-0.2764739475,-0.533857278,1.9701454237
O,0,0.0317639741,-2.2065071834,0.157718451
N,0,-0.7974709887,0.2047070008,-0.4692465003
C,0,-2.1492302896,0.1285595643,-0.5708754294
C,0,-2.893527575,0.9777334661,-1.4646405266
C,0,-4.1933061243,0.6263753793,-1.3073956598
H,0,-2.4733824826,1.7266657457,-2.1173276657
N,0,-2.9978271693,-0.6786008984,0.0753059991
O,0,-4.2994781775,-0.3656763235,-0.3906584073
C,0,-5.4672988801,1.0953446912,-1.9197476881
H,0,-6.1492343217,1.4811902327,-1.1543035901
H,0,-5.2652555601,1.8905191779,-2.6402111507
H,0,-5.975478989,0.2744883475,-2.4370737885
H,0,-3.1171903207,-1.7733432728,1.0952133062
O,0,-3.221632948,-2.5420127999,1.823567938
H,0,-2.0860152095,-2.7699707103,2.3114647682
O,0,-0.9755954683,-2.8797544556,2.6376071042
H,0,-0.6395559176,-1.9068453125,2.5843703823
H,0,-3.8874450527,-2.27052437,2.4688824127
H,0,-0.5190853046,-3.2506431183,1.8520623214

$TS_{enol-Z-imide}$
M062X/6-311+G(d,2p) EmpiricalDispersion=GD3
EE (a.u.)= -1404.89542582
B3LYP/6-31+G(d,p)
Thermal corr. to G (a.u.)= 0.224558
Gsolv(kcal/mol)= -28.20
C,0,-1.4772968973,-0.0575647477,-0.082291233
C,0,-2.0217539996,-1.1538007607,-0.754325089
C,0,-3.1303875998,-1.8098973858,-0.2257676207
C,0,-3.7101914866,-1.3790076001,0.9817063377
C,0,-3.148554772,-0.2687312296,1.6455551471
C,0,-2.0424559293,0.385309162,1.1191675297
H,0,-1.5771302775,-1.4828818159,-1.687017974
H,0,-3.5555097485,-2.659457349,-0.7539152454
H,0,-3.5886078007,0.0802669273,2.5760293677
H,0,-1.6176282073,1.2362637063,1.6413338014
N,0,-4.8454261504,-2.0007322598,1.4858724016
H,0,-5.0370503334,-2.9379361514,1.1624933698
H,0,-5.0461483352,-1.8641035255,2.4660347493
S,0,-0.0473805942,0.7842391894,-0.7592787893
O,0,-0.5145046181,2.2116072927,-0.9623849883
O,0,0.3451286412,0.0541718112,-1.9822101208
N,0,1.0925080671,0.9024196797,0.3610190354
C,0,1.9140736802,-0.1653281069,0.6722786296
C,0,2.1194659606,-1.4419829074,0.0442507229
C,0,3.0815021644,-2.0381341203,0.8002174634
H,0,1.6397223447,-1.8169372517,-0.8450115443
N,0,2.7075248306,-0.024747356,1.7312402461
O,0,3.4544998389,-1.2185191577,1.807517175
C,0,3.7689155202,-3.3581240642,0.7256545179
H,0,3.5888746405,-3.9438147092,1.6338178806
H,0,3.4014208799,-3.9243113941,-0.1328568714
H,0,4.851854107,-3.229477151,0.6224615223
O,0,1.0163041654,4.1270087691,-1.2696407995
H,0,0.3852303228,3.2796837399,-1.1443775047
O,0,1.8069026557,2.749414855,2.1381687041
H,0,1.344817358,2.110350831,1.5029601683
H,0,2.4145075808,2.1481002633,2.6026553841
H,0,1.2704123589,4.2006482263,-2.1986196293
O,0,2.7690174886,4.2196638202,0.3845497653
H,0,3.6163299586,3.8558144766,0.0934846701

H,0,2.4289642954,3.626968515,1.2028394464
H,0,1.9162498301,4.1742178189,-0.4976030762

TS'-imide-$E_{SO2N-Cl-3w}$
M062X/6-311+G(d,2p) EmpiricalDispersion=GD3
EE (a.u.)= -1940.8477967
B3LYP/6-31+G(d,p)
Thermal corr. to G (a.u.)= 0.234913
Gsolv(kcal/mol)= -26.9
O,0,-3.96662877,-0.3400950779,0.4891922225
O,0,0.5786664122,-1.4432092136,-2.1354716277
N,0,-0.4851831896,-0.3522107978,-0.0072071531
N,0,-2.7202219158,0.2879636501,0.3606971376
N,0,5.9082932502,0.3686543295,1.2561337518
C,0,2.1590286264,-0.9104489262,-0.0845096998
C,0,2.5230557855,-1.1115516558,1.2511829411
C,0,3.0324358451,-0.2792466021,-0.9739227187
C,0,-1.8104291171,-0.6598501923,0.1113919682
C,0,4.6550674675,-0.0259865165,0.8192685667
C,0,3.7603170876,-0.6698573203,1.6999087926
C,0,4.2724886997,0.158640568,-0.5243822846
C,0,-2.4739206824,-1.9357059134,0.0609141077
C,0,-3.7837217237,-1.6662247394,0.2984519991
C,0,-5.0006643884,-2.5185992257,0.3785270472
H,0,1.8440198763,-1.6165462867,1.9297453926
H,0,2.7360217474,-0.1378276384,-2.0072398266
H,0,4.0482266833,-0.83149695,2.7352134276
H,0,4.9559106825,0.6428433278,-1.2167404415
H,0,-2.0085271305,-2.8904811002,-0.1136173229
H,0,-5.7314239649,-2.2218264273,-0.3810726473
H,0,-4.7322310856,-3.5642325959,0.2179884126
H,0,-5.4781419632,-2.4234983715,1.3593713742
H,0,6.0475205904,0.4721322166,2.2503833574
H,0,6.4083738228,1.0437320596,0.696757493
S,0,0.5927958603,-1.5132431674,-0.6692297351
O,0,0.2930810003,-2.7937193388,-0.0037709065
Cl,0,-0.0761678291,1.521863023,-0.5120939327
H,0,-2.850302198,1.5678330093,0.6015811328
O,0,0.26559305,3.4838191857,-1.024396419
O,0,-3.1462997027,2.6764011364,0.8516572432
H,0,-2.5955649481,3.5018947536,0.3470443641
H,0,1.1626762845,3.6728666808,-0.7166652743
H,0,-0.9665775522,4.167074803,-0.3793417681
O,0,-1.920545937,4.4921345147,-0.2056028479
H,0,-3.1714365941,2.8319067651,1.8044407932
H,0,-2.2215314471,4.7073796636,-1.1101060916
O,0,-1.3720436319,4.5157643652,-2.9649154627
H,0,-1.6126407168,3.9610785597,-3.7157660944
H,0,-0.6033841964,4.0835243433,-2.5297943634

SMZ^-
M062X/6-311+G(d,2p) EmpiricalDispersion=GD3
EE (a.u.)= -1232.65550900
B3LYP/6-31+G(d,p)
Thermal corr. to G (a.u.)= 0.193369
Gsolv(kcal/mol)= -70.14
S,0,-0.2247059044,1.74162922,0.3314347096
O,0,0.2447791551,3.0024803377,-0.2910198522
O,0,-0.5060694484,1.781109138,1.7889481575
N,0,-1.4562216972,1.2198719562,-0.589906221
N,0,4.4424275352,-2.0268720231,-0.3972735451
C,0,1.174131916,0.5963133706,0.1242658527
C,0,1.9741122985,0.6893578243,-1.0167450535
C,0,1.4658690177,-0.3607341068,1.0964601409
C,0,3.3679704156,-1.1292066629,-0.2089311226
C,0,3.0609259828,-0.1688546448,-1.1862534732
C,0,2.5581556059,-1.214265345,0.9342764319
H,0,1.748638605,1.4482024194,-1.758970907
H,0,0.8331817036,-0.4208569396,1.9739859966
H,0,3.6796729581,-0.0919391149,-2.0786369183
H,0,2.7832877325,-1.9573936116,1.6975545721
H,0,4.8274665343,-2.3914832724,0.4651253758
H,0,5.17687961,-1.6677758775,-0.9943995076
C,0,-2.1012192183,0.0634421535,-0.3359437415
C,0,-3.88383007,-1.2822962152,-0.9350218395
C,0,-2.3823927761,-1.938993599,0.7929026156
C,0,-3.5239570436,-2.2301786055,0.0376632339
H,0,-4.0948588716,-3.1409266926,0.1896029605
N,0,-1.6784751504,-0.8229517902,0.6171611444
N,0,-3.1994059533,-0.1627890161,-1.1295552371
C,0,-1.8776066751,-2.8847994561,1.8590117014
H,0,-0.856462712,-3.2053195675,1.6238996506
H,0,-1.8404140334,-2.372599813,2.826758259
H,0,-2.5144395422,-3.7710093754,1.9498108872
C,0,-5.0894098889,-1.4922683909,-1.8247764623
H,0,-5.5977414746,-2.4370145313,-1.6045840135
H,0,-5.7997467207,-0.6682351222,-1.6951894028
H,0,-4.7842722,-1.489789196,-2.8771181126

SMZ^--PhN-Cl
M062X/6-311+G(d,2p) EmpiricalDispersion=GD3
EE (a.u.)= -1692.21017063
B3LYP/6-31+G(d,p)
Thermal corr. to G (a.u.)= 0.180349
Gsolv(kcal/mol)= -63.57
S,0,2.177984931,-2.2645533853,-0.1266336348
O,0,1.9660157245,-3.4379347242,-1.0045397496
O,0,2.7741618639,-2.5234698348,1.2068853313
N,0,2.9336937103,-1.1514801655,-1.0218975191
N,0,-3.4784701458,-0.4580370482,0.9449556519
C,0,0.4858411111,-1.6604021012,0.202492955
C,0,-0.4798817654,-1.7505753219,-0.8085241843
C,0,0.1581262282,-1.1261968369,1.4491631041
C,0,-2.1211657596,-0.7853682455,0.6852924823
C,0,-1.7802411137,-1.3179640827,-0.5692175019
C,0,-1.1405792257,-0.6748180025,1.6842650863
H,0,-0.2030785917,-2.1866569178,-1.7624849626
H,0,0.9282879034,-1.0634292542,2.2083508913
H,0,-2.5475252272,-1.4012911487,-1.3330232072
H,0,-1.4012687383,-0.2405752386,2.6479631824
H,0,-3.5814951449,-0.271166107,1.9424772228
Cl,0,-3.948802824,1.2173272132,0.2654740503
C,0,3.2597138021,0.0668718235,-0.532505545
C,0,4.404101618,2.0293489616,-0.9485124245
C,0,3.1844733994,1.7287317335,1.07554471
C,0,4.0042476794,2.5489133166,0.2934041314
H,0,4.3067316394,3.5373948712,0.6243788561
N,0,2.8109411112,0.5130106751,0.6782241019
N,0,4.045985069,0.8202025526,-1.3638831489
C,0,5.2749646474,2.8296087075,-1.8907174243
H,0,6.200245162,2.2825771885,-2.1021516566
H,0,4.7605337171,2.9711621356,-2.8476249385
H,0,5.5288965513,3.8104528688,-1.4755902478
C,0,2.6682981107,2.1794461886,2.4224984959
H,0,1.5726857696,2.2005950365,2.4177812972
H,0,2.9751470395,1.4692292914,3.1980167994
H,0,3.0383798074,3.1755353306,2.6858196255

SMZ^--$TS_{PhN-Cl-2w}$
M062X/6-311+G(d,2p) EmpiricalDispersion=GD3
EE (a.u.)= -1921.46235499
B3LYP/6-31+G(d,p)
Thermal corr. to G (a.u.)= 0.242624
Gsolv(kcal/mol)= -75.69
S,0,2.7306691619,-1.7264213326,-0.1290825485
O,0,2.6240048963,-2.925580408,-0.9897131553
O,0,3.3583133041,-1.9073186141,1.20246759
N,0,3.3526270368,-0.5523877621,-1.0408988684
N,0,-3.0539762059,-0.4870601275,1.0433869088
C,0,0.9868104239,-1.2995728802,0.2196813369

C,0,0.0136489933,-1.5501920792,-0.7538441585
C,0,0.6328552599,-0.7476284323,1.4541643957
C,0,-1.676640195,-0.7330140957,0.7522783776
C,0,-1.3253298414,-1.2629550559,-0.4958185799
C,0,-0.7029252238,-0.4606564966,1.7220481423
H,0,0.3151689877,-1.9923505597,-1.697265016
H,0,1.4115994534,-0.559926272,2.1828446743
H,0,-2.0967325853,-1.4619223641,-1.2336370141
H,0,-0.9925275035,-0.0321047382,2.6795006732
H,0,-3.2368607709,-0.5028808288,2.0447131357
Cl,0,-3.8757464802,1.191400339,0.4701057389
O,0,-6.3787414793,0.3478492905,-1.1596710894
H,0,-6.5748970813,0.2890701463,-2.1024555912
H,0,-3.7808374895,-1.0932568315,0.5048124269
O,0,-4.9551288427,-1.5273406251,-0.4022194464
H,0,-5.5097111658,-2.2919278284,-0.2126944628
H,0,-5.6027848148,-0.7032282978,-0.7575894463
H,0,-5.9110061534,1.548365317,-0.8769047136
O,0,-5.4264322954,2.5287491295,-0.5715340751
H,0,-5.9710369631,2.9160850705,0.1275892663
C,0,3.5689112926,0.6971678546,-0.5629854406
C,0,4.533070981,2.7482974134,-0.999588201
C,0,3.3434479502,2.3606892056,1.0279470414
C,0,4.0881562627,3.2412241737,0.2376237442
H,0,4.3007022813,4.2559794564,0.5584773924
N,0,3.0793325966,1.1119475505,0.6418889501
N,0,4.2840303897,1.5071803728,-1.401858913
C,0,5.327961927,3.6133118238,-1.9507355611
H,0,6.2970484432,3.1475866155,-2.1602148028
H,0,4.8007053282,3.7004793079,-2.9070573142
H,0,5.4954693898,4.6162014972,-1.5449958696
C,0,2.7868398447,2.7785921695,2.3690456646
H,0,1.6927647633,2.715593572,2.3592020056
H,0,3.1447069093,2.1006302862,3.1515035467
H,0,3.0761298345,3.8023591577,2.626354006

SMZ^{-}-$TS_{PhN\text{-}Cl}$
M062X/6-311+G(d,2p) EmpiricalDispersion=GD3
EE (a.u.)= -1997.90913169
B3LYP/6-31+G(d,p)
Thermal corr. to G (a.u.)= 0.262617
Gsolv(kcal/mol)= -77.38
S,0,2.5014432795,-1.8733109459,-0.4385837398
O,0,2.2336398316,-3.1333974563,-1.1669647692
O,0,3.2665751251,-1.9770630466,0.8275186704
N,0,3.0743353142,-0.823629561,-1.5188995466
N,0,-3.0383467468,-0.125915576,1.2053198918
C,0,0.8337937643,-1.3071469937,0.0554976094
C,0,-0.2528598074,-1.5712252079,-0.7844446327
C,0,0.6504334715,-0.6317847739,1.2652501877
C,0,-1.7146699025,-0.509377667,0.8047043039
C,0,-1.535916704,-1.1681656819,-0.4173397935
C,0,-0.6285581535,-0.2295375348,1.6425237363
H,0,-0.0837491763,-2.1122449866,-1.7092294606
H,0,1.5138375507,-0.4385096054,1.8896654517
H,0,-2.3937149975,-1.3779546707,-1.0489604178
H,0,-0.7851355662,0.2992034835,2.580543132
H,0,-3.1197015017,-0.0851992927,2.219804636
O,0,-6.7775536388,0.0507698089,-0.876040228
H,0,-6.9192135439,-0.1626080151,-1.8060888344
H,0,-3.8353637579,-0.7351131364,0.7910795701
O,0,-5.0246743039,-1.4092106365,0.103646004
H,0,-5.4749058219,-2.1657224739,0.4954120504
H,0,-5.773152552,-0.7685238418,-0.3169801556
H,0,-6.7523635561,1.2390091129,-0.7923824855
O,0,-6.7357276738,2.4696158373,-0.7481490516
H,0,-7.5296326152,2.788798564,-0.3040501281
H,0,-5.5807347716,3.1103351574,-0.3134899754
O,0,-4.6553207983,3.558700591,0.0201764799
H,0,-4.1741728904,3.8326004957,-0.772769453
Cl,0,-3.6596799926,1.6295564726,0.6733718751
C,0,3.4055574222,0.4477730151,-1.1867610297
C,0,4.4103269665,2.3929357723,-1.9177750426
C,0,3.4508205267,2.2530282106,0.2586053989
C,0,4.1393252931,3.0162737992,-0.6890753732
H,0,4.4384305288,4.0402249612,-0.4893007996
N,0,3.0820541177,0.9935389943,0.021949442
N,0,4.0549357104,1.1387619726,-2.1728228869
C,0,3.0783029398,2.8170076274,1.6098862444
H,0,1.9893146675,2.8143168553,1.7326909871
H,0,3.4966437278,2.1912166936,2.405697118
H,0,3.4421617892,3.8415872853,1.7354779315
C,0,5.1295248235,3.1238131315,-3.0283587293
H,0,4.5011532166,3.1540869242,-3.9252170576
H,0,5.3876869033,4.148037149,-2.7406760089
H,0,6.0463765023,2.5881192392,-3.2973730011

SMZ^{-}-$TS_{SO2N\text{-}Cl\text{-}2w}$
M062X/6-311+G(d,2p) EmpiricalDispersion=GD3
EE (a.u.)= -1921.40770556
B3LYP/6-31+G(d,p)
Thermal corr. to G (a.u.)= 0.244613
Gsolv(kcal/mol)= -79.37
O,0,0.6146776344,0.7651159852,2.4852122169
N,0,-4.7649670143,2.3649027671,-0.9656655782
C,0,-1.3297154899,0.6027968375,0.6820928248
C,0,-2.2908106562,-0.2037607085,0.0633525178
C,0,-1.4959983283,1.9871827977,0.7493372138
C,0,-3.6030663,1.7803839698,-0.4417573932
C,0,-3.4202393625,0.3881039675,-0.4936486331
C,0,-2.6301946965,2.5729495547,0.1934536507
H,0,-2.1666901809,-1.2804491156,0.0041847712
H,0,-0.7392448416,2.5906904461,1.2348779002
H,0,-4.1632832809,-0.2414437676,-0.976871336
H,0,-2.768782526,3.6502552644,0.2552343722
H,0,-5.2334168052,1.8164968056,-1.6747970557
H,0,-4.6721685836,3.3341036556,-1.2390040893
S,0,0.0911750872,-0.1636042136,1.4630664244
O,0,-0.3423230913,-1.5046876304,1.8752156847
N,0,1.3868063623,-0.4366036537,0.3789174197
Cl,0,0.5688597373,-1.1486490354,-1.5802456742
H,0,2.2325260251,-2.5658256541,0.052098804
O,0,1.8737498295,-3.2500428193,-0.5355230073
O,0,-0.2930507234,-4.2364919184,0.6222295463
H,0,-0.4319398556,-3.5298531847,1.2705379351
H,0,0.5552932266,-3.9309934223,0.1632758383
O,0,-2.1421172837,-3.4365076108,-1.2721807659
H,0,-1.5635950065,-3.8479783304,-0.5813734508
H,0,-1.5031931037,-2.9202961058,-1.7834450116
C,0,2.1739019737,0.6430463419,-0.0180393936
C,0,2.4671578308,2.8513708203,-0.5454232697
C,0,4.2579818472,1.2713735334,-0.7242611314
C,0,3.8040855585,2.5906125517,-0.8635887394
H,0,4.4641611965,3.3797998348,-1.2095843987
N,0,1.6491810754,1.8771897619,-0.1354191323
N,0,3.4474238121,0.3025461674,-0.2958409774
C,0,1.8725786242,4.2328485115,-0.6516183779
H,0,0.9749172946,4.20597781,-1.277395557
H,0,1.5670898833,4.586054092,0.3396384991
H,0,2.5834911547,4.9483611225,-1.0749438778
C,0,5.6761701186,0.8712803323,-1.040405982
H,0,6.2680064378,1.7189353676,-1.3982280831
H,0,6.1538518083,0.4536973132,-0.147820871
H,0,5.6773075021,0.084201679,-1.8013410128

SMZ_{iso}-$TS_{SO2N\text{-}Cl\text{-}2w}$
M062X/6-311+G(d,2p) EmpiricalDispersion=GD3
EE (a.u.)= -1922.00539021
B3LYP/6-31+G(d,p)
Thermal corr. to G (a.u.)= 0.256216

Gsolv(kcal/mol)= -30.17
S,0,0.7450704116,0.0171193118,1.7079890401
O,0,0.5668306675,1.284177879,2.4126479913
O,0,0.870232886,-1.2469148701,2.4365557021
N,0,-0.6919462544,-0.1082443937,0.7428732194
N,0,5.3594496486,0.6662492839,-2.0170950525
C,0,2.1162476158,0.1977052379,0.5861855335
C,0,2.4280677218,1.4688785536,0.0892710076
C,0,2.8899745307,-0.9135016094,0.2377322586
C,0,4.3033561741,0.5164911402,-1.1312193709
C,0,3.5104131476,1.6238015264,-0.7678702801
C,0,3.9776586279,-0.7530937897,-0.6121876618
H,0,-4.2114371826,0.477508307,1.2270137047
H,0,1.8349169982,2.3263938537,0.386795209
H,0,2.6393425711,-1.8864532281,0.6427311709
H,0,3.7537505551,2.6099182035,-1.1542397101
H,0,4.5867771842,-1.6134988026,-0.8770892902
H,0,6.0648028932,-0.0561866865,-2.0225460391
H,0,5.7308983881,1.5971068714,-2.1399839445
C,0,-1.0464817373,-1.256566544,0.090770062
C,0,-0.5556372767,-3.3305250352,-0.7670165914
C,0,-2.7680914827,-2.4439402131,-0.9971253076
C,0,-1.8684682098,-3.4716333171,-1.2432910463
H,0,-2.1802981066,-4.3566170693,-1.7842784933
N,0,-0.1523061267,-2.2242787995,-0.1343069378
N,0,-2.3502049505,-1.3580478995,-0.3086131406
O,0,-4.2988719738,0.1113717271,0.3368133266
H,0,-4.4135553113,0.9891637753,-0.3637455212
H,0,-3.171182831,-0.6002259241,0.0094696569
O,0,-4.4334493984,1.980657775,-1.217436496
H,0,-5.2300315719,2.5219038573,-1.1686983823
H,0,-3.4082656976,2.6587031585,-1.1034670091
Cl,0,-1.3843712253,1.5021642602,-0.0039429433
O,0,-2.365425937,3.1926934544,-0.9176741729
H,0,-1.9172403397,3.321782418,-1.7653746159
C,0,-4.1971269004,-2.4668022947,-1.4525507308
H,0,-4.872073764,-2.2289550337,-0.6260119503
H,0,-4.3588789721,-1.6992204238,-2.2177155752
H,0,-4.4501900024,-3.4423004989,-1.8711888475
C,0,0.4777150599,-4.4040198906,-0.9611390105
H,0,1.337527208,-3.9981186429,-1.5036853608
H,0,0.8418096977,-4.743213476,0.0143872401
H,0,0.0791205251,-5.2594115416,-1.5108370408

SMZ_{iso}
M062X/6-311+G(d,2p) EmpiricalDispersion=GD3
EE (a.u.)= -1233.18231838
B3LYP/6-31+G(d,p)
Thermal corr. to G (a.u.)= 0.207182
Gsolv(kcal/mol)= -29.73
S,0,-0.9768142732,-0.4143390495,1.8867243447
O,0,-0.9448369746,-1.7953487462,2.3847419945
O,0,-1.1739753084,0.697478457,2.8309999433
N,0,0.4814272267,-0.2483000264,1.0619945256
N,0,-5.3042259868,-0.1965914774,-2.2498758662
C,0,-2.2674943367,-0.3353230143,0.643048553
C,0,-2.5590242047,-1.4734869219,-0.1156726598
C,0,-2.9980476974,0.8407080566,0.4588018956
C,0,-4.3178171673,-0.2525878576,-1.2672006703
C,0,-3.571854941,-1.4304776737,-1.0681100038
C,0,-4.0179078639,0.8803068943,-0.487567897
H,0,-2.0055180361,-2.3902939606,0.0576262714
H,0,-2.7693001214,1.7076851807,1.0672826925
H,0,-3.7985470922,-2.3163906978,-1.6560432988
H,0,-4.5919521789,1.7934902436,-0.6248699918
H,0,-6.0065769589,0.5212422483,-2.1381547618
H,0,-5.6930749877,-1.0816024958,-2.5442038224
C,0,0.8366326391,0.8739277468,0.5039885992
C,0,0.6348277074,3.0659357844,-0.2321355945
C,0,2.710504638,1.9293612433,-0.6399481667
C,0,1.9642244546,3.0737895005,-0.7572922335
H,0,2.3729864626,3.9536975038,-1.2380203834
N,0,0.0994605201,2.0202512394,0.3647848929
N,0,2.1351743488,0.8639135517,-0.0233778985
H,0,2.6420605653,-0.0046560784,0.1076265985
C,0,4.1149774345,1.7604956279,-1.1379596398
H,0,4.7933152827,1.5167592926,-0.3123201722
H,0,4.1729449094,0.9466324674,-1.8697312016
H,0,4.4658187914,2.6777845651,-1.6131377815
C,0,-0.2184774525,4.2981378848,-0.3639472039
H,0,-0.4270084509,4.5044582908,-1.4206543408
H,0,-1.1608498963,4.1596521604,0.1662070937
H,0,0.3019199483,5.1730790602,0.0413801831

SMZ
M062X/6-311+G(d,2p) EmpiricalDispersion=GD3
EE (a.u.)= -1233.20290266
B3LYP/6-31+G(d,p)
Thermal corr. to G (a.u.)= 0.207379
Gsolv(kcal/mol)= -18.80
S,0,-0.0799131497,1.650792794,0.390243479
O,0,0.2215130099,2.9416678214,-0.2434427288
O,0,-0.499659425,1.5684171136,1.788884551
N,0,-1.320803961,1.058442983,-0.6282438036
N,0,4.5793132215,-1.9769330141,-0.4367703983
C,0,1.2987766381,0.5547434772,0.140788277
C,0,2.1457913638,0.7572462252,-0.9541643037
C,0,1.5485986912,-0.4719011217,1.0553636301
C,0,3.5102976916,-1.1192375464,-0.228775916
C,0,3.2412543311,-0.0764663746,-1.1390437688
C,0,2.6481510554,-1.3014179832,0.8710132486
H,0,-1.7058259227,1.7909698547,-1.2160339838
H,0,1.9540068463,1.5722042209,-1.6437686892
H,0,0.8839775859,-0.6092331851,1.8997170916
H,0,3.9009861646,0.0802514239,-1.9882233115
H,0,2.8459842416,-2.0992365274,1.5820262683
H,0,4.9068495997,-2.5096697259,0.3558480969
H,0,5.3182753857,-1.6575402163,-1.0460787378
C,0,-2.170145643,-0.0137632325,-0.3768644286
C,0,-2.5939358972,-1.9895480455,0.6634809564
C,0,-4.1525683576,-1.0181610124,-0.8762157043
C,0,-3.8222641515,-2.059097286,-0.000412123
H,0,-4.497638164,-2.8925235025,0.1574384846
N,0,-1.7537959388,-0.9611552009,0.4667426276
N,0,-3.3224431332,0.0177588059,-1.0619526551
C,0,-5.4447930521,-0.9935865134,-1.646270057
H,0,-6.0120721084,-0.0908627453,-1.3968176853
H,0,-5.2398819383,-0.9575847516,-2.7213848606
H,0,-6.0604656451,-1.8703648546,-1.4314683358
C,0,-2.1402778127,-3.0520423756,1.627131308
H,0,-1.1832382377,-3.4713433303,1.2995755384
H,0,-1.9794987398,-2.6126484042,2.6173437541
H,0,-2.8710022989,-3.8598826004,1.7129955897

SMZ-PhNCl
M062X/6-311+G(d,2p) EmpiricalDispersion=GD3
EE (a.u.)= -1692.75187339
B3LYP/6-31+G(d,p)
Thermal corr. to G (a.u.)= 0.194774
Gsolv(kcal/mol)= -17.11
S,0,-0.4320040416,2.1162171866,0.3541388725
O,0,-0.1655082712,3.3579067479,-0.3827990166
O,0,-0.9666134827,2.1329244091,1.7141829602
N,0,-1.5120188842,1.3150578345,-0.6975918646
N,0,4.6418531578,-1.0368372897,0.3519068718
C,0,1.0512800648,1.1202015554,0.3388801948
C,0,1.9839386413,1.3063720542,-0.6845683069
C,0,1.2756034443,0.1916055227,1.3570856161
C,0,3.3886167614,-0.3909638053,0.3302741589
C,0,3.144690046,0.5404022034,-0.6960128234

C,0,2.4460040211,-0.5612846031,1.3557016229
H,0,-1.8939839588,1.9463064884,-1.3951208193
H,0,1.801362455,2.0407812032,-1.4613253364
H,0,0.5375647451,0.0676106681,2.1400080163
H,0,3.8717792075,0.676947697,-1.4921019795
H,0,2.6309642592,-1.2793358015,2.1460868464
C,0,-2.3043502258,0.2088382595,-0.3971807956
C,0,-2.6767573557,-1.6742013995,0.8187582089
C,0,-4.1593150437,-0.9949185323,-0.9385467543
C,0,-3.8345531099,-1.9083715949,0.0711796969
H,0,-4.4614022674,-2.7711583053,0.2666829097
N,0,-1.8961747512,-0.6086886671,0.5751223572
N,0,-3.3902362829,0.0788049536,-1.1709134402
C,0,-2.2363252599,-2.5908599311,1.9269598092
H,0,-1.22817838,-2.967401087,1.724784647
H,0,-2.1927200959,-2.03893396,2.8719019151
H,0,-2.9145817103,-3.4393301577,2.0443245341
C,0,-5.3770437028,-1.1534565756,-1.8071289771
H,0,-6.0248140013,-0.2763694864,-1.707104602
H,0,-5.0811291393,-1.2161230082,-2.8595914232
H,0,-5.9472943182,-2.0477882873,-1.5449595804
Cl,0,4.6552387756,-2.7146053445,0.9057490519
H,0,5.0837118637,-1.0485872472,-0.5632814006

SMZ-TS$_{PhN-Cl-2w}$
M062X/6-311+G(d,2p) EmpiricalDispersion=GD3
EE (a.u.)= -1921.99557135
B3LYP/6-31+G(d,p)
Thermal corr. to G (a.u.)= 0.254876
Gsolv(kcal/mol)= -28.68
S,0,2.5122888936,-1.8062132481,-0.1384136661
O,0,2.4972032864,-2.8748946564,-1.1436862919
O,0,3.2509525643,-1.9437084917,1.1147745467
N,0,3.0906766838,-0.4824560028,-1.0357490244
N,0,-3.1727356905,-0.4792801172,1.1704377773
C,0,0.8149955015,-1.3767863719,0.2555036504
C,0,-0.1764063482,-1.5775643734,-0.70925964
C,0,0.5114405116,-0.8900594841,1.5273590846
C,0,-1.8144933066,-0.7828651639,0.8746000562
C,0,-1.4991813021,-1.2740041107,-0.4006938509
C,0,-0.8131081424,-0.5886142484,1.834799502
H,0,3.506623915,-0.771185929,-1.9158559143
H,0,0.08460744,-1.9814013337,-1.68120496
H,0,1.3037123748,-0.7499043238,2.2522971135
H,0,-2.2917491455,-1.4256237568,-1.1261672505
H,0,-1.0675971981,-0.200673913,2.8174702084
H,0,-3.362470273,-0.46854333,2.1706919268
Cl,0,-3.8349972449,1.2643707388,0.5043656969
O,0,-6.1194156013,0.5583997177,-1.3169263267
H,0,-6.2692812936,0.4808346287,-2.2667232957
H,0,-3.9756619699,-1.0332563516,0.5465098475
O,0,-4.9387776979,-1.3637727082,-0.393403807
H,0,-5.5413072087,-2.0905668735,-0.1963244347
H,0,-5.5311100846,-0.4479617722,-0.8594365017
H,0,-5.6271132632,1.6739094034,-1.0201771248
O,0,-5.0536143429,2.6391027298,-0.6489325535
H,0,-5.6134837278,3.1240082144,-0.0247243093
C,0,3.5290101277,0.7440774391,-0.5358925601
C,0,3.4928523208,2.3345974421,1.0849518036
C,0,4.7664656414,2.6129975549,-0.9275731519
C,0,4.3557169976,3.1167650039,0.3121833352
H,0,4.6952137681,4.0854445832,0.6612776719
N,0,3.0659779669,1.1364646405,0.651651784
N,0,4.3540326183,1.4096354904,-1.3531496375
C,0,2.989506887,2.7820153052,2.4296950356
H,0,1.895462549,2.8304880645,2.423231308
H,0,3.2786921258,2.0561354539,3.1969643244
H,0,3.384435784,3.7630678018,2.703732495
C,0,5.6859484694,3.373858409,-1.8424603825
H,0,6.5839642475,2.7820104618,-2.0475589301
H,0,5.1928239138,3.5535986691,-2.8035708016
H,0,5.9827560826,4.3328953782,-1.4114918128

SMZ-DMP-Cl
M062X/6-311+G(d,2p) EmpiricalDispersion=GD3
EE (a.u.)= -1692.72412892
B3LYP/6-31+G(d,p)
Thermal corr. to G (a.u.)= 0.194486
Gsolv(kcal/mol)= -25.91
S,0,0.5430468441,1.9007916088,0.6083499681
O,0,1.0515541264,3.1834109577,0.1097977764
O,0,0.2421592963,1.7397076085,2.0393271115
N,0,-0.8345940533,1.623164422,-0.3278997539
N,0,4.5286453462,-2.2653318064,-1.0044979517
C,0,1.7237645444,0.6409659608,0.1278337497
C,0,2.4395155681,0.7917048384,-1.0644976705
C,0,1.9547460696,-0.4602251891,0.9551999159
C,0,3.6210576961,-1.2844893345,-0.6130743345
C,0,3.3755119471,-0.1674477405,-1.4355410297
C,0,2.899500969,-1.4142236528,0.5886976117
H,0,2.2732552561,1.6662029941,-1.6846369175
H,0,1.4039859382,-0.5499911167,1.8841405971
H,0,3.9318532744,-0.0491433388,-2.3619495348
H,0,3.085972602,-2.2666302581,1.2373893997
H,0,4.9023443651,-2.8504455399,-0.2704352589
H,0,5.2273190699,-1.9872794712,-1.6793496026
C,0,-1.5814831391,0.5817094585,-0.1526910828
C,0,-3.6834513656,-0.493167566,-0.8599602354
C,0,-2.2091800404,-1.4709718921,0.773738804
C,0,-3.4105666028,-1.5171971982,0.0147792419
H,0,-4.1069562492,-2.3413354228,0.1078674755
N,0,-1.3537923258,-0.4764276302,0.682290434
N,0,-2.7731221487,0.5285942294,-0.9377014369
C,0,-1.8686780863,-2.5951922712,1.7135600926
H,0,-1.6198492908,-3.4995760277,1.1450700652
H,0,-1.0140826749,-2.3202469906,2.332038454
H,0,-2.7219768353,-2.8375643588,2.3558591945
C,0,-4.9153227481,-0.4574207702,-1.7110336957
H,0,-5.5161759954,-1.3467088379,-1.5156414032
H,0,-5.516352378,0.4317554958,-1.4964361269
H,0,-4.6586619197,-0.4286035843,-2.7746392674
Cl,0,-3.0675941595,1.8518279838,-2.0160786595

SMZ-PhCCl
M062X/6-311+G(d,2p) EmpiricalDispersion=GD3
EE (a.u.)= -1692.80438895
B3LYP/6-31+G(d,p)
Thermal corr. to G (a.u.)= 0.195914
Gsolv(kcal/mol)= -16.81
S,0,0.0012780246,1.6723102859,-1.0328384001
O,0,-0.5035765574,3.0188845985,-0.7358692569
O,0,0.5623775096,1.3356225489,-2.3399009037
N,0,1.1837910889,1.472720453,0.1826218829
N,0,-4.2902074533,-2.2614622606,0.2781023867
C,0,-1.274954834,0.492343888,-0.6404959321
C,0,-2.2754162145,0.8371645658,0.2741217406
C,0,-1.2784634303,-0.7520061227,-1.2715603569
C,0,-3.3145611984,-1.3477646281,-0.0478524501
C,0,-3.2786345227,-0.0759557197,0.5639487672
C,0,-2.2899676828,-1.6532056521,-0.9713484056
H,0,1.4270195884,2.3545928165,0.6225798953
H,0,-2.2712265445,1.8168912707,0.7386132732
H,0,-0.5019981047,-1.0056818835,-1.9818429026
H,0,-4.4209363843,-3.0546844647,-0.332353985
C,0,2.1593160851,0.4811032278,0.2460372264
C,0,2.8825738101,-1.6065087324,-0.2879158125
C,0,4.1702683788,-0.1454033722,1.1102021546
C,0,4.0370086608,-1.381984518,0.467970606
H,0,4.8052617918,-2.1421685746,0.5544943703
N,0,1.9275586341,-0.6678843854,-0.3920940976

N,0,3.2253117943,0.7987764311,0.993190716
C,0,2.6397726783,-2.8967904473,-1.0215867875
H,0,1.6930582472,-3.3416642676,-0.6983935372
H,0,2.5551598228,-2.7039167471,-2.0964270132
H,0,3.4457837983,-3.6152519948,-0.8544241839
C,0,5.3677632835,0.199578811,1.9527011102
H,0,5.8642185288,1.0890761096,1.5511055349
H,0,5.0513012092,0.4410964896,2.972838065
H,0,6.0868769697,-0.622216523,1.9893145603
H,0,-5.1239295181,-1.9188753488,0.7303735464
Cl,0,-2.3049612423,-3.2225485494,-1.7734683542
H,0,-4.0594320371,0.1865476156,1.272437613

SMZ-Cl_{Int}
M062X/6-311+G(d,2p) EmpiricalDispersion=GD3
EE (a.u.)= -1692.74922867
B3LYP/6-31+G(d,p)
Thermal corr. to G (a.u.)= 0.194855
Gsolv(kcal/mol)= -19.11
S,0,-0.0853233055,1.7037714021,-1.1132515506
O,0,-0.6078869518,3.0418193396,-0.8116993715
O,0,0.4569360228,1.3731840247,-2.4275642454
N,0,1.0720561916,1.4816890875,0.1093102155
N,0,-4.8061204634,-1.6363817317,-0.2329777161
C,0,-1.3640365094,0.5122007783,-0.6995889094
C,0,-2.2006743908,0.7917614137,0.462814737
C,0,-1.5291708213,-0.5706658778,-1.4777264926
C,0,-3.6540113657,-1.0765701051,-0.1732033125
C,0,-3.2718709383,0.0151522112,0.7309589581
C,0,-2.5994479199,-1.5624064406,-1.1559747732
H,0,1.321685243,2.3569029292,0.5603787992
H,0,-1.9587735424,1.6483137815,1.0826252326
H,0,-0.8693138083,-0.762617246,-2.3160665832
C,0,2.0537154893,0.4844455198,0.1520483402
C,0,2.7680314651,-1.5911116659,-0.4260465091
C,0,4.0554205543,-0.1650466084,1.0114390708
C,0,3.918097231,-1.3866434108,0.343179231
H,0,4.6815524843,-2.1527328661,0.4195106647
N,0,1.818877335,-0.6441581089,-0.514466168
N,0,3.1132529298,0.7861905234,0.9096436298
C,0,2.5239276019,-2.8605563213,-1.1926069477
H,0,1.5440256027,-3.2723767754,-0.9323602305
H,0,2.5085913639,-2.6475673511,-2.2672728744
H,0,3.2950588477,-3.6085667435,-0.9939372238
C,0,5.2497918044,0.1573111807,1.8663589905
H,0,5.7492219844,1.0554940995,1.4885380444
H,0,4.9298495008,0.3742414053,2.8909409132
H,0,5.9672252832,-0.6663654841,1.8857319151
H,0,-3.0892560274,-1.9555846594,-2.046352907
H,0,-3.9199809686,0.2305560142,1.5761248009
Cl,0,-1.7542511702,-3.0479044779,-0.4242756271
H,0,-5.4337789325,-1.2575688468,0.4830198493

SMZ-TS-Cl_1
M062X/6-311+G(d,2p) EmpiricalDispersion=GD3
EE (a.u.)= -1692.164194
B3LYP/6-31+G(d,p)
Thermal corr. to G (a.u.)= 0.170061
O,0,-0.4617866314,2.7834404941,-1.0080206187
O,0,-1.3167834307,1.9727886071,1.2316756644
N,0,-1.0518859083,0.3171828431,-0.8393834703
N,0,4.8173886249,-0.244409758,1.1259791889
C,0,0.9903458408,1.0607840578,0.3536951631
C,0,1.9139943054,0.8364315315,-0.723487078
C,0,1.3537113249,0.864405552,1.69301989
C,0,3.6167343543,0.2065444865,0.9307983614
C,0,3.1848672348,0.4478871094,-0.446777072
C,0,2.6440427604,0.4761454514,2.0041866752
H,0,1.5673869123,0.9890628385,-1.738728776
H,0,0.6301505402,1.0890988442,2.4686493687
H,0,2.996032099,0.4844851837,3.0262930501
H,0,4.9181122209,-0.515259007,2.114474117
S,0,-0.6524067733,1.6737007274,-0.0503069446
Cl,0,3.314231571,-1.8147299051,3.6754175949
H,0,3.9126627986,0.2520705286,-1.2275865904
C,0,-2.181415237,-0.4124908545,-0.6094171757
N,0,-3.2740375502,0.1531588775,-0.0396573132
N,0,-2.1192244803,-1.6961595098,-1.0551688623
C,0,-4.3522842087,-0.6153739515,0.1202553225
C,0,-3.2026616259,-2.4528297408,-0.8886979639
C,0,-4.3682907854,-1.9545904516,-0.2881415447
H,0,-5.2460993059,-2.5780939771,-0.1523909687
C,0,-5.5511483798,0.0414437027,0.7581256457
H,0,-6.3842081073,-0.658371931,0.8743957486
H,0,-5.2768058514,0.4405374826,1.7400115075
H,0,-5.88057293,0.8902068593,0.1490922198
C,0,-3.1073254617,-3.877812068,-1.3763538376
H,0,-2.312568549,-4.4041407076,-0.8369581544
H,0,-4.0481248852,-4.4187113157,-1.2367900515
H,0,-2.8396483562,-3.89343168,-2.438099916

SMZ-TS-NClN
M062X/6-311+G(d,2p) EmpiricalDispersion=GD3
EE (a.u.)= -1692.65026980
B3LYP/6-31+G(d,p)
Thermal corr. to G (a.u.)= 0.192745
Gsolv(kcal/mol)= -35.37
N,0,-1.411804673,0.9581081143,-0.8492128079
O,0,-1.2592225017,2.2941218623,1.4714804297
S,0,-0.7352891061,2.1236140131,0.114615945
O,0,-0.5426247944,3.3002084217,-0.7381433094
C,0,0.7744710144,1.1951150514,0.1450085586
C,0,1.078446608,0.3690647511,1.2407615627
C,0,1.6115672781,1.2286927049,-1.0180645941
C,0,2.2210635466,-0.3967035952,1.2094056775
H,0,0.4043464155,0.3423216951,2.0881403871
C,0,2.7633825813,0.5014972319,-1.0516454348
H,0,1.3142405329,1.8573314916,-1.8497990213
C,0,3.1350485484,-0.3054028542,0.0821045131
H,0,2.5153593887,-0.9958749303,2.0634885448
H,0,3.4257319948,0.5376334555,-1.910590739
N,0,4.4005246572,-0.733760831,0.2077439565
H,0,4.9890831042,-0.765858463,-0.6139336593
H,0,4.6161843206,-1.4189061384,0.9201172117
Cl,0,1.9230426367,-2.5665036162,-0.33722601
C,0,-2.0873781572,-0.14266009,-0.3888783231
C,0,-2.7954743611,-1.5578644235,1.2705366708
C,0,-3.4015015816,-1.9095277085,-1.0334277188
C,0,-3.490500219,-2.308907553,0.3091878286
H,0,-4.0625397624,-3.1853873591,0.5944530495
N,0,-2.6900568203,-0.8369866191,-1.3846316774
N,0,-2.098929795,-0.4748213361,0.9227758968
C,0,-2.7815567936,-1.9416382326,2.7257248082
H,0,-3.5543149618,-2.6790124166,2.9573823775
H,0,-1.8076219463,-2.3724991974,2.9860964051
H,0,-2.9260735518,-1.0544547514,3.3480238739
C,0,-4.0820248451,-2.6663588709,-2.1419923913
H,0,-3.3356294965,-3.0208423855,-2.8601720577
H,0,-4.6458352388,-3.5224630381,-1.7633126288
H,0,-4.7619608814,-2.0023258329,-2.685000064

STZ^-
M062X/6-311+G(d,2p) EmpiricalDispersion=GD3
EE (a.u.)= -1458.76803409
B3LYP/6-31+G(d,p)
Thermal corr. to G (a.u.)= 0.122874
Gsolv(kcal/mol)= -63.34
S,0,2.5703943256,-1.7993563049,-0.1691181082
O,0,2.431534118,-2.8806116325,-1.1716250664
O,0,3.1968210207,-2.1515160493,1.1272286097

N,0,3.2805025371,-0.5541527908,-0.9401914714
N,0,3.2639373318,1.0401011969,0.903602497
N,0,-3.1048720063,-0.1581484069,1.0730625896
C,0,0.8666034542,-1.3033774089,0.2189122614
C,0,-0.1590366417,-1.605985861,-0.6792931056
C,0,0.5737753607,-0.6266045214,1.4056212936
C,0,3.5667165091,0.5897111642,-0.3046644939
C,0,-1.7816929641,-0.5677240116,0.8005773873
C,0,-1.4747444958,-1.2410302928,-0.39264948
C,0,-0.7419406763,-0.263272538,1.6933408206
C,0,4.454713574,2.8931213486,0.0984137288
H,0,0.083421509,-2.1425854247,-1.5906406229
H,0,1.380987593,-0.383716897,2.0864302489
H,0,-2.2703921981,-1.480845641,-1.0958147613
H,0,-0.9657107176,0.2670754132,2.6172578152
H,0,-3.3042232277,-0.0396646947,2.0583303734
H,0,-3.813075631,-0.7275251935,0.6272140322
S,0,4.5251040903,1.7938256177,-1.2644812797
H,0,4.9121556416,3.8725368719,0.0745894649
C,0,3.7587721923,2.3042647503,1.114148721
H,0,3.5727493011,2.7770573063,2.0746385459

STZ^{-}-PhNCl
M062X/6-311+G(d,2p) EmpiricalDispersion=GD3
EE (a.u.)= -1918.32256896
B3LYP/6-31+G(d,p)
Thermal corr. to G (a.u.)= 0.109854
Gsolv(kcal/mol)= -56.61
S,0,2.5621492636,-1.7842061482,-0.1898838712
O,0,2.4169277278,-2.859573945,-1.1949571114
O,0,3.1857683067,-2.1394491236,1.1051330269
N,0,3.2419091009,-0.525194365,-0.9512143094
N,0,3.2666119094,1.0412951065,0.9159766867
N,0,-3.1329741112,-0.315487841,1.1379875895
C,0,0.8523608732,-1.2938654695,0.2085202586
C,0,-0.1782836617,-1.6165242851,-0.6840678388
C,0,0.578930289,-0.5993279553,1.3897299393
C,0,3.5549429463,0.6075847921,-0.3004847862
C,0,-1.777050766,-0.5670737382,0.7982314977
C,0,-1.4906965717,-1.2624296618,-0.3879830428
C,0,-0.7332217572,-0.2188426002,1.6703726474
C,0,4.4813215824,2.884770286,0.129905456
H,0,0.0658754197,-2.1682665526,-1.5854513247
H,0,1.3958505124,-0.3432762583,2.0536922431
H,0,-2.3055134628,-1.5279957535,-1.0547742927
H,0,-0.9532686578,0.3439787794,2.5756995887
H,0,-3.1671428144,0.0423878245,2.0925615211
Cl,0,-3.836430999,1.1496376278,0.2249156939
S,0,4.5217170897,1.8056154297,-1.2478871554
H,0,4.9539021328,3.8570130781,0.1147913092
C,0,3.7843100208,2.2940692173,1.1435242658
H,0,3.6132527871,2.7551529858,2.1120319286

STZ^{-}-TS$_{PhN-Cl-2w}$
M062X/6-311+G(d,2p) EmpiricalDispersion=GD3
EE (a.u.)= -2147.57443890
B3LYP/6-31+G(d,p)
Thermal corr. to G (a.u.)= 0.171901
Gsolv(kcal/mol)= -68.58
S,0,2.4223674637,-2.0141486037,-0.2527411484
O,0,2.199714277,-3.1157531939,-1.2127142855
O,0,3.0337723404,-2.3546709428,1.0514411115
N,0,3.1558980796,-0.8231343721,-1.0637628521
N,0,3.3061607418,0.8027055428,0.7459163968
N,0,-3.1482768078,-0.1627565617,1.1069920518
C,0,0.7455838069,-1.4165104397,0.1471274154
C,0,-0.3224223112,-1.7618903541,-0.6884253304
C,0,0.5378600991,-0.6260295261,1.2837910308
C,0,3.5571201188,0.3074950338,-0.4549788429
C,0,-1.8203278457,-0.5623306215,0.7641826935
C,0,-1.6139573569,-1.335996223,-0.3848737331
C,0,-0.7488798728,-0.1903883975,1.5869638355
C,0,4.6493771821,2.5209633131,-0.1084496596
H,0,-0.1269747133,-2.3828805803,-1.5558300769
H,0,1.3864857082,-0.3547014274,1.9003764342
H,0,-2.4582760012,-1.6076894129,-1.0111849883
H,0,-0.9243956607,0.4303586797,2.4632009084
H,0,-3.2360397368,0.0441501797,2.0998718794
Cl,0,-3.9308671036,1.4150904756,0.2522882663
O,0,-6.6182461674,0.4285891117,-0.9468072218
H,0,-6.9106375944,0.1870899019,-1.8338968253
H,0,-3.9546220648,-0.8128465927,0.7704391355
O,0,-5.228922302,-1.3436886628,0.074728044
H,0,-5.8028736913,-2.0072551117,0.4727078218
H,0,-5.863672487,-0.5671860635,-0.3943115407
H,0,-6.0617791579,1.6251782424,-0.9707432884
O,0,-5.49834955,2.6092112429,-0.9263504638
H,0,-5.9514507864,3.1749213761,-0.2859577802
S,0,4.6021404618,1.3936871058,-1.4453704593
H,0,5.1909740649,3.455033944,-0.1615614577
C,0,3.9164651151,2.0212402096,0.9282877004
H,0,3.7847489718,2.5281143984,1.8796628789

STZ^{-}-TS$_{PhN-Cl}$
M062X/6-311+G(d,2p) EmpiricalDispersion=GD3
EE (a.u.)= -2224.02108330
B3LYP/6-31+G(d,p)
Thermal corr. to G (a.u.)= 0.192023
Gsolv(kcal/mol)= -70.34
S,0,2.4266660476,-1.9948715087,-0.7154541285
O,0,2.0780229873,-3.1112536836,-1.6193776928
O,0,3.1725117888,-2.3209027837,0.5206243555
N,0,3.085773068,-0.8362522165,-1.6302480567
N,0,-2.926720123,0.0410879656,1.1764238717
C,0,0.8156238021,-1.3532835518,-0.1468393578
C,0,-0.3394317149,-1.667246384,-0.8704545584
C,0,0.7454744024,-0.5568334502,1.0019887263
C,0,-1.6474306662,-0.4158542891,0.7145633353
C,0,-1.5809682821,-1.1983760234,-0.4439073352
C,0,-0.4913105953,-0.0807963058,1.4296447633
H,0,-0.2506655248,-2.2969292175,-1.7490452597
H,0,1.658591714,-0.3121249877,1.5311249842
H,0,-2.4909207755,-1.4482248036,-0.9807968217
H,0,-0.5599699742,0.547397528,2.3152403689
H,0,-2.9208847748,0.2008309896,2.1823242043
O,0,-6.8014998446,0.0481274571,-0.6505622768
H,0,-7.0055460224,-0.2728480994,-1.5370701408
H,0,-3.7630885611,-0.5933641887,0.9021305131
O,0,-5.0100233583,-1.3197215817,0.3892929494
H,0,-5.4454647691,-2.0058491053,0.9070962706
H,0,-5.7751284687,-0.7180702793,-0.0590312299
H,0,-6.7576292898,1.2386477792,-0.7122916597
O,0,-6.7249221744,2.463622614,-0.8164256179
H,0,-7.4859411938,2.8468033421,-0.3653759747
H,0,-5.5389223944,3.1282330196,-0.5276305976
O,0,-4.5903561565,3.5955982184,-0.301815715
H,0,-4.1567186197,3.7768459829,-1.147057915
Cl,0,-3.5698124477,1.7380859287,0.4931841052
C,0,3.5595933005,0.3037980324,-1.0951719682
S,0,4.4996061838,1.35723204,-2.2175786944
C,0,4.0776922321,2.0448540659,0.2007557571
C,0,4.6980857681,2.5140938613,-0.9201197696
H,0,4.052864993,2.5741511793,1.1486753956
H,0,5.2383715413,3.4409905882,-1.0526889507
N,0,3.4429371322,0.8280885475,0.1136497509

STZ
M062X/6-311+G(d,2p) EmpiricalDispersion=GD3
EE (a.u.)= -1459.29735960
B3LYP/6-31+G(d,p)

Thermal corr. to G (a.u.)= 0.135176
Gsolv(kcal/mol)= -17.11
S,0,2.2281968829,-1.9356709087,-0.2491495068
O,0,2.1063552833,-2.9515456,-1.302870239
O,0,3.0344136865,-2.158619084,0.9495481141
N,0,2.8911238377,-0.6040071688,-1.142861059
N,0,-3.225187857,-0.1667839422,1.3579092865
C,0,0.6061384336,-1.4006858048,0.2324152489
C,0,-0.4746289083,-1.6332882961,-0.6252496028
C,0,0.4185927081,-0.772789803,1.4680162552
C,0,-1.9617182959,-0.6014223931,0.997505779
C,0,-1.7493918402,-1.2355205492,-0.2443304706
C,0,-0.857784642,-0.3778751637,1.8457613919
H,0,3.2724932018,-0.9497319734,-2.017322962
H,0,-0.3155177266,-2.1363851563,-1.5727273958
H,0,1.2676955821,-0.594795755,2.1168705982
H,0,-2.5913958477,-1.4181765486,-0.906429506
H,0,-1.0071071253,0.1118777036,2.8041669846
H,0,-3.392399604,0.0304740069,2.333537298
H,0,-4.0155078473,-0.5697709426,0.8765849756
C,0,3.5290176556,0.473923466,-0.5459721332
S,0,4.9499799627,1.209190382,-1.2993597769
C,0,3.9701256249,2.0916230327,0.8832558577
C,0,4.9792581809,2.3630598339,0.0086863351
H,0,3.7703764009,2.6552705216,1.7865313755
H,0,5.7136775605,3.154800578,0.0396437314
N,0,3.146775723,1.0321628048,0.5643666205

STZ-PhN-Cl
M062X/6-311+G(d,2p) EmpiricalDispersion=GD3
EE (a.u.)= -1918.84603874
B3LYP/6-31+G(d,p)
Thermal corr. to G (a.u.)= 0.122450
Gsolv(kcal/mol)= -15.44
S,0,-0.0718935929,1.5469356811,0.4228927429
O,0,0.1671067789,2.8685134017,-0.1694434398
O,0,-0.5136104996,1.383372062,1.8054534751
N,0,-1.2689965618,0.8998774116,-0.6462319198
N,0,4.8531105922,-1.715726574,-0.4158635362
C,0,1.366565729,0.5287096612,0.1531559819
C,0,2.2850335405,0.8993212412,-0.8326992608
C,0,1.5649607517,-0.6127119028,0.933399022
C,0,3.6311787137,-1.0344508094,-0.2553025096
C,0,3.4108981654,0.1114258535,-1.0428302623
C,0,2.6990565481,-1.3924788943,0.731124553
H,0,-1.6741335065,1.65583284,-1.1887232247
H,0,2.118607207,1.792616949,-1.4243327256
H,0,0.8347654426,-0.8866013687,1.6851863238
H,0,4.1293850812,0.3932287971,-1.8078244483
H,0,2.8613697714,-2.2782482363,1.3336379554
C,0,-2.0942601272,-0.1662734885,-0.3099531317
S,0,-3.799877763,-0.1684044808,-0.7700346321
C,0,-2.7415906388,-2.1095558424,0.5006840923
C,0,-3.9401852888,-1.7433030748,-0.0346594532
H,0,-2.558742269,-3.0413577034,1.0218412019
H,0,-4.8721975739,-2.2893134802,-0.0562208539
N,0,-1.698073184,-1.2231202252,0.3351437005
Cl,0,4.8277220098,-3.4634974554,-0.1874946046
H,0,5.2678903542,-1.560929242,-1.3304004964

STZ-TS'$_{PhN-Cl-2w}$
M062X/6-311+G(d,2p) EmpiricalDispersion=GD3
EE (a.u.)= -2148.08930492
B3LYP/6-31+G(d,p)
Thermal corr. to G (a.u.)= 0.182480
Gsolv(kcal/mol)= -26.74
S,0,2.2335959952,-1.9311831107,-0.248127707
O,0,2.1046119454,-2.9518537261,-1.2934038848
O,0,3.0039917173,-2.1563860107,0.9712817326
N,0,2.9034122833,-0.6067893807,-1.122253303
N,0,-3.2670739025,-0.1472362984,1.2663491357
C,0,0.5937863896,-1.3713954154,0.2074589946
C,0,-0.476876847,-1.6546487082,-0.6453882165
C,0,0.4142604512,-0.6897688165,1.4127483074
C,0,-1.9508024882,-0.557604652,0.9201686227
C,0,-1.7578030665,-1.2448138878,-0.2870104997
C,0,-0.8677789452,-0.2762972515,1.7641104928
H,0,3.2374703129,-0.9241519099,-2.0261272622
H,0,-0.3076517209,-2.2033918064,-1.5649909742
H,0,1.2647228729,-0.4777962227,2.0489992331
H,0,-2.6107616216,-1.4601747047,-0.9220959575
H,0,-1.0257361758,0.2667049085,2.6918970051
H,0,-3.366137414,0.0517394398,2.2599034533
Cl,0,-3.9381601541,1.4782598282,0.345520887
O,0,-6.397303795,0.5579647442,-1.1119321385
H,0,-6.6348904864,0.3180216437,-2.0156039401
H,0,-4.1436231862,-0.7675629997,0.8172581725
O,0,-5.188155409,-1.2150732992,0.0423990227
H,0,-5.7867042339,-1.8694767464,0.4214256772
H,0,-5.7986611791,-0.3705931805,-0.5320715912
H,0,-5.8493414015,1.6798234896,-1.0674380005
O,0,-5.2104792377,2.6710195546,-0.9301159658
H,0,-5.6911063171,3.2902491899,-0.3609687329
C,0,3.5530615727,0.4652559126,-0.5216823858
S,0,4.9814158166,1.1857078537,-1.2674520438
C,0,4.0104033122,2.0658532949,0.9202102584
C,0,5.0227051943,2.3303688201,0.0471903162
H,0,3.8161326187,2.6265224988,1.8263248946
H,0,5.7650230769,3.114580117,0.0818122289
N,0,3.1747050524,1.0184100717,0.5922333687

STZ-TS'$_{SO2N-Cl-2w}$
M062X/6-311+G(d,2p) EmpiricalDispersion=GD3
EE (a.u.)= -2148.09289174
B3LYP/6-31+G(d,p)
Thermal corr. to G (a.u.)= 0.186078
Gsolv(kcal/mol)= -31.43
O,0,-0.0381105892,-1.871889373,1.8014850137
O,0,-0.4675961322,0.4831171723,2.6849847548
N,0,0.9763294227,0.0671669376,0.586250718
N,0,0.1341591721,2.2931697866,0.1390920845
N,0,-5.4316261907,-0.3083440531,-1.5753239916
C,0,-1.8579858468,-0.3931540239,0.6024041972
C,0,-2.257482724,-1.5231485528,-0.1188542755
C,0,-2.6538293635,0.7568767187,0.621346129
C,0,1.0900513814,1.4170797484,0.2870712037
C,0,-4.2634056393,-0.3486878467,-0.8287824886
C,0,-3.4500278011,-1.4992485341,-0.8319658756
C,0,-3.8472365353,0.7762273983,-0.0885389507
H,0,-1.6437207137,-2.4162590516,-0.1015867648
H,0,-2.3328342038,1.622627585,1.1867625201
H,0,-3.7615613273,-2.3770569669,-1.3919885463
H,0,-4.4668308088,1.6690360662,-0.0746202719
S,0,-0.3581721472,-0.4573137654,1.5637812447
O,0,3.6232571122,-2.9592624493,-0.4592294327
H,0,3.020322942,-2.8750930714,-1.3089270241
O,0,2.0806141502,-2.4773227755,-2.418649786
Cl,0,1.2872349001,-1.1830323319,-1.1568216157
H,0,1.3516860617,-3.0743057329,-2.6481595467
H,0,3.5686700829,-3.8646544604,-0.1259718092
H,0,3.3466751167,-2.096709529,0.4280157494
H,0,2.2577946913,-0.7400582989,1.0714421764
O,0,3.1244358582,-1.311687709,1.2572346384
H,0,3.8725309543,-0.7182913422,1.4125439942
H,0,-6.112962565,0.3948329991,-1.3289444124
H,0,-5.8387004356,-1.1918045598,-1.8457141495
S,0,2.7187214711,2.0866597608,0.0474329635
C,0,0.6382789231,3.5357978041,-0.1601107346
C,0,1.996769456,3.6349267068,-0.2640255269
H,0,2.5951745018,4.4983939594,-0.5170629357

H,0,-0.0503162845,4.3593234043,-0.3077112691

STZ-THZ-Cl
M062X/6-311+G(d,2p) EmpiricalDispersion=GD3
EE (a.u.)= -1918.84047763
B3LYP/6-31+G(d,p)
Thermal corr. to G (a.u.)= 0.122807
Gsolv(kcal/mol)= -17.20
S,0,-0.041856311,1.0978526116,0.479289726
O,0,-0.0345277384,2.4344692764,-0.1235801039
O,0,-0.3648427977,0.9409751444,1.9154606966
N,0,-1.0720912949,0.1433975825,-0.4389510722
N,0,-3.0040270651,-1.1365635357,-0.6733130677
N,0,5.2319040098,-1.4936932176,-0.5684041165
C,0,1.5390630413,0.334668127,0.1785137362
C,0,2.2655059312,0.6800076518,-0.9655064467
C,0,2.0453244567,-0.6008057223,1.085389696
C,0,-2.1289910965,-0.3832751545,0.0836918828
C,0,4.0231231759,-0.8650278848,-0.3045294422
C,0,3.4969556664,0.0828674474,-1.2053836631
C,0,3.2791407056,-1.1952730576,0.845488646
C,0,-4.157486763,-1.3420177863,1.2750965361
H,0,1.8706146889,1.4214260569,-1.6516389377
H,0,1.4814971442,-0.8438118492,1.9796183921
H,0,4.0623592565,0.3512960466,-2.0938767908
H,0,3.6754869195,-1.9189491209,1.5528735482
H,0,5.7058264806,-1.9265205639,0.2111288739
H,0,5.8560893154,-1.0371363077,-1.2176963405
S,0,-2.7784648003,-0.3529884405,1.7553569269
H,0,-4.9113365157,-1.6229084698,1.9957790773
C,0,-4.1236168537,-1.667402496,-0.0267815978
H,0,-4.8210874322,-2.2567753054,-0.6029559558
Cl,0,-2.7350796636,-1.3974000524,-2.3449877433

STZ-Cl$_{Int}$
M062X/6-311+G(d,2p) EmpiricalDispersion=GD3
EE (a.u.)= -1918.84134430
B3LYP/6-31+G(d,p)
Thermal corr. to G (a.u.)= 0.121766
Gsolv(kcal/mol)= -18.51
S,0,1.0273275723,-1.7674200897,0.2307005246
O,0,1.1145563224,-3.0579015507,-0.4617319648
O,0,1.4619636649,-1.5976760152,1.6135090335
N,0,1.9168178735,-0.7327307713,-0.81825783
N,0,-4.6969542313,-0.6170203685,-0.0158231618
C,0,-0.6525382452,-1.1601350095,0.0707093065
C,0,-1.3577611686,-1.4313153477,-1.177525413
C,0,-1.2102493284,-0.5046709804,1.1028691914
C,0,-3.4220704703,-0.5991786581,-0.1503035293
C,0,-2.6691019445,-1.1329917146,-1.2923983256
C,0,-2.6152078789,-0.0052039222,0.994249962
H,0,2.5044404493,-1.2966558479,-1.4246625493
H,0,-0.8145655087,-1.8980656557,-1.9918888495
H,0,-0.6461377038,-0.2888040791,2.0032215313
H,0,-3.2134403514,-1.3551086007,-2.2061970637
H,0,-5.1472253235,-1.0099287918,-0.8482058082
C,0,2.414314248,0.5045400535,-0.4074628659
S,0,4.0377672604,1.0276671686,-0.8578848894
C,0,2.4856715127,2.4859969731,0.546623444
C,0,3.7303606282,2.5187874513,-0.0090178584
H,0,2.0491195747,3.2856357603,1.1321993428
H,0,4.4651683441,3.3106375101,0.0114679602
N,0,1.7409936624,1.3500575292,0.3112001499
H,0,-3.1712249924,-0.119294187,1.9245148147
Cl,0,-2.5092571758,1.8294219741,0.7552532579

STZ-TS$_{Cl1}$
M062X/6-311+G(d,2p) EmpiricalDispersion=GD3
EE (a.u.)= -1918.280235
B3LYP/6-31+G(d,p)
Thermal corr. to G (a.u.)= 0.100597
O,0,-0.4000739271,3.2992956461,-0.5781362377
O,0,-1.3273532731,2.096924116,1.4561544968
N,0,-1.3645695884,1.0048928897,-0.9165171441
N,0,4.479286298,-0.8035973826,0.5592274127
C,0,0.8738252933,1.1786005272,0.3138634407
C,0,1.7915568296,1.2001877662,-0.7881979918
C,0,1.1629440629,0.4967184647,1.503579204
C,0,3.3454469189,-0.1682381479,0.5364723532
C,0,2.9905692112,0.5698937471,-0.6742463666
C,0,2.3807815742,-0.1462200547,1.6466933332
H,0,1.5094694401,1.7337107441,-1.6886932945
H,0,0.4563255576,0.5410053095,2.3244979964
H,0,2.6936351468,-0.5262233072,2.6087917868
H,0,4.518797084,-1.3902421435,1.4028973899
S,0,-0.6743928944,2.0398252816,0.1297906695
Cl,0,2.5798499646,-2.9363069555,2.4166652611
H,0,3.7115215393,0.5549037572,-1.4851519613
C,0,-2.3234094445,0.1263406447,-0.5881331443
N,0,-2.7324502442,-0.800662612,-1.4492169484
C,0,-4.1344970893,-1.2760434509,0.3496157718
C,0,-3.7240589685,-1.5694409579,-0.9293847216
H,0,-4.8952432915,-1.775034058,0.9346584633
H,0,-4.1367430869,-2.3685151408,-1.5361096723
S,0,-3.2339971326,0.0586974669,0.983202703

STZ-PhCCl
M062X/6-311+G(d,2p) EmpiricalDispersion=GD3
EE (a.u.)= -1918.89801626
B3LYP/6-31+G(d,p)
Thermal corr. to G (a.u.)= 0.123608
Gsolv(kcal/mol)= -15.38
S,0,1.0551075929,-1.8072744766,0.1576581292
O,0,1.2169611933,-3.0623442902,-0.5863127246
O,0,1.4700705437,-1.6726658942,1.5519265123
N,0,1.9662919541,-0.7090976048,-0.8221572073
N,0,-4.5935470551,0.0129586003,-0.3770834571
C,0,-0.6290358165,-1.26031164,0.0009070333
C,0,-1.4245668206,-1.7685108436,-1.0313444162
C,0,-1.1417079121,-0.344036039,0.9213338848
C,0,-3.2992780611,-0.4242656216,-0.2346107002
C,0,-2.7425953839,-1.3517225987,-1.1421683269
C,0,-2.4620919104,0.0611443739,0.7962722941
H,0,2.5792067128,-1.2358659735,-1.4356970735
H,0,-1.0148323354,-2.492805643,-1.7262274585
H,0,-0.5165353289,0.0478491935,1.7137711512
H,0,-3.3675566193,-1.7442229133,-1.9394188268
H,0,-5.2169174073,-0.5233769396,-0.9602644636
C,0,2.408329314,0.5276454033,-0.371313164
S,0,3.9894894342,1.1521610962,-0.8530161971
C,0,2.4156593067,2.4772542163,0.6546559747
C,0,3.6379789371,2.5941597176,0.0638793636
H,0,1.9613281357,3.2314812045,1.2855452591
H,0,4.3346081946,3.4192910445,0.0961720796
N,0,1.7186742883,1.3145172393,0.4001425199
H,0,-5.0223411789,0.5015800771,0.3948094933
Cl,0,-3.1204889579,1.2073355615,1.9591173007

STZ-TS-NClN
M062X/6-311+G(d,2p) EmpiricalDispersion=GD3
EE (a.u.)= -1918.77368413
B3LYP/6-31+G(d,p)
Thermal corr. to G (a.u.)= 0.122458
Gsolv(kcal/mol)= -27.85
N,0,-0.7649742102,1.1388963447,-1.0230718026
O,0,-0.7205463498,2.2024449135,1.3812224951
S,0,0.0023710652,2.1537800361,0.0983572697
O,0,0.3194091322,3.3817033815,-0.6246195997
C,0,1.4102307067,1.1072123283,0.2574211278
C,0,1.524406257,0.2793903943,1.3754629717

C,0,2.2415756469,0.9312345527,-0.8790947521
C,0,2.4088022681,-0.7828210131,1.3389748169
H,0,0.8734130288,0.4259933942,2.2287368605
C,0,3.1681250036,-0.0831422569,-0.8962677229
H,0,2.1257846479,1.590727055,-1.732039849
C,0,3.2436317584,-0.9903162187,0.1942829167
H,0,2.5001873008,-1.4506350661,2.1882744588
H,0,3.809030367,-0.2292318788,-1.7601436292
N,0,4.1351549122,-2.0099620609,0.1847528876
H,0,4.5796476656,-2.2628942946,-0.6862235272
H,0,4.0134620005,-2.7667952654,0.8431557755
C,0,-1.7162333662,0.3095614975,-0.7144804808
N,0,-2.2387871151,-0.5485684103,-1.6297972421
C,0,-3.5935199228,-0.9907437864,0.209052808
Cl,0,0.5053359171,-2.454429512,-0.0116698277
S,0,-2.5852687848,0.1731103959,0.912163636
C,0,-3.2546649381,-1.2402582207,-1.1335378636
H,0,-4.3860189166,-1.4698857919,0.770039902
H,0,-3.7690583246,-1.9613975482,-1.7586291093

[i] Cao, X.; Mo, Y.; Zhang, F.; Zhou, Y.; Dong Liu, Y.; Zhong, R. Reaction sites of pyrimidine bases and nucleosides during chlorination: A computational study. Chemosphere 2024, 358, 142189.

[ii] Gallard, H.; von Gunten, Urs. Chlorination of Phenols: Kinetics and Formation of Chloroform, Eviron. Sci. Technol. 2002, 36, 884-890.
